# Phytochemical Investigation of *Celtis Mildbraedii* Engl and Exploratory *In Vitro* and *In Silico* Evaluation against *Schistosoma Mansoni*

**DOI:** 10.1021/acsomega.6c05025

**Published:** 2026-09-25

**Authors:** Fabiola Madaha Tanekou, Lucas Fukui-Silva, Monique C. Amaro, Johane K. Dongmo Jumeta, Diderot Noungoue Tchamo, Josué de Moraes

**Affiliations:** † Department of Organic Chemistry, Faculty of Science, 107751 University of Yaounde I, P.O. Box 812, Yaounde, Cameroon; ‡ Research Center on Neglected Diseases, 92928 Guarulhos University, Guarulhos, São Paulo 07023-070, Brazil; § Research Center on Neglected Diseases, Scientific and Technological Institute, Brazil University, São Paulo, São Paulo 08230-030, Brazil

## Abstract

Schistosomiasis remains a major neglected tropical disease, underscoring the need to expand the chemical space available for antischistosomal drug discovery. Using modern methods in phytochemistry, a chemical investigation of the stem bark and roots of *Celtis mildbraedii* Engl. (Cannabaceae) led to the isolation and structural characterization of 17 secondary metabolites, including a previously unreported dicarboxylic acid (**1**) named celtic acid, a previously unreported paraconic acid (**2**) named milbraedic acid, a monounsaturated fatty acid (**3**) (9*R*,10*E*,12*R*,13*S*)-9,12,13-trihydroxy-10-octadecanoic acid, reported for the first time from natural sources, and its previously unreported hydrogen-bonded dimer (**4**), named Niemeyong acid dimer, together with 13 known compounds (**5**–**17)**, belonging to several classes of secondary metabolites, including triterpenoids, xanthones, phenolic amides, phenolic acids, alkaloids and fatty acid derivatives. The isolated metabolites were subjected to exploratory *in vitro* phenotypic screening against adult worms and newly transformed schistosomula of *Schistosoma mansoni*. Three compounds, including corosolic acid (**11**), compound **3**, and gallic acid (**14**), exhibited measurable activity against adult worms, with EC_50_ values of 158.6, 302.6, and 764.21 μM, respectively. No activity was observed against larval stages. The active metabolites did not display cytotoxicity in mammalian cell lines or lethality in *Caenorhabditis elegans* at the highest tested concentrations (2000 μM). *In silico* analyses indicated generally acceptable drug-likeness profiles, predicted gastrointestinal absorption, and low acute oral toxicity in rats. Although the antiparasitic potency was modest, the combined chemical and biological evaluation expanded the repertoire of natural products assessed against *S. mansoni* and provides a foundation for future structure-based optimization.

## Graphical abstract

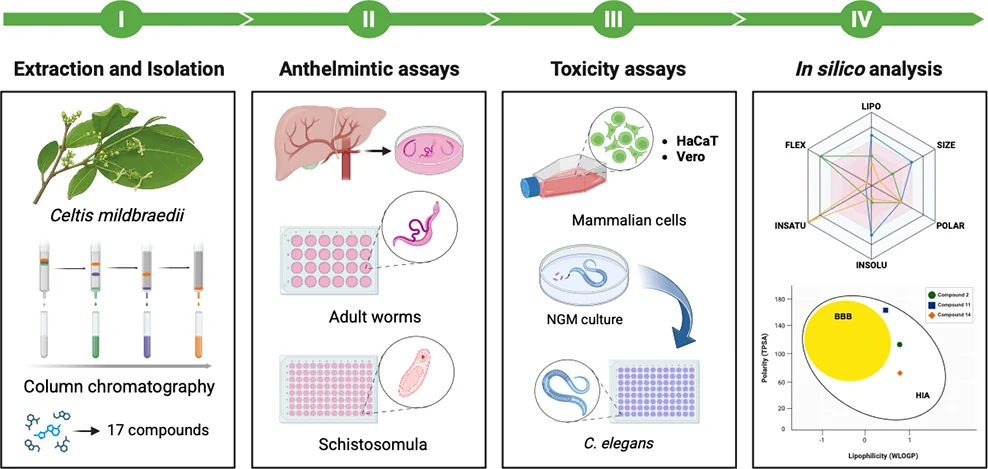

## 1 Introduction

Schistosomiasis is a parasitic disease caused by blood flukes of the genus *Schistosoma* and remains one of the most prevalent neglected tropical diseases worldwide.^1^ More than 250 million individuals are currently infected, with the highest burden concentrated in sub-Saharan Africa. Chronic infection contributes to anemia, impaired growth, hepatic fibrosis, and long-term organ damage, generating substantial social and economic consequences in endemic regions.^2^ The persistence of schistosomiasis reflects structural inequities in access to sanitation, safe water, and health services, challenges directly connected to the United Nations Sustainable Development Goals, particularly SDG 3, which aims to ensure healthy lives and well-being, and SDG 6, which promotes access to clean water and sanitation.^3^

In response to the sustained global burden of schistosomiasis and other helminthiasis, the World Health Organization established the Roadmap for Neglected Tropical Diseases 2021–2030, which defines targets for control and elimination as public health problems.^3^ Achieving these goals requires not only preventive chemotherapy, sanitation improvements, and vector control, but also continued innovation in therapeutics.^4^ The roadmap emphasizes the importance of strengthening the drug development pipeline for neglected tropical diseases, including the discovery of new antihelminthic agents capable of complementing existing treatments.^5^ This need is particularly relevant given that praziquantel, the cornerstone of current chemotherapy, is mainly effective against adult worms, shows limited activity against juvenile stages, and does not prevent reinfection.^6^

Natural products remain a major source of chemically diverse bioactive molecules. Many antiparasitic agents have originated from natural scaffolds or were inspired by natural molecular architectures.^7^ In helminth research, plant-derived metabolites such as terpenoids, alkaloids, phenolic compounds, and oxygenated fatty acids have demonstrated *in vitro* activity against *Schistosoma* species.^8,9^ Even when displaying moderate potency, such compounds expand chemical diversity and provide structural templates for future optimization and mechanistic studies.

The genus *Celtis* (Cannabaceae) comprises approximately 100 species distributed in tropical and subtropical regions and is recognized for producing structurally diverse secondary metabolites, including flavonoids, triterpenes, phenolic amides, and fatty acid derivatives.^10^ *Celtis mildbraedii* Engl, widely distributed in Central and West Africa, is traditionally used in ethnomedicine for gastrointestinal disorders and helminth-related conditions.^11^ Despite this traditional relevance, the phytochemical profile of the stem bark and roots of *C. mildbraedii* has not been comprehensively investigated, and its potential activity against *Schistosoma* species remains largely unexplored. The investigation of biodiversity from endemic regions represents an important strategy for expanding the chemical space available for antihelminthic discovery, particularly when phytochemical expertise is integrated with advanced biological screening platforms.

In this study, we performed a phytochemical investigation of the stem bark and roots of *C. mildbraedii*, leading to the isolation and structural characterization of 17 secondary metabolites, including previously undescribed compounds. The isolated metabolites were subjected to an exploratory *in vitro* phenotypic screening at a fixed concentration against adult worms and newly transformed schistosomula of *Schistosoma mansoni*. Cytotoxicity assays in mammalian cell lines and complementary *in silico* analyses were conducted to provide preliminary safety and physicochemical context. This work represents the first integrated chemical and exploratory biological evaluation of the stem bark and roots of *C. mildbraedii* in the framework of antischistosomal research aligned with global efforts to expand therapeutic options for neglected tropical diseases.

## 2 Materials and Methods

### 2.1 General Experimental Procedures

^1^H and ^13^C NMR spectra were recorded on Bruker Avance DRX-500 spectrometers operating at 500 and 600 MHz for ^1^H and at 125 and 150 MHz for ^13^C, respectively. Chemical shifts are reported in ppm (δ) with reference to residual solvent signals. Infrared spectra were obtained using a Thermo Electron Nicolet 380 FT-IR spectrometer. High-resolution mass spectra (HRMS) were recorded on an Agilent 6520 Accurate-Mass Q-TOF LC/MS instrument (Agilent Technologies). Column chromatography (CC) was performed on silica gel (Merck, 230–400 mesh) using mixtures of *n*-hexane, ethyl acetate (EtOAc), and methanol (MeOH) as eluents. Flash chromatography (FC) was also conducted on silica gel under similar solvent systems. Thin-layer chromatography (TLC) analyses were carried out on Merck silica gel 60 F254 aluminum plates (0.5 mm thickness). Spots were visualized under UV light at 254 and 366 nm (VL-4-LC UV lamp) and by exposure to iodine vapor or by spraying with H_2_SO_4_/H_2_O (1:9, v/v) followed by heating at 100 °C for 2 or 3 min. Optical rotation was measured using a P-2000 Obéron polarimeter. Melting points were recorded using BUCHIR SMP-20 (maximal temperature 300 °C, precision ±1.0 °C).

### 2.2 Plant Material

The stem bark and roots of *C. mildbraedii* were harvested in November 2020 at Niemeyong (3°22′ N, 11°15′ E), Centre Region, Cameroon. The plant material was identified at the National Herbarium of Yaoundé, where voucher specimens (Nos. 2819 SRFK and 3910 SRFK) were deposited.

The collected material was cut into small pieces, washed with distilled water, and air-dried at room temperature under shade. The dried plant material was pulverized using an electric grinder and stored in sealed containers until extraction.

### 2.3 Extraction and Isolation

The pulverized stem bark (2.0 kg) and roots (2.5 kg) were extracted using EtOH/H_2_O (80:20, v/v) by maceration for 72 h. The filtrate was concentrated using a rotary evaporator and freed from solvent to afford 300 g of stem bark extract and 200 g for root extract.

270 g of the stem bark extract was subjected to Flash Chromatography (FC) on silica gel eluting with *n*-hexane, mixtures of *n*-hexane/EtOAc (1:0 to 0:1, v/v) to EtOAc/MeOH (95:5 to 90:10, v/v). A total of 60 fractions of 500 mL each were collected. Using their TLC profiles, those fractions were combined on three fractions F1 (120.4 g), F2 (80.5 g) and F3 (50.8 g). Fraction F1 (120.4 g) was subjected to Chromatography Column (CC) on silica gel, eluted with *n*-hexane/EtOAc mixtures (90:10 to 30:70, v/v) and yielded compound **1** (20 mg), a mixture of **5** and **6** (15.5 mg), **7** (10.2 mg), **8** (15.4 mg), **9** (7 mg). Fraction F2 (80.5 g) was also subjected to CC on silica gel and eluted with mixtures of n-Hex/EtOAc (50:50, v/v) to EtOAc/MeOH (95:5, v/v) mixtures to afford compound **2** (10 mg) and **10** (10.3 mg). Fraction F3 (50.8 g) was too complex and was not studied.

The same protocol was done for 150 g of the root extract and 50 fractions of 500 mL each were collected and grouped using their TLC profiles into three fractions F1 (40.2 g), F2 (40.7 g) and F3 (30.8 g). Fraction F1 (40.2 g) was subjected to CC on silica gel, eluted with *n*-hexane/EtOAc mixtures (95:5 to 70:30, v/v) and yielded the mixture of **15**, **16** and **17** (20.2 mg). Fraction F2 (40.7 g) was also subjected to CC on silica gel and eluted with *n*-hexane/EtOAc mixtures (80:20 to 50:50, v/v) to give compound **11** (10.5 mg), **12** (8.3 mg), **13** (11 mg) and **14** (10 mg). Fraction F3 (30.8 g) was also subjected to CC on silica gel and eluted with EtOAc/MeOH mixtures (100:0 to 75:15, v/v) to afford compound **3** (15 mg) and **4** (17.2 mg).

**Celtic acid (1).** White needle; mp: 124–126 °C; [α]_D_:^25^ + 4.7 (c 0.15, (DCM/MeOH 50:50); IR υ_max_ 3329, 3209, 2954, 2849, 1690 cm^–1^; NMR data (Table 1); HR-ESI-MS [M-H]^−^ at *m*/*z* 327.2542 (calcd for C_19_H_35_O_4_^–^ 327.2535). **Milbraedic acid** (**2**). White powder; mp: 132–134 °C; IR υ_max_ 3060, 2954, 2849, 1737, 1702 cm^–1^; NMR data (Table 1); HR-ESI-MS [M + H]^+^ at *m*/*z* 327.2519 (calcd for C_19_H_35_O_4_^+^ 327.2535). **(9** *R*,**10** *E*,**12** *R*,**13** *S* **)-9,12,13-trihydroxy-10-octadecanoic acid (3).** White needle; mp: 106–108 °C; [α]_D_:^25^ −2.8 (c 0.30, (DCM/MeOH 1:1); IR υ_max_ 3278, 3200, 2952, 2848, 1697, 1468 cm^–1^; NMR data (Table 2); HR-ESI-MS [M + Na]^+^ at *m*/*z* 353.2314 (calcd for C_18_H_34_O_5_Na^+^ 353.2304). **Niemeyong acid dimer** (**Dimer of (9** *R*,**10** *E*,**12** *R*,**13** *S* **)-9,12,13-trihydroxy-10-octadecanoic acid**) **(4).** White powder; mp: 116–118 °C; [α]_D_:^25^ −10.22 (c 0.20, (MeOH); IR υ_max_ 3270, 3196, 2952, 2848, 1697, 1468 cm^–1^; NMR data (Table 2); HR-ESI-MS [M + Na]^+^ at *m*/*z* 683.4777 (calcd for 683.4710).

**Table 1:** ^1^H NMR (500 MHz) and ^13^C NMR (125 MHz) Data for **1** (CDCl_3_/CD_3_OD) and **2** (CDCl_3_)

	**1**			**2**		
No	*δ*c type	*δ*_H_ (*J* in Hz)	COSY	*δ*c type	*δ*_H_ (*J* in Hz)	COSY
**1**	177.7, C	/	/	/	/	/
**2**	48.8, CH	2.51 (m)	H-3, H-5	177.4, C	/	/
**3**	42.2, CH	2.51 (m)	H-2, H-6	36.4, CH	3.02 (1H, dq, *J* = 9.9, 7.2)	H-4, H–CH2
**4**	176.9, C	/		51.6, CH	3.20 (1H, dd, *J* = 9.7, 8.2)	/
**5**	15.0, CH_3_	1.10 (d, *J* = 6.3)	H-2	77.2, CH	4.68 (1H, td, *J* = 9.2, 8.9, 3.6)	H-4, H-6*a*/6b
**6**	30.8, CH_2_	1.54 (m)	H-3	31.2, CH_2_	1.37 (1H, m, Ha)/1.54 (2H, m, Hb)	/
		1.39 (m)				
7–16	27.4–30.8 CH_2_	1.18 (m)	/	22.7–31.9 CH_2_	1.24 (22H, m)	/
**17**	31.9	1.18 (m)	/	14.1	0.86 (3H, t, *J* = 6.9)	/
**18**	22.6	1.18 (m)	/	/	/	/
**19**	13.6, CH_3_	3.77 (t, *J* = 6.9)	/	/	/	/
**–CH**_**3**_	/	/	/	14.4	1.30 (3H, d, *J* = 7.2)	/
–COOH	/	/	/	174.5	/	/

**Table 2:** ^1^H NMR (500 MHz) and ^13^C NMR (125 MHz) Data for **3** (C_5_D_5_N) and **4** (CD_3_OD)

	**3**			**4**		
No	δc type	δ_H_ (*J* in Hz)	COSY	δc type	δ_H_ (*J* in Hz)	COSY
**1**	177.3	/	/	177.9		/
**2**	35.2	2.56 (dt, *J* = 7.9, 4.1)	/	35.1	2.29 (t, *J* = 7.4)	/
**3**	27.1	1.85 (m)	/	30.4	1.34 (m)	/
**4**	31.1	1.43 (m)	/	26.3	1.60 (m)	/
**5**	30.9	1.45 (m)	/	30.8	1.60 (m)	/
**6**	26.9	1.86 (m)	/	30.3	1.36 (m)	/
**7**	27.4	1.89 (m)	/	26.4	1.60 (m)	/
**8**	39.9	1.90 (m)	H-9	38.5	1.52 (m)	H-9
		1.83 (m)				
**9**	73.2	4.60 (m)	H-8	73.3	4.05 (q, *J* = 6.0)	H-8
**10**	137.6	6.42 (ddd, *J* = 15.7, 10.0, 6.2)	H-11, H-9	136.6	5.71 (m)	H-11, H-9
**11**	132.1	6.58 (ddd, *J* = 15.8, 10.0, 5.9)	H-10, H-12	131.1	5.71 (m)	H-10, H-12
**12**	77.5	4.69 (m)	H-13, H-11	76.7	3.93 (t, *J* = 5.0)	H-13, H-11
**13**	76.5	4.15 (m)	H-14	75.8	3.48 (dt, *J* = 8.4, 3.7)	H-14
**14**	34.7	1.99 (m)	H-13	33.6	1.54 (m)	H-13
**15**	27.6	1.66 (m)	/	26.8	1.54 (m)	/
**16**	33.6	1.37 (m)	/	33.3	1.34 (m)	/
**17**	24.3	1.37 (m)	/	23.9	1.34 (m)	/

All compounds evaluated in the biological assays had a purity >95%. Individual purity values, molecular weights, and the corresponding molar concentrations at the fixed screening concentration of 200 μg/mL are provided in Table S1 of the Supporting Information.

### 2.4 Parasite Maintenance

*Schistosoma mansoni* (BH strain) was maintained using *Biomphalaria glabrata* snails as intermediate hosts and Swiss mice as definitive hosts at Research Center on Neglected Diseases (Guarulhos University, Brazil). Adult worms were recovered by perfusion of infected mice at 7 weeks postinfection.^12^

Newly transformed schistosomula (NTS) were obtained by mechanical transformation of cercariae and maintained in RPMI 1640 medium supplemented with 10% fetal bovine serum, 100 U/mL penicillin, and 100 μg/mL streptomycin at 37 °C in a 5% CO_2_ atmosphere.^12^

All animal procedures were conducted in accordance with institutional and national guidelines for the care and use of laboratory animals and were approved by the Institutional Animal Ethics Committee under protocol number CEUA 65/24.

### 2.5 *In Vitro* Screening against Adult Worms

Adult worm pairs of *S. mansoni* were transferred to 24-well culture plates containing 1 mL of complete RPMI 1640 medium supplemented with 10% fetal bovine serum, 100 U/mL penicillin, and 100 μg/mL streptomycin.^13^ Each well contained two worm pairs. Compounds (**1**–**17**) were initially screened at a fixed concentration of 200 μg/mL. Test solutions were prepared in DMSO, with the final solvent concentration maintained at or below 0.5% (v/v). Praziquantel (2 μM) was included as a positive control, whereas culture medium containing 0.5% (v/v) DMSO was used as the vehicle control.

Parasites were incubated at 37 °C in a humidified atmosphere containing 5% CO_2_ and monitored for 72 h. Worm viability was assessed by microscopic examination of motility and morphological integrity. For the purposes of the phenotypic assay, worms were considered dead when they showed complete absence of spontaneous movement for a minimum of 60 s and remained unresponsive to gentle mechanical stimulation using a fine forceps.^14^ Experiments were performed in triplicate and repeated in three independent biological replicates.

Compounds that induced measurable effects under these conditions were further evaluated in concentration–response assays, starting at 200 μg/mL and using 2-fold serial dilutions, to determine EC_50_ values. Parasites were exposed to each concentration under the same experimental conditions described above and evaluated after 72 h.

### 2.6 Screening against Schistosomula

Newly transformed schistosomula of *S. mansoni* were obtained from cercariae shed by infected *B. glabrata* snails following 3 h of light exposure at 28 °C. Cercariae were mechanically transformed into schistosomula and maintained in Medium 199 supplemented with 100 U/mL penicillin and 100 μg/mL streptomycin at 37 °C in a humidified atmosphere containing 5% CO_2_ for 24 h prior to the screening assay.^12^

For phenotypic evaluation, approximately 50 schistosomula were transferred into each well of 96-well culture plates containing 200 μL of complete medium. Compounds were tested at an initial concentration of 200 μg/mL. Praziquantel (10 μM) was used as a positive control, while medium containing 0.5% (v/v) DMSO served as the negative control.^15^

Plates were incubated at 37 °C under 5% CO_2_, and parasite viability was assessed at 1, 24, 48, and 72 h. Effects were evaluated by microscopic observation of motility, morphological integrity, and mortality.^16^ For quantitative analysis, schistosomula were classified as viable or nonviable based on these phenotypic parameters, and mortality was expressed as the percentage of nonviable parasites relative to the total number of schistosomula evaluated in each well. All experiments were conducted in triplicate and repeated in three independent biological replicates.

### 2.7 Cytotoxicity Assays

Cytotoxicity was evaluated in Vero (African green monkey kidney epithelial) and HaCaT (human keratinocyte) cell lines cultured in DMEM supplemented with 10% fetal bovine serum at 37 °C in 5% CO_2_. Cells were seeded into 96-well plates and allowed to adhere for 24 h prior to treatment.^17^ Compounds were tested at six 2-fold serial dilutions ranging from 2000 to 62.5 μM and incubated for 72 h. The final concentration of DMSO did not exceed 0.5% (v/v) and showed no effect on cell viability. Cell viability was determined using the MTT assay. Absorbance was measured at 570 nm using a microplate reader. CC_50_ values were calculated by nonlinear regression analysis.^18^

### 2.8 *Caenorhabditis Elegans* Assay

*Caenorhabditis elegans* (Bristol N2 strain) was maintained on nematode growth medium seeded with *Escherichia coli* OP50 at 21 °C.^19,20^ Synchronized L4-stage worms were transferred to 96-well plates and exposed to compounds for 24 h at 21 °C. Worm viability was assessed based on motility and response to mechanical stimulation.^21^ Experiments were performed in triplicate.

### 2.9 *In Silico* Analysis

Drug-likeness rules, structural alerts (Brenk and PAINS), bioavailability radar, and BOILED-Egg models predicted using the SwissADME platform under default settings.^22^ Acute oral toxicity (LD_50_ in rats), GHS classification, and organ-specific toxicity end points were predicted using the ProTox-3.0 platform.^23^

### 2.10 Statistical Analysis

All experiments were conducted as three independent biological replicates with technical triplicates. EC_50_ and CC_50_ values, calculated by nonlinear regression analysis using GraphPad Prism 8.0 software, are reported with 95% confidence intervals.^24^

## 3 Results and Discussion

### 3.1 Phytochemical Investigation of *C. Mildbraedii*

The hydroethanolic extracts obtained from the stem bark and roots of *C. mildbraedii* were subjected to successive chromatographic fractionation on silica gel, resulting in the isolation of 17 secondary metabolites (Figure 1). The chemical diversity observed included triterpenoids, phenolic derivatives, nitrogen-containing compounds, and oxygenated fatty acids, reflecting the metabolic richness of this species.

**Figure 1:**
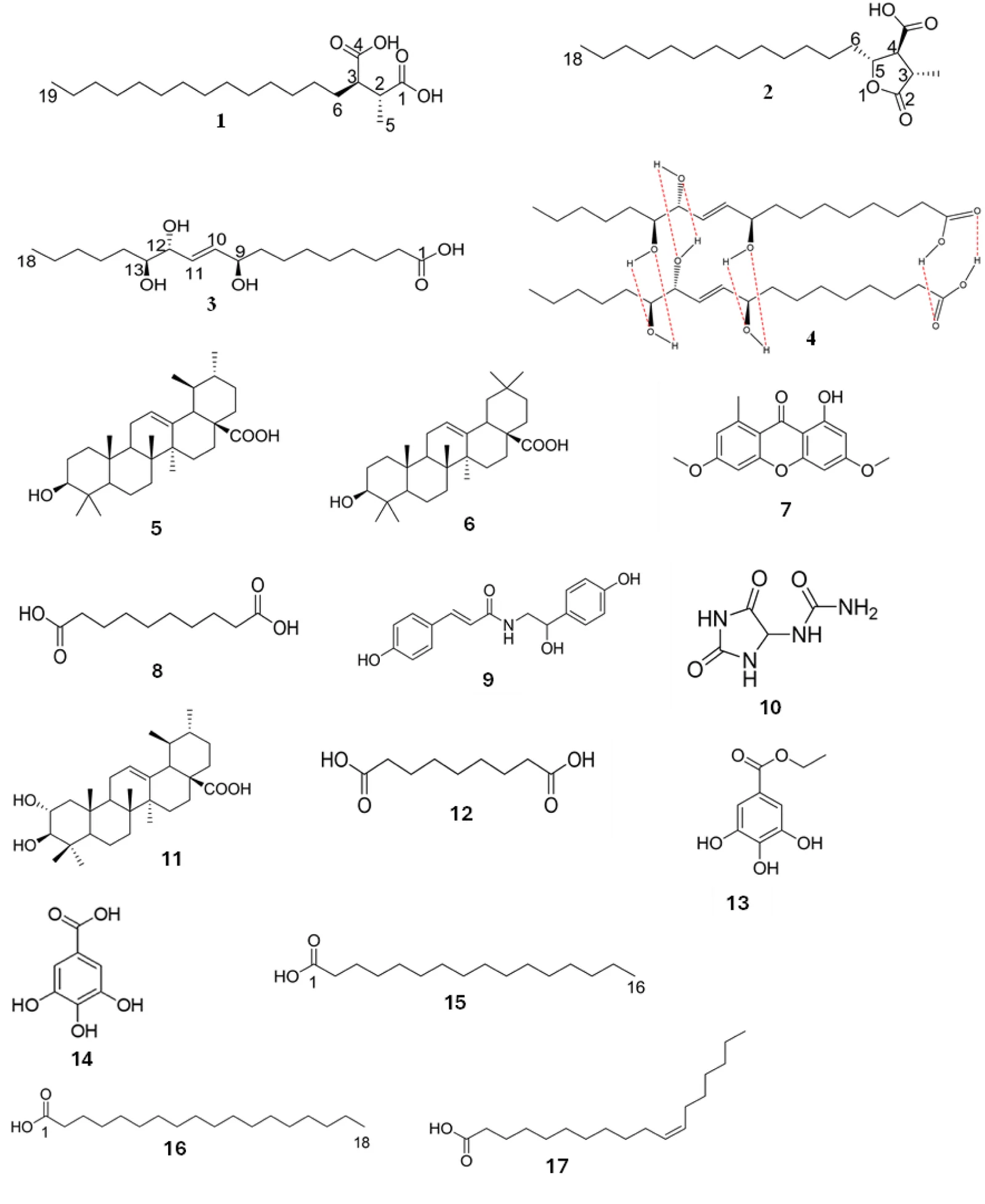
Chemical structures of compounds **1–17**.

From the stem bark extract, eight compounds were isolated and characterized. These included the previously unreported dicarboxylic acid celtic acid (**1**) and the previously unreported paraconic acid (**2**), together with six known constituents: a mixture of ursolic acid (**5**) and oleanolic acid (**6**), lichexanthone (**7**), azelaic acid (**8**), *N-p-trans-*coumaroyloctopamine (**9**), and allantoin (**10**).

The root extract afforded nine compounds, including (9*R*,10*E*,12*R*,13*S*)-9,12,13-trihydroxy-10-octadecanoic acid (**3**), herein reported for the first time as a natural product, and its previously unreported dimer (**4**). Additional metabolites identified from the roots were corosolic acid (**11**), sebacic acid (**12**), ethyl gallate (**13**), gallic acid (**14**), and a mixture of palmitic acid (**15**), cis-vaccenic acid (**16**), and stearic acid (**17**).

The chemical structures of compounds **1**–**17** are presented in Figure 1. The HR-ESI-MS and complete 1D and 2D NMR spectroscopic data for these compounds are provided in the Supporting Information (Figures S1–S59).

### 3.2 Structural Characterization of Previously Unreported Compounds

The structures of compounds **1**–**4** were elucidated through comprehensive spectroscopic analysis, including HR-ESI-MS and extensive one- and two-dimensional NMR experiments. The complete spectroscopic data sets are provided in the Supporting Information (Figures S1–S59, Tables 1 and 2), while the key correlations supporting structural assignments are summarized in Figure 2.

**Figure 2:**
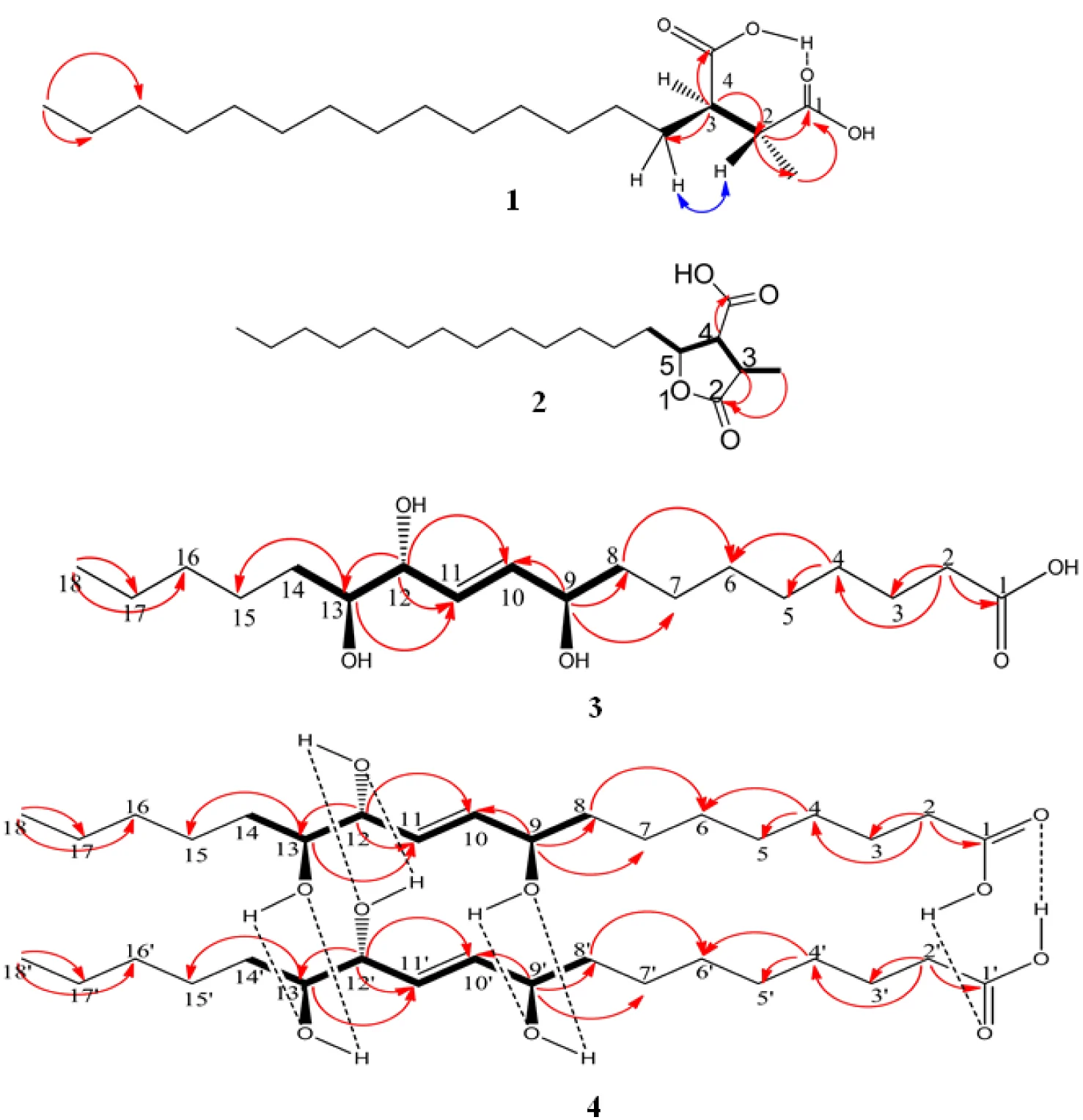
Key COSY (black), HMBC (red), and NOESY (blue dashed) correlations of compounds **1**–**4**.

Compound **1** was obtained as a white crystalline solid. [α]_D_:^25^ +4.7 (c 0.15, (DCM/MeOH 50:50), melting point: 124–126 °C. It reacts positively to the NaHCO_3_ test characteristics of carboxylic acids. Consistent with two degrees of unsaturation, its molecular formula was established as C_19_H_36_O_4_ based on HR-ESI-MS (Figures S1) analysis, which showed the adduct ion peak at *m*/*z* 327.2542 [M – H]^−^ (calcd for C_19_H_35_O_4_^–^ 327.2535). The IR spectrum (Figures S2) suggested the presence of two carboxylic acid groups at υ_O–H_ = 3329 cm^–1^ (free), and υ_O–H_ = 3209 cm^–1^ (bonded) also at υ_C=O_ = 1690 cm^–1^. The 1D and 2 D NMR spectra as ^1^H and ^13^C (Table 1 and Figure S3 and **S4**, respectively), HSQC (Figure S5), COSY (Figure S6), HMBC (Figure S7) and NOESY (Figure S8) revealed signals and correlations characteristic of a substituted succinic acid derivative bearing a aliphatic long chain and methyl group.

Indeed, key COSY correlations (^3^ *J*) confirmed the connectivity between the methine protons H-2 (δ_H_ 2.52, H, m), H-3 (δ_H_ 2.51, H, m) at C-2 (δ_C_ 42.2) and C-3 (δ_C_ 48.3) and adjacent methyl (H-5: δ_H_ 1.10, 3H, d, *J* = 6.3 Hz) and methylene (H-6a/b: δ_H_ 1.54 and 1.39, 2H, m) groups, respectively. HMBC correlations established the positions of the two carboxylic acid carbons and confirmed the substitution pattern. Crucial long-range correlations between H-3 and C-1, C-2, C-4, C-5; H-2 and C-1, C-3, C-4, C-5; H-5 and C-2, C-3, C-1 and also H-19 and C-17, C-18 supported the succinic acid core structure. Additionally, diagnostic NOESY correlations between H-2 and the diastereotopic protons H-6a/b at C-6 (δ_C_ 30.7), as well as between H-3 and the methyl group H-5 at C-5 (δ_C_ 15.1), supported the relative configuration at C-2 and C-3 which may be 2R,3R. The principal COSY, HMBC, and NOESY correlations are depicted in Figure 2.

Based on these spectroscopic data and considering the relative stereochemical relationship between C-2 and C-3, compound **1** was identified as (2*R*,3*R*)-2-methyl-3-tetradecylsuccinic acid, herein named celtic acid, and represents a previously unreported natural product.

Compound **2** was obtained as a white powder. Melting point: 132–134 °C. Test for carboxylic acids is positive. Its HR-ESI-MS (Figure S9) displays the adduct ion peak at *m*/*z* 327.2519 [M + H]^+^ (calcd for C_19_H_35_O_4_^+^ 327.2535) implying the molecular formula C_19_H_34_O_4_ with 3 degrees of unsaturation. Its IR spectrum (Figure S10) showed bands for carboxylic acid group at about υ_O–H_ = 3060 cm^–1^ υ_C=O_ = 1702 cm^–1^, for cyclic ester group at υ_C=O_ = 1737 cm^–1^, and for CH_2_/CH_3_ groups at 2954 and 2849 cm^–1^ respectively. The NMR spectrum ^1^H (Table 1 and Figure S11) combined with the HSQC technique (Figure S12), displayed resonances of: methylene groups at δ_H_ 1.23 (22H, brs) and at δ_H_ 1.3/1.54 (2H, m, H-7*a*/7b); two methyl signals at δ_H_ 1.30 (3H, d, *J* = 7.2 Hz, H-6) and at δ_H_ 0.86 (3H, t, *J* = 6.9 Hz, H-19); and also three methine protons at δ_H_ 4.68 (1H, td, *J* = 9.2, 8.9, 3.6 Hz, H-5); 3.20 (1H, dd, *J* = 9.7, 8.2 Hz, H-4) and δ_H_ 3.02 (1H, dq, *J* = 9.9, 7.2 Hz, H-3). Its ^13^C NMR (Table 1 and Figure S13) sorted by the DEPT technique (Figure S14) are suggestive of γ-butyrolactone moiety and carboxylic acid with carbonyls at δ_C_ 177.4 (C-2) and 174.5 (−COOH) respectively and belonging to a paraconic acid skeleton.^25^ The COSY (Figure S15) and the HMBC (Figure S16) spectra of compound **2** allowed us to connect all the atoms to each (Figure 2). Furthermore, Karplus law permitted us to establish the relative configuration of the three asymmetric carbons C-3, C-4 and C-5, by exploiting their coupling constants. Indeed, on the one hand the coupling between protons H-3 and H-4 with the value of 9.8 Hz, corresponds to a *trans* position between those protons according to Karplus. On the other hand, the coupling between proton H-4 and H-5 with the value of 8.2 Hz, also led to a *transoid* position between the two protons. All of this leads us to believe that the three chiral carbons of compound 2 may have the following configuration, either *3R,4R,5S* or *3S,4S,5R*. The synthetic enantiomer (*3S,4S,5R*) is known as rocellaric acid and all the literature consulted for this compound has displayed the same value of chemical shifts for carbons C-3 (δ_C_ 39.8), C-4 (δ_C_ 53.8) and C-5 (δ_C_ 79.4) in CDCl_3_); for the diastereotropic protons H-7 (δ_H_ 1.65–1.85, m, 2H) in CDCl_3_; and also the same melting point is 98–100 °C;^25–27^ while compound **2** has, in CDCl_3_, roughly the same chemical shifts of carbons and protons except for C-3 (δ_C_ 36.4), C-4 (δ_C_ 51.6), C-5 (δ_C_ 77.2) and H-7 (δ_H_ 1.37–1.54, m, 2H), and also melting point which is 132–134 °C. From the above and so while waiting for the alpha D result, the relative configuration in **2** was proposed as 3*R*,4*R*,5*S*.

Based on all of the above and considering the relative stereochemical relationship between C-3, C-4 and C-5, compound **2** with the trivial name milbraedic acid was identified as (3*R*,4*R*,5*S*)-4-carboxy-3-methyl-5-tridecyl-4,5-dihydro-2­(3H)-furanone being isolated from natural product and described for the first time. Synthetically, paraconic acids are generally derived from the succinic acid derivatives.^28^ So, we think that compound **2** could come from an internal cyclization of compound **1**, the mechanism remains unknown.

Compound **3** was isolated as a white solid. [α]_D_:^25^ −2.8 (c 0.30, (DCM/MeOH 1:1), melting point: 106–108 °C. It reacts positively to NaHCO_3_ for carboxylic acids test. Its HR-ESI-MS (Figure S17) showed the sodium adduct at *m*/*z* 353.2314 [M + Na]^+^ (calcd for C_18_H_34_O_5_Na^+^ 353.2304) corresponding to the molecular formula C_18_H_34_O_5_, indicating two degrees of unsaturation. The IR spectrum (Figure S18) of **3** showed characteristic absorption bands for carboxylic acid group at υ_O–H_ = 3200 cm^–1^ and υ_C=O_ = 1697 cm^–1^, and for hydroxyl group at υ_O–H_ = 3278 cm^–1^ and for olefinic groups υ_O–H_ = 1468 cm^–1^. The ^1^H NMR (Table 2 and Figure S19) and HSQC (Figure S20) spectra displayed two olefinic protons at δ_H_ 6.58 (1H, ddd, *J* = 15.8, 10.0, 5.9 Hz, H-11) and δ_H_ 6.42 (1H, ddd, *J* = 15.7, 10.0, 6.2 Hz, H-10) with a coupling constant of 15.8 Hz consistent of a *trans* configuration of the double bond, as well as three oxymethine signals at δ_H_ 4.69 (1H, m, H-12), δ_H_ 4.60 (1H, m, H-9) and δ_H_ 4.15 (1H, m, H-13) indicative of a trihydroxylated fatty acid framework. The ^13^C NMR (Table 2 and Figure S21) spectrum confirmed the presence of one carboxylic acid carbon [δ_C_ 177.2 (C-1)], two olefinic carbons [δ_C_ 137.6 (C-10) and δ_C_ 132.1 (C-11)], three oxygenated methines [δ_C_ 77.5 (C-12), δ_C_ 76.5 (C-13) and δ_C_ 73.2 (C-9)], multiple aliphatic methylenes (δ_C_ 24.3–39.8), and a terminal methyl [δ_C_ 15.5 (C-18)]. COSY (Figure S22) and HMBC (Figure S23) correlations defined the continuous carbon chain and confirmed connectivity between the olefinic and oxymethine protons. HMBC cross-peaks of H-10 and H-11 with neighboring carbons established the position of the double bond within the carbon chain. The positions of the functional groups in this compound can be confirmed by the presence of two important fragment ions observed on its HR-ESI mass spectrum (Figure S17) at *m*/*z* 155 [(M – 2H) – 173]^+^ and 173 (M – 157)^+^ (Scheme S1). The combined spectroscopic evidence with the literature data supported the structure of (9*R*,10*E*,12*R*,13*S*)-9,12,13-trihydroxy-10-octadecanoic acid. Indeed, by comparing the optical rotation value of compound **3** with those of its stereoisomers synthesized by Shirahata et al. (2006), only the stereoisomer, with configurations 9*R*, 12*R* and 13*S*, have a close alpha D [[α]_D_:^30^ −5.3 (c 0.15, (MeOH)], and this allowed assignment of the absolute configuration.^29^ Although previously described as a synthetic compound, this study represents the first report of its occurrence as a natural product.

Compound **4** was isolated as a white powder. [α]_D_:^25^ −10.22 (c 0.20, (MeOH), melting point: 116–118 °C. Its molecular formula C_36_H_68_O_10_ was deduced from its HR-ESI-MS (Figure S24), which showed the sodium adduct [M + Na]^+^ at *m*/*z* 683.4777 (calcd for C_36_H_68_O_10_Na^+^: 683.4710). Compared to compound **3**, Its molecular formula and its mass suggest its dimeric structure. Furthermore, the qualitative analysis by LC-MS (Figure S25) shows a single peak demonstrating the purity of this compound. Its IR spectrum (Figure S26) is quite similar to that of compound **3** (Figure S18), particularly the vibration bands of carbonyl, olefinic and CH_2_/CH_3_ groups which are the same in both compounds (1697, 1468 and 295/2848 cm^–1^ respectively). However, in the vibration region of O–H bands, the absorption bands of compound **4** have a lower wavenumber (υ_O–H_ = 3270 cm^–1^ for alcohols; υ_O–H_ = 3196 cm^–1^ for acids) than that of compound **3** (υ_O–H_ = 3278 cm^–1^; υ_O–H_ = 3201 cm^–1^). This difference means that the O–H bonds in compound **4** are less strong than those of **3**: this type of phenomenon is usually caused by the formation of hydrogen bonds. Likewise, its 1D NMR spectra (^1^H and ^13^C (Table 2 and Figure S27, S28, respectively) and also its 2D spectra; COSY (Figure S29), HSQC (Figure S30) and HMBC (Figure S31), display signals similar to those of compound **3**, except that the signals of compound **4** were duplicated, (Figures S32 and S33). This is consistent with symmetrical association of two monomeric units.

The absence of significant chemical shift deviations relative to compound **3**, together with mass spectrometric evidence, supported the formation of a hydrogen-bonded acid dimer, through the different hydroxyl groups, rather than a covalent dimer. All these spectral data suggested that compound **4** could be formed by dimerization of compound **3**.

This could be demonstrated by the following evidence. Indeed, recorded in deuterated methanol, the spectral profile of the proton of this dimer (**4**) verifies the theory proposed on all the eight isomers synthesized stereoselectively by Shirahata et al. (2006).^29^ Exploiting the Karplus rule, this theory highlights relationship between the absolute configuration of carbons C-9, C-12, and C-13 and the pattern of the ^1^H NMR signals (Figure S33). Based on this theory, since proton H-13 of compound **4** is located in a weaker field (δ_H_ 3.50), this would mean that the OH groups at C-12 and C-13 are antidiol and therefore the configurations could be (12*R* and 13*S*) or (12*S* and 13*R*) respectively. Furthermore, given that signals of protons H-10 and H-11 are very close and confused, the absolute configurations of carbons C-9 and C-12 would be (9*S*,12*S*) or (9*R*,12*R*) respectively. According to this, the absolute configurations of the monomer would be either 9*R*,12*R*,13*S* (**3**) or its enantiomer 9*S*,12*S*,13*R*. Then, the dimer **4** could be a combination of these two monomers as follows: (9*R*,12*R*,13*S*) (**3**)/(9*R*,12*R*,13*S*) (**3**): (**CC1**); (9*S*,12*S*,13*R*)/(9*S*,12*S*,13*R*): (**CC2**) or (9*R*,12*R*,13*S*) (**3**)/9*S*,12*S*,13*R*): (**CC3**).

By comparing the optical rotations of these two synthetic stereoisomers: [9*R*,12*R*,13*S*: [α]­D:^30^ −5.3 (c 0.15, (MeOH)] and [9*S*,12*S*,13*R*: [α]­D:^25^ +6.7 (c 0.14, MeOH)] with that of compounds **3**; [[α]­D:^25^ −2.8 (c 0.30, DCM/MeOH 50:50)] or **4** [[α]_D_:^25^ −10.22 (c 0.20, MeOH)], we believe that compound **3** is exclusively the monomer of the dimer **4** (**CC1**). In fact, the dimer **CC3** which is made up of the two enantiomers, would have a racemic mixture and therefore alpha D of **4** will be null. Also, given the negative sign of the alpha D of **4**, the **CC2** dimer may be have a positive sign. Furthermore, as we will see later, under the same conditions and on the same biological target, compounds **3** and **4** show completely different results.

Therefore, compound **4**, with the trivial name niemeyong acid dimer, was identified as a dimer of (9*R*,10*E*,12*R*,13*S*)-9,12,13-trihydroxy-10-octadecanoic acid (**3)** and described for the first time. The key spectroscopic correlations for compound **4** are also summarized in Figure 2.

Collectively, the spectroscopic analyses unambiguously established the structures of compounds **1**–**4** and expanded the known chemical diversity of the genus *Celtis*.

### 3.3 Exploratory *In Vitro* Screening against *S. Mansoni*

All 17 isolated metabolites (**1**–**17**) were initially evaluated in an exploratory phenotypic screening assay at a fixed concentration of 200 μg/mL against adult worms and newly transformed schistosomula of *S. mansoni*. This concentration was selected to ensure methodological consistency across extracts, fractions, and purified compounds during the preliminary screening phase.

The screening results are summarized in Figure 3. At 200 μg/mL, only three compounds, namely corosolic acid (**11**), trihydroxyoctadecanoic acid (**3**), and gallic acid (**14**), induced measurable mortality in adult worms, whereas the remaining metabolites showed minimal or no detectable effect. None of the evaluated compounds significantly affected newly transformed schistosomula under the same experimental conditions.

**Figure 3:**
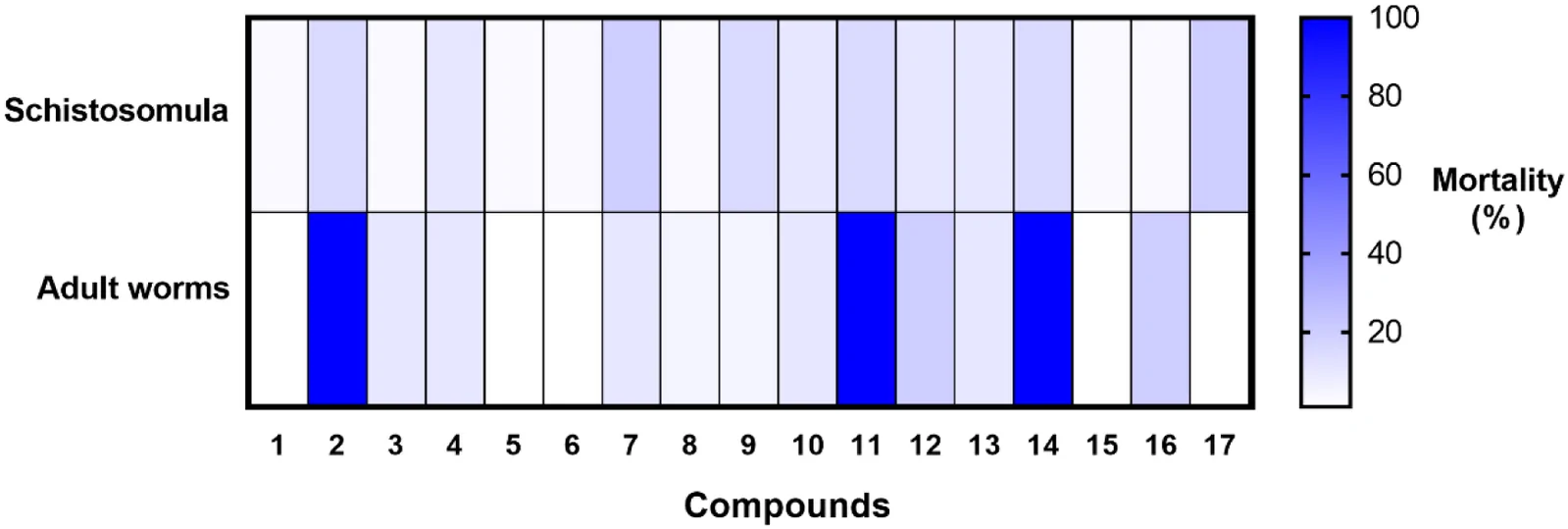
*In vitro* screening of compounds **1**–**17** against adult worms and newly transformed schistosomula (NTS) of *S. mansoni* at 200 μg/mL. Heatmap representation of parasite mortality (%) after 72 h of incubation.

Concentration–response assays were subsequently performed for the active compounds to determine EC_50_ values (Table 3). Corosolic acid (**11**) exhibited the lowest EC_50_ (158.6 μM), followed by compounds **3** (302.6 μM) and **14** (764.2 μM). Although corosolic acid (**11**) exhibited only modest antischistosomal activity, indicating that it should not be considered a highly potent lead compound, its potency is comparable to that reported for several natural and semisynthetic compounds previously evaluated *in vitro* against *S. mansoni*. These include phytol (∼170 μM),^30^ hesperidin (∼165 μM),^31^ miltefosine (100–200 μM),^32^ cyproterone acetate (∼240 μM),^33^ and artemisinin derivatives such as artemether (∼335–1000 μM)^34^ and artesunate (∼100–780 μM).^35^ Within the triterpenoid class, compounds such as friedelan-3-one, β-sitosterol/stigmasterol, and dichapetalin derivatives including dichapetalin M and dichapetalin A have also been reported to exhibit activity against adult schistosomes at concentrations above 250 μM.^36^ Other natural metabolites, including arachidonic acid (∼5000 μM)^37^ and epiisopiloturine (>600 μM),^38^ have likewise been described as active only at relatively high micromolar concentrations. Collectively, these comparisons indicate that measurable activity in the high micromolar range has been reported for several structurally diverse natural products evaluated against *S. mansoni*. Although such potency remains modest compared with the most active antischistosomal natural products, these compounds may still represent useful starting points for future scaffold optimization and structure–activity relationship studies.

**Table 3:** *In Vitro* Antischistosomal Activity and Selectivity Profile of Active Compounds

Compounds	EC_50_ (μM)^a^	CC_50_ (μM)^b^ ^,+^	CC_50_ (μM)^b^ ^,++^	SI^c^	LD_50_ (μM)^d^
**3**	302.6 [283.7–321.5]	>2000	>2000	6.6	>2000
**11**	158.6 [144.3–172.9]	>2000	>2000	12.6	>2000
**14**	764.2 [739.6–788.8]	>2000	>2000	2.61	>2000

a EC_50_ values against adult *S. mansoni* worms after 72 h incubation. Values in brackets represent 95% confidence intervals obtained by nonlinear regression analysis.

b CC_50_ values determined in Vero (^**+**^) and HaCaT (^**++**^) cells after 72 h incubation.

c Selectivity index calculated as CC_50_/EC_50_.

d LD_50_ values determined in *C. elegans* under the tested conditions.

The lack of activity against newly transformed schistosomula aligns with the well-established stage-dependent susceptibility of *S. mansoni*.^12,15^ Biological and biochemical differences between larval and adult worms, including variations in tegument structure, metabolic activity, membrane permeability, and target expression, are known to influence drug responsiveness.^39,40^ These findings reinforce the importance of evaluating compounds across multiple developmental stages in antischistosomal discovery efforts.

Additional parameters, including cytotoxicity in mammalian cell lines, selectivity indices, and lethality in *C. elegans*, are summarized in Table 3 and discussed in Section 3.5.

### 3.4 Structure–Activity Considerations

Although only three compounds exhibited measurable activity against adult *S. mansoni*, the structural diversity among these metabolites allows preliminary structure–activity considerations. The active compounds belong to distinct chemical classes, including a pentacyclic triterpenoid (**11**), a polyhydroxylated fatty acid (**3**), and a simple phenolic acid (**14**), suggesting that no single structural motif exclusively accounts for the observed activity.

Previous studies indicate that gallic acid (**14**) exhibits activity against both protozoan and helminth parasites. It has shown activity against intraerythrocytic forms of *Plasmodium falciparum* at approximately 40 μM,^41^ against *Trypanosoma brucei* at approximately 15 μM,^42^ and against *Leishmania major* at approximately 100 μM.^43^ Gallic acid has also been reported to exhibit larvicidal activity against infective larvae of *Hemonchus contortus*, with an LC_50_ of approximately 11,800 μM for the commercial standard.^44^ In contrast, to our knowledge, specific antiparasitic activity has not previously been demonstrated for compounds **11** and **3** as isolated compounds. These observations suggest that gallic acid possesses broader antiparasitic activity, whereas the effects observed here for compounds **11** and **3** may represent previously unreported antiparasitic properties.

Corosolic acid (**11**), the most active compound identified in this study, is a pentacyclic triterpenoid characterized by a rigid hydrophobic scaffold bearing hydroxyl and carboxylic acid functionalities. Triterpenoids have previously been reported to display *in vitro* activity against *Schistosoma* species, often attributed to interactions with the parasite tegument or membrane structures.^45,46^ The amphiphilic nature of corosolic acid, combining a lipophilic backbone with polar substituents, may facilitate interaction with lipid-rich surfaces of adult worms. The higher activity of compound **11** relative to the other tested metabolites may therefore be associated with its balanced hydrophobic and hydrogen-bonding properties.

Compound **3**, a trihydroxylated long-chain fatty acid, displayed weaker activity compared to compound **11**. Oxygenated fatty acids have been described as modulators of membrane integrity and lipid organization in various biological systems.^47^ The presence of three hydroxyl groups increases polarity relative to nonoxygenated fatty acids, potentially influencing membrane permeability and molecular orientation at biological interfaces. However, the flexible aliphatic chain and high polarity may limit effective interaction with specific parasite targets, which could explain the modest potency observed. Furthermore, no perceptible activity is observed from the dimer of **3**, probably because, as mentioned above, this dimer (**4**) does not have free hydroxyl groups which are involved in hydrogen bonding.

Gallic acid (**14**), the least potent of the active metabolites, is a small, highly polar phenolic acid bearing three hydroxyl groups. Interestingly, its alkylated derivative ethyl gallate (**13**) did not display measurable activity under the same conditions. This observation suggests that free phenolic hydroxyl groups may contribute to biological effect, possibly through redox-related mechanisms or hydrogen-bonding interactions. At the same time, the high polarity and low molecular complexity of gallic acid may restrict its ability to penetrate or accumulate within parasite tissues, which may account for its comparatively weak activity.

Taken together, these observations suggest that amphiphilicity and molecular size may influence *in vitro* activity against adult *S. mansoni*. Compounds combining hydrophobic frameworks with polar functional groups, such as corosolic acid, may achieve more favorable interactions with parasite membranes or surface structures. However, these structure–activity considerations do not exclude the possibility that the observed effects result from interactions with other, unrelated molecular targets in the parasite. Nevertheless, given the high micromolar activity range and the limited number of active metabolites, these interpretations remain preliminary. Further chemical modification and targeted mechanistic studies would be required to define the structural features associated with improved potency.

### 3.5 Cytotoxicity and Selectivity

To evaluate whether the observed antischistosomal effects were associated with nonspecific cytotoxicity, the active compounds were tested in mammalian cell lines and in *C. elegans*. The corresponding CC_50_ values in mammalian cells, LD_50_ values in *C. elegans*, and calculated selectivity indices are summarized in Table 3.

None of the evaluated compounds exhibited detectable cytotoxicity in mammalian cells at concentrations up to 2000 μM. Similarly, no lethality was observed in *C. elegans* within the tested concentration range. These findings indicate that the measurable activity detected against adult *S. mansoni* worms is unlikely to result from general cytotoxic effects.

The calculated selectivity indices remained modest due to the high micromolar EC_50_ values observed in the parasite assays. Nevertheless, the absence of cytotoxicity at concentrations exceeding those required to induce schistosomicidal effects suggests a preliminary *in vitro* safety margin. Comparable safety profiles have been reported for structurally diverse natural products evaluated under similar experimental conditions,^48,49^ reinforcing the reliability of this screening platform for distinguishing parasite-specific effects from general cytotoxicity.

The lack of toxicity in *C. elegans*, a free-living nematode model frequently used for preliminary safety assessment, further supports the limited general toxicity of these metabolites. Although differences between trematodes and nematodes preclude direct extrapolation, the absence of lethality in this model provides additional evidence that the observed effects are not associated with broad-spectrum invertebrate toxicity.^50,51^

Taken together, these results indicate that the detected schistosomicidal activity occurs in the absence of significant cytotoxic or general nematocidal effects under the tested conditions. While the overall antiparasitic potency remains limited, the safety profile observed *in vitro* suggests that future efforts would primarily require structural optimization to enhance activity rather than mitigation of toxicity.

### 3.6 *In Silico* Physicochemical and Pharmacokinetic Evaluation

To further characterize the three metabolites that exhibited measurable activity against adult *S. mansoni*, an *in silico* physicochemical and pharmacokinetic evaluation was conducted using the SwissADME platform. Drug-likeness rule compliance and structural alert analyses are summarized in Figure 4, while predicted pharmacokinetic properties are shown in Figure 5.

**Figure 4:**
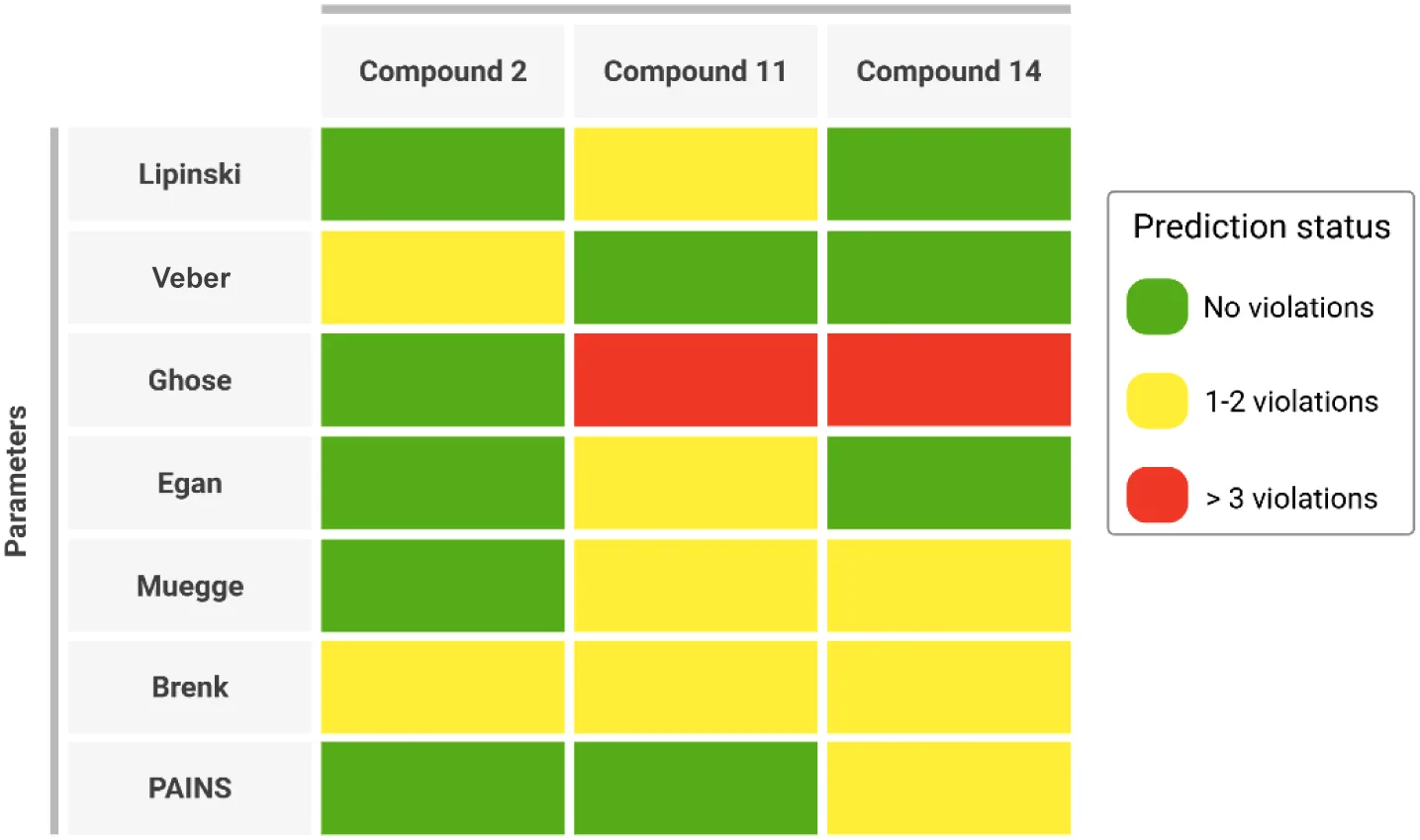
*In silico* drug-likeness rule compliance and structural alert assessment of compounds **2**, **11**, and **14**. Heatmap summarizing compliance with Lipinski, Veber, Ghose, Egan, and Muegge drug-likeness rules, together with Brenk and PAINS structural alert analyses generated using the SwissADME platform. Green indicates no violations; yellow indicates one to two violations; red indicates more than three violations. Detailed physicochemical parameters are provided in the Supporting Information.

**Figure 5:**
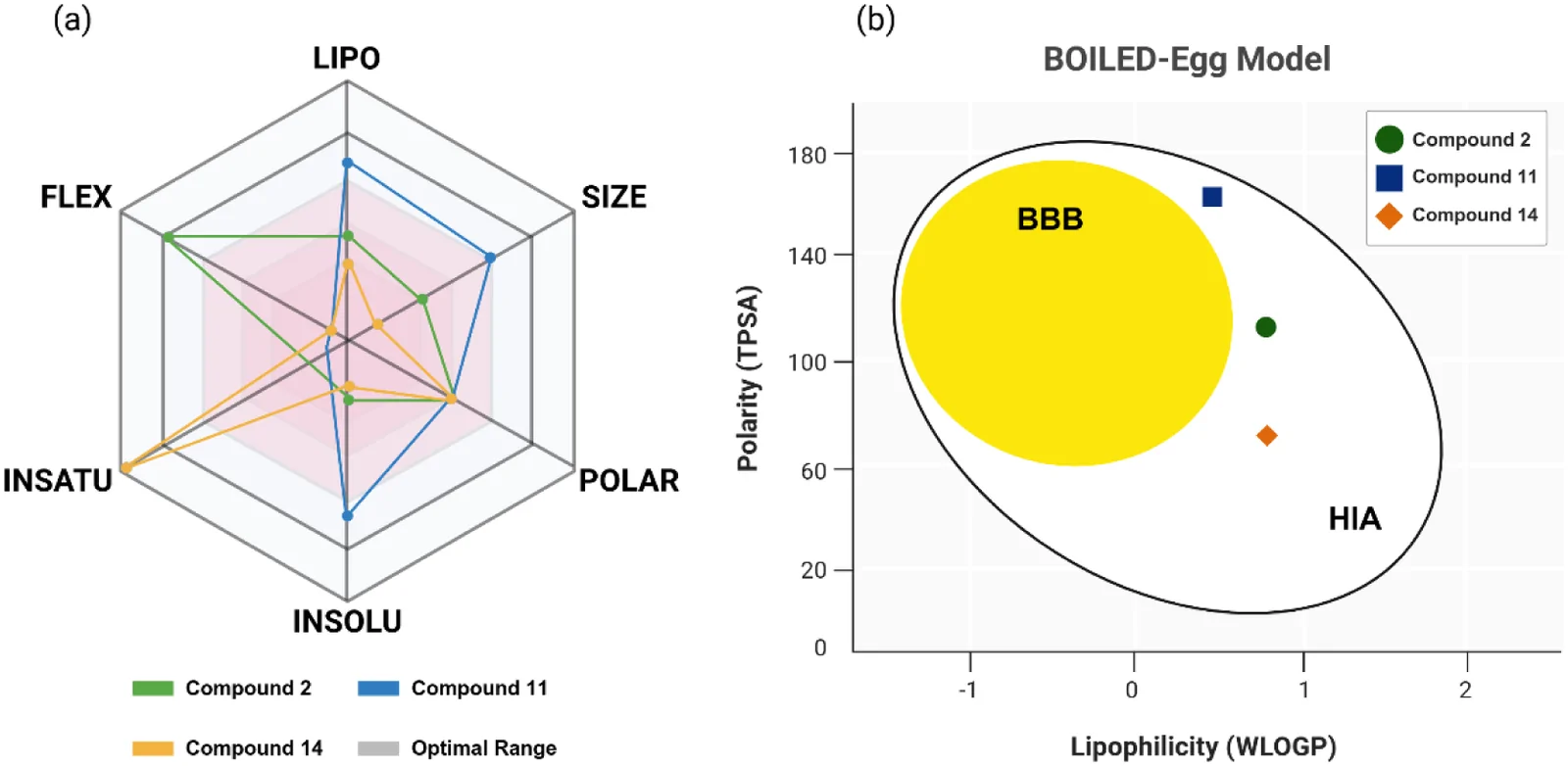
Predicted pharmacokinetic properties of compounds **2**, **11**, and **14**. (a) Bioavailability radar summarizing key physicochemical parameters influencing oral bioavailability, including lipophilicity (LIPO), size (SIZE), polarity (POLAR), solubility (INSOLU), flexibility (FLEX), and saturation (INSATU). The shaded region represents the optimal range for oral drug-likeness. (b) BOILED-Egg model predicting passive gastrointestinal absorption (HIA) and blood–brain barrier (BBB) permeability based on lipophilicity (WLOGP) and polarity (TPSA). Colored markers correspond to compounds **2**, **11**, and **14**. Predictions were generated using the SwissADME platform.

Compliance with classical drug-likeness filters revealed distinct physicochemical profiles among the compounds (Figure 4). Compound **3** largely satisfied Lipinski, Ghose, and Egan criteria, with minor violations associated with molecular flexibility. Compound **11** fulfilled Lipinski and Veber parameters but exceeded certain lipophilicity and molecular size thresholds in other filters, consistent with its pentacyclic triterpenoid structure. Compound **14** complied with most filters but showed deviations related to molecular size and polarity, reflecting its low molecular weight and highly polar phenolic scaffold. Brenk and PAINS analyses indicated limited structural alerts across the series, with no extensive pan-assay interference concerns.

The bioavailability radar (Figure 5a) provides a comparative visualization of lipophilicity, size, polarity, solubility, flexibility, and saturation. Compound **11** displayed elevated lipophilicity and molecular size, remaining partially outside the optimal oral bioavailability region, whereas compound **14** exhibited high polarity and low lipophilicity. Compound **3** showed intermediate characteristics, combining moderate lipophilicity with increased conformational flexibility. These differences reflect the structural diversity of the evaluated metabolites.

The BOILED-Egg model (Figure 5b), which predicts passive gastrointestinal absorption and blood–brain barrier permeability based on lipophilicity and topological polar surface area, positioned all three compounds within the region associated with a high probability of gastrointestinal absorption. None of the compounds were predicted to permeate the blood–brain barrier. This profile is consistent with their intended antiparasitic application, where central nervous system exposure is not required.^52,53^

Overall, the *in silico* analyses highlight pronounced physicochemical differences among compounds **3**, **11**, and **14** while indicating generally acceptable oral absorption potential. Although these parameters do not directly correlate with the observed *in vitro* potency, they provide a framework for understanding molecular properties that may influence distribution and bioavailability in future optimization efforts.

### 3.7 *In Silico* Toxicity Prediction

To complement the experimental cytotoxicity evaluation, computational toxicity predictions were performed using the ProTox-3.0 platform. The predicted acute oral LD_50_ values in rats and corresponding Globally Harmonized System (GHS) classifications are presented in Figure 6a.

**Figure 6:**
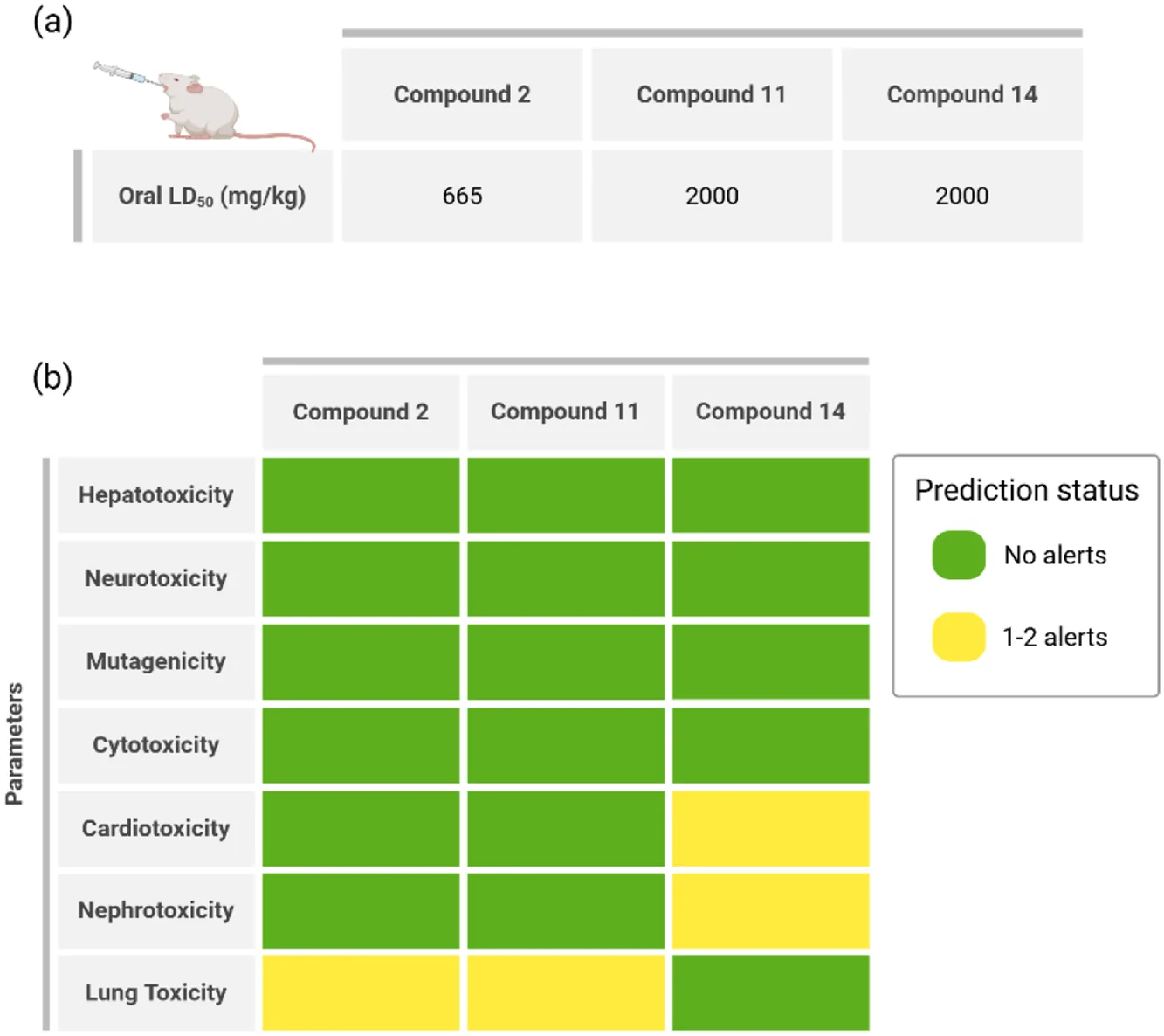
*In silico* acute toxicity prediction of compounds 2, 11, and 14. (a) Predicted acute oral LD50 values in rats (mg/kg) and corresponding GHS toxicity classification. (b) Heatmap summarizing predicted toxicity end points, including hepatotoxicity, neurotoxicity, mutagenicity, cytotoxicity, cardiotoxicity, nephrotoxicity, and lung toxicity. Green indicates no predicted alerts; yellow indicates one to two predicted alerts. Detailed probability scores are provided in the Supporting Information. Predictions were generated using the ProTox-3.0 platform.

Compounds **11** and **14** were predicted to have an acute oral LD_50_ of 2000 mg/kg in rats, whereas compound **3** exhibited a predicted value of 665 mg/kg. All three compounds were classified within GHS toxicity class 4, indicating low acute oral toxicity according to established regulatory criteria. These predictions suggest a favorable acute toxicity profile at the systemic level.

Predicted toxicity end points are summarized in Figure 6b. No alerts were identified for hepatotoxicity, neurotoxicity, mutagenicity, or general cytotoxicity across the three compounds. Moderate alerts were predicted for cardiotoxicity and nephrotoxicity in compound **14**, and for lung toxicity in compounds **3** and **11**. These alerts were associated with moderate probability values and should be interpreted cautiously, as *in silico* models may overestimate organ-specific risks, particularly for structurally diverse natural products.

The absence of predicted mutagenicity and broad cytotoxic liability is consistent with the experimental findings described in Section 3.5, where no cytotoxic effects were observed in mammalian cell lines or in *C. elegans* at the highest tested concentrations. Although computational predictions do not substitute for experimental toxicological assessment,^54^ the combined *in vitro* and *in silico* results indicate that the primary limitation of these metabolites lies in their moderate antiparasitic potency rather than predicted systemic toxicity.

### 3.8 Chemotaxonomic Significance of the Isolated Compounds

From the stem barks and roots of *C. mildbraedii* 17 compounds were isolated of which 3 dicarboxylic acids, 4 monocarboxylic acids, 1 dimer of monocarboxylic acid, 1 paraconic acid, 3 triterpenoids, 1 xanthone, 1 phenolic amide, 2 phenolic acids and 1 alkaloid. Despite no chemical and pharmacological works have been carried out on the stem barks and roots of *C. mildbraedii*, the chemotaxomic study performed on the obtained molecules suggests that this plant does, indeed, belong to the *Celtis* genus where numerous chemical works have been reported. Therefore, the presence of celtic acid (**1**) and mildbraedic acid (**2**) was not a surprise since succinic acid was previously isolated from *Celtis tessmannii* Rendle^55^ while dicarboxylic acid derivatives like azelaic acid (**12**) and sebacic acid (**8**) were isolated in the roots of *C. adolphi friderici*.^56^ Also, the presence of fatty acid derivatives like (9*R*,10*E*,12*R*,13*S*)-9,12,13-trihydroxy-10-octadecanoic acid (**3**) has been described from *C. tessmannii*,^57^ *C. adolphi-friderici* ^56^ and *C. Africana*.^57^

In addition, oleanolic acid (**6**) and ursolic acid (**5**) were isolated from *C. tessmannii* ^55^ and *C. philippinensis*.^58^ The phenolic amide *N*-*p*-trans-coumaroyloctopamine (**9**) were isolated from *C. adolphi-friderici*,^56^ *C. africana*,^57^ *C. occidentalis*,^59^ *C. sinensis*,^60^ *C. tessmanii* ^55^ and *C. zenkeri*.^61^ The imidazole alkaloid allantoin (**10**) was isolated from *C. adolphi-friderici*.^56^ Benzoic acids were also isolated from *C. australis* ^62^ and *C. adolphi-friderici*.^56^

Moreover, this work stands as the first report of the isolation of lichexanthone (**7**) and corosolic acid (**11**) in the *Celtis* genus. The discovery of these compounds expands the known phytochemical profile of the *Celtis* genus, providing a strong basis for future chemotaxonomic research.

## 4 Conclusion

This study reports the phytochemical investigation of *C. mildbraedii*, leading to the isolation and structural characterization of 17 metabolites, including previously undescribed compounds. The integration of phytochemical analysis with exploratory *in vitro* screening and computational evaluation provides the first systematic assessment of this species in the context of antischistosomal research.

Phenotypic screening against adult *S. mansoni* identified three metabolites with measurable activity at high micromolar concentrations, with corosolic acid (**11**) exhibiting the lowest EC_50_ value. Although the overall antiparasitic potency observed in this study was modest and does not support the identified compounds as highly potent lead candidates, the activity range is comparable to that reported for several structurally diverse natural products previously evaluated against *S. mansoni*. The complete lack of activity against newly transformed schistosomula further highlights the stage-dependent susceptibility of this parasite.

Importantly, the active compounds did not exhibit cytotoxicity in mammalian cell lines or lethality in *C. elegans* under the tested conditions. *In silico* analyses further supported a generally favorable acute toxicity profile and predicted gastrointestinal absorption, despite structural differences among the evaluated metabolites. These findings suggest that the primary limitation of the identified scaffolds lies in their moderate potency rather than overt toxicity concerns.

While none of the isolated metabolites fulfill criteria for advanced development in their current form, the structural diversity uncovered in *C. mildbraedii* expands the chemical space of natural products evaluated against *S. mansoni*. Nevertheless, these findings reinforce that phenotypic screening of chemically diverse natural products remains a valuable strategy for identifying novel antiparasitic scaffolds, even when the initial potency is modest. The combined phytochemical and biological data provide a foundation for future structure–activity relationship studies and structure-based optimization, thereby contributing to ongoing efforts aimed at diversifying the antiparasitic discovery pipeline.

## Supplementary Material



## Notes

Table S1. Molecular weights, purity levels, and molar concentrations corresponding to the fixed screening concentration of 200 μg/mL for the isolated metabolites. Figure S1: HR-ESI-MS spectrum of compound **1.** Figure S2: IR spectrum of compound **1.** Figure S3: ^1^H NMR spectrum {500 MHz, CDCl_3_/CD_3_OD (1:1)} of compound **1.** Figure S4: HSQC spectrum of compound **1.** Figure S5: ^13^C NMR spectrum {125 MHz, CDCl_3_/CD_3_OD (1:1)} of compound **1.** Figure S6: COSY spectrum of compound **1.** Figure S7: HMBC spectrum of compound **1.** Figure S8: NOESY spectrum of compound **1.** Figure S9: ESI mass spectrum of compound **2.** Figure S10: IR spectrum of compound **2.** Figure S11: ^1^H NMR spectrum (CDCl_3_, 500 MHz) of compound **2.** Figure S12: HSQC spectrum of compound **2.** Figure S13: ^13^C NMR spectrum (CDCl_3_, 125 MHz) of compound **2.** Figure S14: DEPT spectrum in CDCl_3_ of compound **2.** Figure S15: COSY spectrum of compound **2.** Figure S16: HMBC spectrum of compound **2.** Figure S17: HR-ESI-MS spectrum of compound **3.** Figure S18: IR spectrum of compound **3.** Figure S19: ^1^H NMR (500 MHz, C_5_D_5_N) spectrum of compound **3.** Figure S20: HSQC spectrum of compound **3.** Figure S21: ^13^C NMR (125 MHz, C_5_D_5_N) spectrum of compound **3.** Figure S22: COSY spectrum of compound **3.** Figure S23: HMBC spectrum of compound **3.** Scheme 1: Mass fragmentation pattern of **3.** Figure S24: HR-ESI-MS spectrum of compound **4.** Figure S25: LC-MS profile of compound **4.** Figure S26: IR spectrum of compound **4.** Figure S27: ^1^H NMR (500 MHz, CD_3_OD) spectrum of compound **4.** Figure S28: ^13^C NMR (125 MHz, CD_3_OD) spectrum of compound **4.** Figure S29: COSY spectrum of compound **4.** Figure S30: HSQC spectrum of compound **4.** Figure S31: HMBC spectrum of compound **4.** Figure S32: Compared ^13^C NMR (125 MHz, CD_3_OD) spectrum of compounds **4** and **3** {125 MHz, CDCl_3_/CD_3_OD (1:1)}. Figure S33: Extended ^13^C NMR (125 MHz, CD_3_OD) spectrum of compound **4.** Figure S34: ^1^H NMR spectra of stereoisomers of pinellic acid. Figure S35: ^1^H NMR spectrum (Acetone-*d*_6_, 500 MHz) of the mixture of **5** and **6.** Figure S36: ^13^C NMR spectrum (Acetone-*d*_6_, 125 MHz) of the mixture of **5** and **6.** Figure S37: ^1^H NMR spectrum (CDCl_3_, 500 MHz) of **7.** Figure S38: ^13^C NMR spectrum (CDCl_3_, 125 MHz) of **7.** Figure S39: HR-ESI-MS of **8.** Figure S40: ^1^H NMR spectrum (CD_3_OD, 500 MHz) of **8.** Figure S41: ^13^C NMR spectrum (CD_3_OD, 125 MHz) of **8.** Figure S42: ^1^H NMR spectrum (CDCl_3_/CD_3_OD, 600 MHz) of **9.** Figure S43: ^13^C NMR spectrum (CDCl_3_/CD_3_OD, 150 MHz) of **9.** Figure S44: ^1^H NMR spectrum (DMSO-*d*_6_, 500 MHz) of **10.** Figure S45: ^13^C NMR spectrum (DMSO-*d*_6_, 125 MHz) of **10.** Figure S46: ^1^H NMR spectrum of **11** (500 MHz, DMSO-*d*_6_). Figure S47: ^13^C NMR spectrum of **11** (125 MHz, DMSO-*d*_6_). Figure S48: HR-ESI-MS of **12.** Figure S49: ^1^H NMR spectrum (CDCl_3_/CD_3_OD, 600 MHz) of **12.** Figure S50: ^13^C NMR spectrum {150 MHz, CDCl_3_/CD_3_OD (1:1)} of **12.** Figure S51: ^1^H NMR spectrum (DMSO-*d*_6_, 500 MHz) of **13.** Figure S52: ^13^C NMR spectrum (DMSO-*d*_6_, 125 MHz) of **13.** Figure S53: ^1^H NMR spectrum (DMSO-*d*_6_, 500 MHz) of **14.** Figure S54: ^13^C NMR spectrum (DMSO-*d*_6_, 125 MHz) of **14.** Figure S55: GC/EIMS pic 1 of **15.** Figure S56: GC/EIMS pic 2 of **16.** Figure S57: GC/EIMS pic 3 of **17.** Figure S58: ^1^H NMR spectrum (CDCl_3_, 500 MHz) of the mixture of **15**, **16** and **17.** Figure S59: ^13^C NMR spectrum (CDCl_3_, 125 MHz) of the mixture of **15**, **16** and **17** (PDF)
