# Supplementary material for: Phytochemical Investigation of Celtis Mildbraedii Engl and Exploratory In Vitro and In Silico Evaluation against Schistosoma Mansoni
Source: ACS Omega. 2026 Sep 25;11(39):59226–39. doi: 10.1021/acsomega.6c05025 (PMC13647360; doi:10.1021/acsomega.6c05025)
Supplement: Supplementary file 1 [file ao6c05025_si_001.pdf]

Supplementary material

Phytochemical Investigation of *Celtis mildbraedii* Engl and Exploratory *In Vitro* and *In Silico* Evaluation against *Schistosoma mansoni*

Fabiola Tanekou Madaha [a,b], Lucas Fukui-Silva [b], Monique C. Amaro [b], Johane K. Dongmo Jumeta [a], Diderot Noungoue Tchamo [a*], and Josué de Moraes [b,c*]

[a] *Department of Organic Chemistry, Faculty of Science, University of Yaounde I, P.O. Box 812, Yaounde, Cameroon.*

[b] *Research Center on Neglected Diseases, Guarulhos University, Guarulhos, 07023-070, SP, Brazil.*

[c] *Research Center on Neglected Diseases, Scientific and Technological Institute, Brazil University, São Paulo, SP 08230-030, Brazil.*

*J. de Moraes

Email: moraesnpdn@gmail.com

ORCID: 0000-0003-1766-7031

*D. Noungoue Tchamo

Email: didetchamo@yahoo.fr

Table of contents

Table S1. Molecular weights, purity levels, and molar concentrations corresponding to the fixed screening concentration of 200 μg/mL for the isolated metabolites................................3
Figure S1: HR-ESI-MS spectrum of compound **1** 4
Figure S2: IR spectrum of compound **1** 5
Figure S3: ^{1}H NMR spectrum {500 MHz, $CDCl_3/CD_3OD$ (1:1)} of compound **1** 5
Figure S4: HSQC spectrum of compound **1**... 6
Figure S5: ^{13}C NMR spectrum {125 MHz, $CDCl_3/CD_3OD$ (1:1)} of compound **1**.................... 6
Figure S6: COSY spectrum of compound **1**... 7
Figure S7: HMBC spectrum of compound **1** 7
Figure S8: NOESY spectrum of compound **1** 8
Figure S9: ESI mass spectrum of compound **2**... 8
Figure S10: IR spectrum of compound **2** 8
Figure S11: ^{1}H NMR spectrum ($CDCl_3$, 500 MHz) of compound **2**....................................... 9
Figure S12 : HSQC spectrum of compound **2**... 9
Figure S13: ^{13}C NMR spectrum ($CDCl_3$, 125 MHz) of compound **2**.....................................10
Figure S14: DEPT spectrum in $CDCl_3$ of compound **2**...11
Figure S15: COSY spectrum of compound **2**...11

Figure S16: HMBC spectrum of compound **2** 12
Figure S17: HR-ESI-MS spectrum of compound **3** 12
Figure S18: IR spectrum of compound **3** 13
Figure S19: ^{1}H NMR (500 MHz, C_5D_5N) spectrum of compound **3**....................13
Figure S20: HSQC spectrum of compound **3**....................14
Figure S21: ^{13}C NMR (125 MHz, C_5D_5N) spectrum of compound **3** 14
Figure S22: COSY spectrum of compound **3**....................15
Figure S23: HMBC spectrum of compound **3** 15
Figure S24: HR-ESI-MS spectrum of compound **4** 16
Figure S25: LC-MS profile of Compound **4**....................16
Figure S26: IR spectrum of compound **4** 17
Figure S27: ^{1}H NMR (500 MHz, CD_3OD) spectrum of compound **4**....................17
Figure S28: ^{13}C NMR (125 MHz, CD_3OD) spectrum of compound **4**....................18
Figure S29: COSY spectrum of compound **4**....................18
Figure S30: HSQC spectrum of compound **4**....................19
Figure S31: HMBC spectrum of compound **4** 19
Figure S32: Compared ^{13}C NMR (125 MHz, CD_3OD) spectrum of compound **4** and **3** {125 MHz, $CDCl_3/CD_3OD$ (1:1)} 20
Figure S33: Extended ^{13}C NMR (125 MHz, CD_3OD) spectrum of compound **4**....................20
Figure S34: ^{1}H NMR spectra of stereoisomers of pinellic acid. 21
Figure S35: ^{1}H NMR spectrum (Acetone-d_6, 500 MHz) of the mixture of **5** and **6**....................21
Figure S36: ^{13}C NMR spectrum (Acetone-d_6, 125 MHz) of the mixture of **5** and **6** 22
Figure S37: ^{1}H NMR spectrum ($CDCl_3$, 500 MHz) of **7** 22
Figure S38: ^{13}C NMR spectrum ($CDCl_3$, 125 MHz) of **7** 22
Figure S39: HR-ESI-MS of **8** 23
Figure S40: ^{1}H NMR spectrum (CD_3OD, 500 MHz) of **8** 23
Figure S41: ^{13}C NMR spectrum (CD_3OD, 125 MHz) of **8** 23
Figure S42: ^{1}H NMR spectrum ($CDCl_3/CD_3OD$, 600 MHz) of **9** 24
Figure S43: ^{13}C NMR spectrum ($CDCl_3/CD_3OD$, 150 MHz) of **9**....................24
Figure S44: ^{1}H NMR spectrum (DMSO-d_6, 500 MHz) of **10** 25
Figure S45: ^{13}C NMR spectrum (DMSO-d_6, 125 MHz) of **10**....................25
Figure S46: ^{1}H NMR spectrum of **11** (500 MHz, DMSO-d_6) 25
Figure S47: ^{13}C NMR spectrum of **11** (125 MHz, DMSO-d_6)....................26
Figure S48: HR-ESI-MS of **12** 26
Figure S49: ^{1}H NMR spectrum ($CDCl_3/CD_3OD$, 600 MHz) of **12**....................26
Figure S50: ^{13}C NMR spectrum {150 MHz, $CDCl_3/CD_3OD$ (1:1)} of **12** 27
Figure S51: ^{1}H NMR spectrum (DMSO-d_6, 500 MHz) of **13** 27
Figure S52: ^{13}C NMR spectrum (DMSO-d_6, 125 MHz) of **13**....................27
Figure S53: ^{1}H NMR spectrum (DMSO-d_6, 500 MHz) of **14** 28
Figure S54: ^{13}C NMR spectrum (DMSO-d_6, 125 MHz) of **14**....................28
Figure S55: GC/EIMS pic **1** of **15** 29
Figure S56: GC/EIMS pic **2** of **16** 29
Figure S57: GC/EIMS pic **3** of **17** 30
Figure S58: ^{1}H NMR spectrum ($CDCl_3$, 500 MHz) of the mixture of **15**, **16** and **17**....................30
Figure S59: ^{13}C NMR spectrum ($CDCl_3$, 125 MHz) of the mixture of **15**, **16** and **17** 30

Scheme 1: mass fragmentation pattern of **3** 16

Table S1. Molecular weights, purity levels, and molar concentrations corresponding to the fixed screening concentration of 200 µg/mL for the isolated metabolites.

Compound	Structure	Molecular weight (g/mol)	Estimated purity (%)	Screening concentration (µg/mL)	Equivalent concentration (µM)
Celtic acid	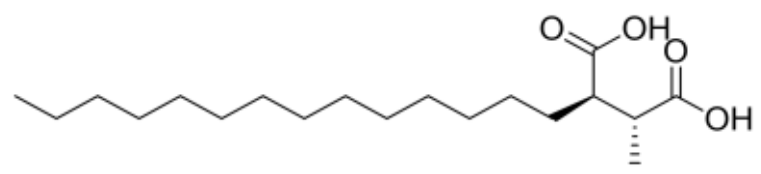	328.4867	> 95	200	608.85
Milbraedic acid	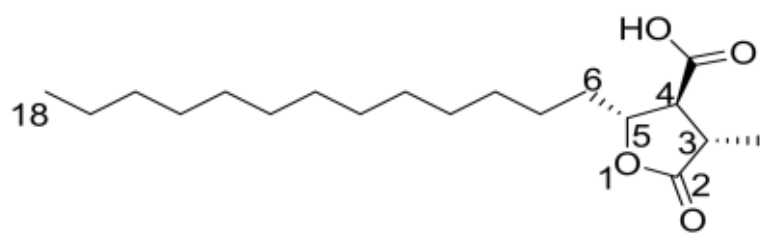	326.4709	> 95	200	612.61
(9*R*, 10*E*, 12*R*, 13*S*)-9, 12, 13-Trihydroxy-10-octadecanoic acid	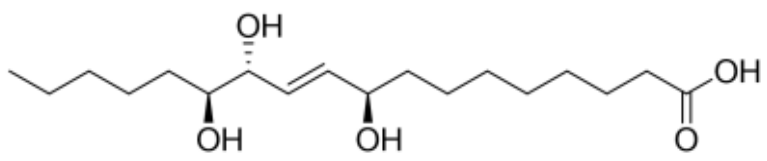	330.4596	> 95	200	605.22
Niemeyong acid dimer	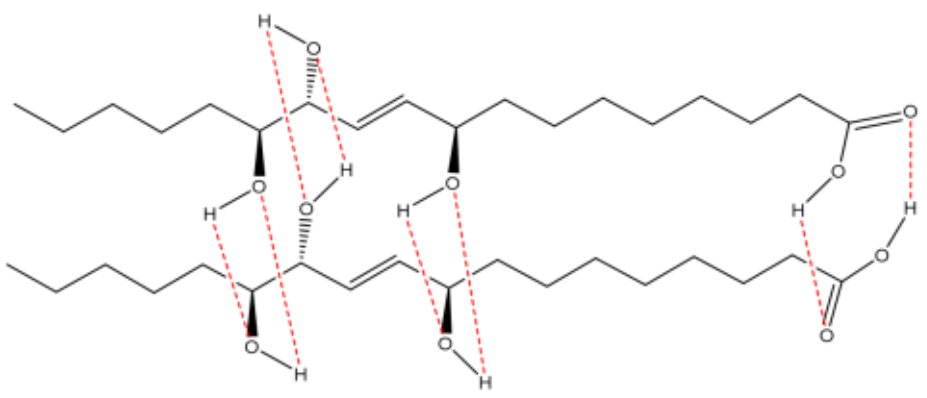	660.9191	> 95	200	302.61
Mixture of Ursolic (5) and Oleanolic acid (6)	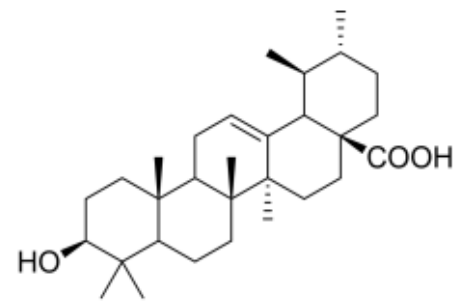 **5** 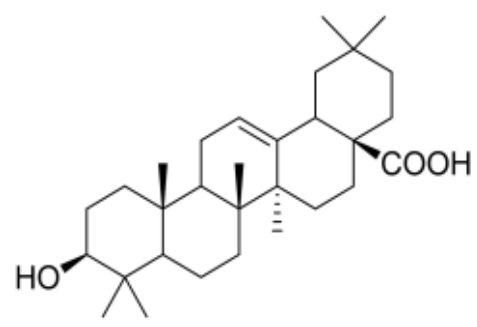 **6**	-	Mixture of two compounds	200	-
Lichexanthone	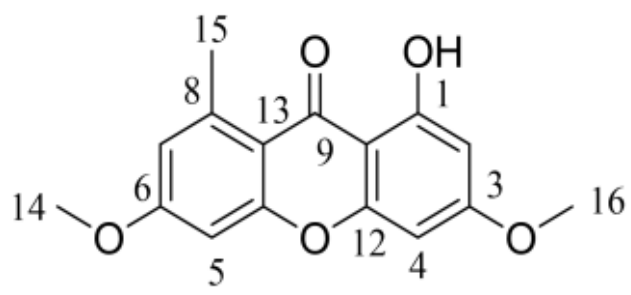	286.2794	> 95	200	698.62
Azelaic acid	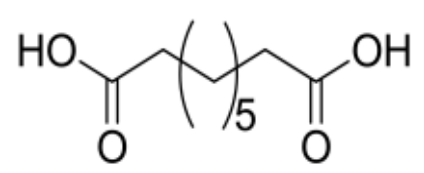	132.1146	> 95	200	1513.84
*N-Trans-p-*coumaroyloctopamine	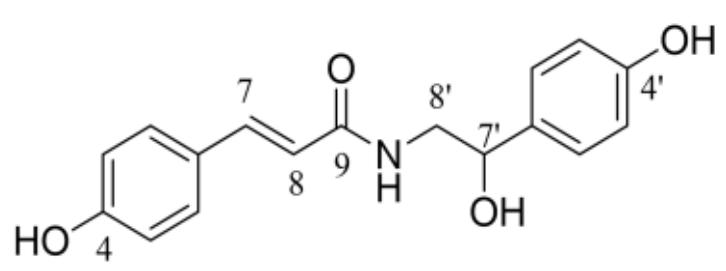	299.3212	> 95	200	668.18
Allantoin	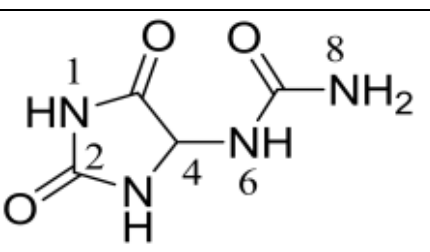	158.1154	> 95	200	1264.90

Corosolic acid	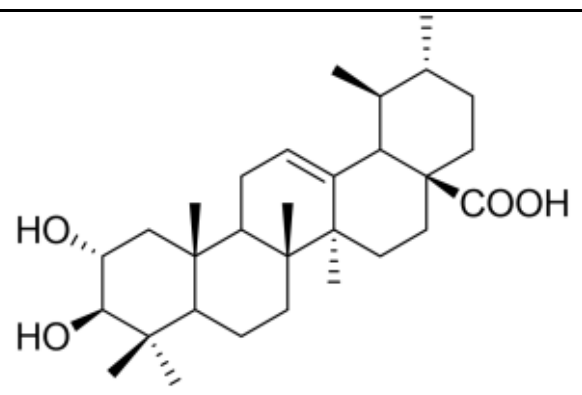	472.6997	> 95	200	423.10
Sebacic acid	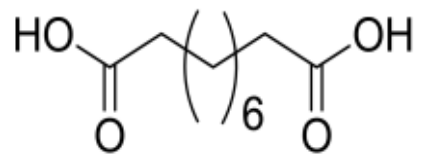	132.1146	> 95	200	1513.84
Ethyl gallate	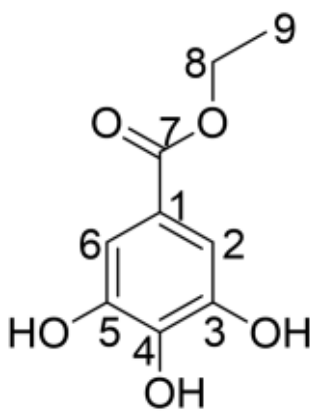	198.1727	> 95	200	1009.22
Gallic acid	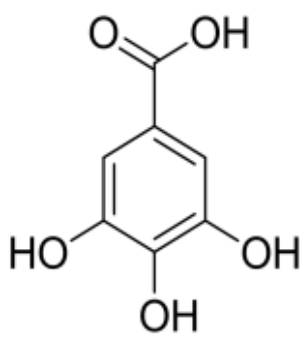	170.1195	> 95	200	1175.64
Mixture of palmitic acid (15), stearic acid (17) and *Cis*-11 vaccenic acid (16)	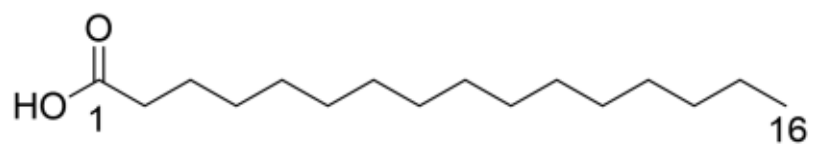 **15** 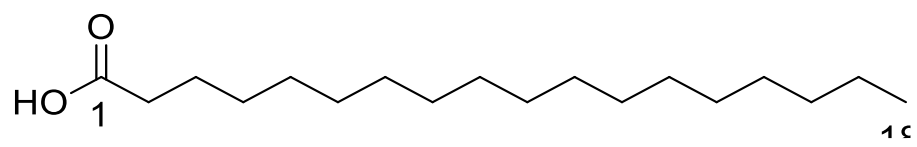 **16** 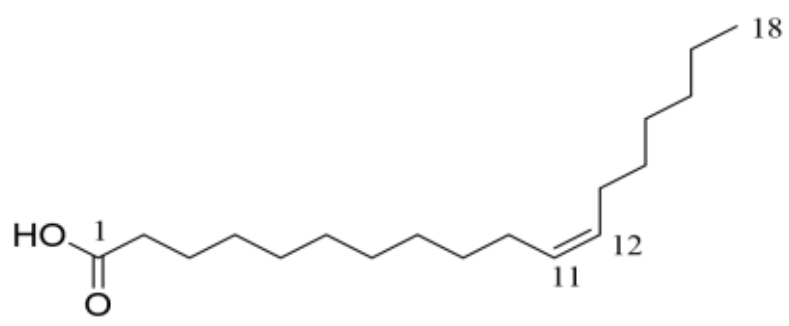 **17**	-	Mixture of tree compounds	200	-

Purity was estimated based on ESI-MS and one-dimensional NMR data.

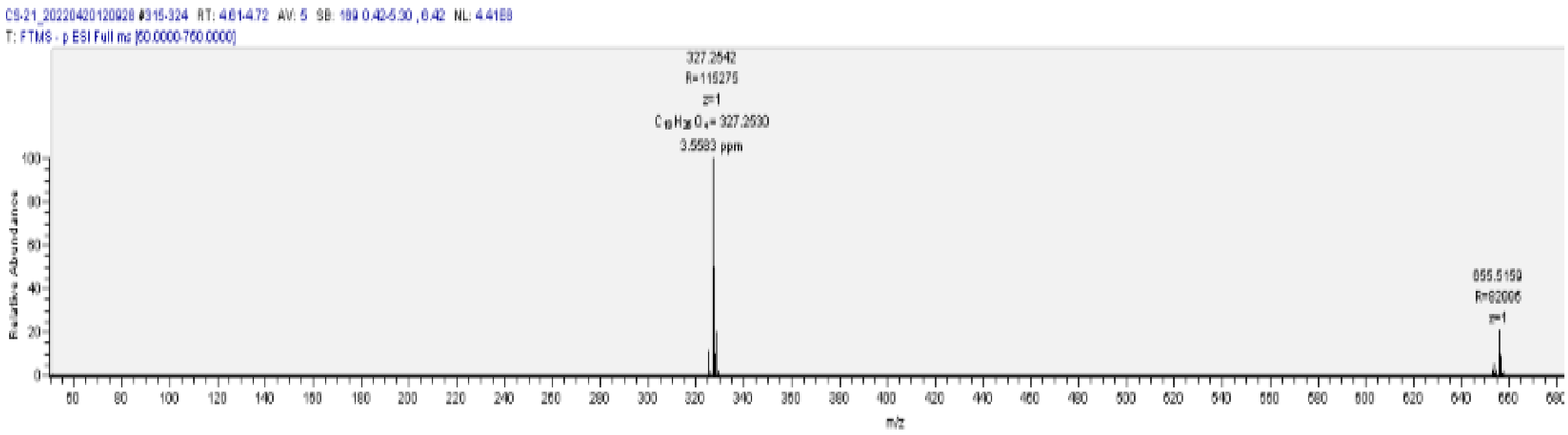

Figure S1: HR-ESI-MS spectrum of compound **1**

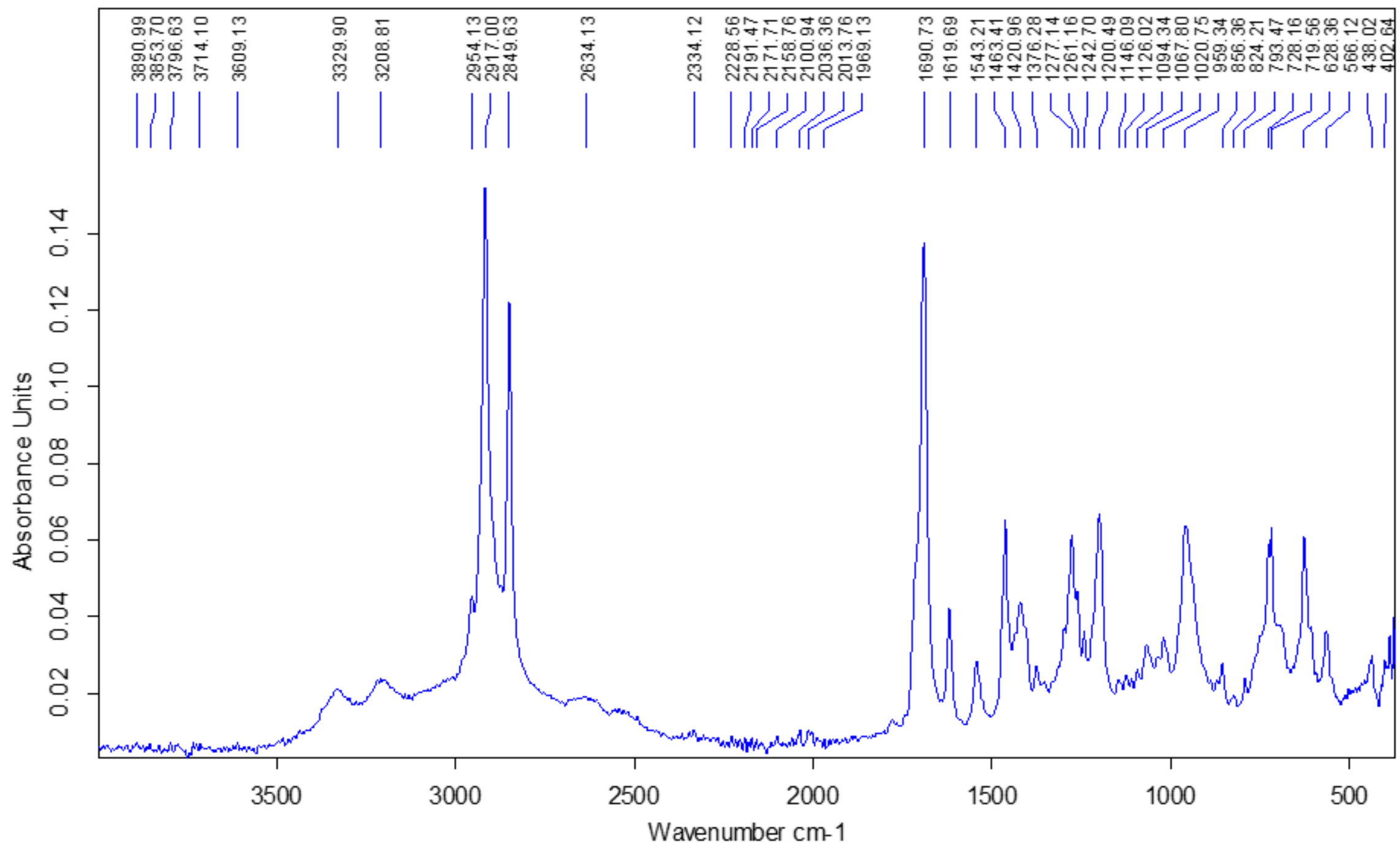

Figure S2: IR spectrum of compound **1**

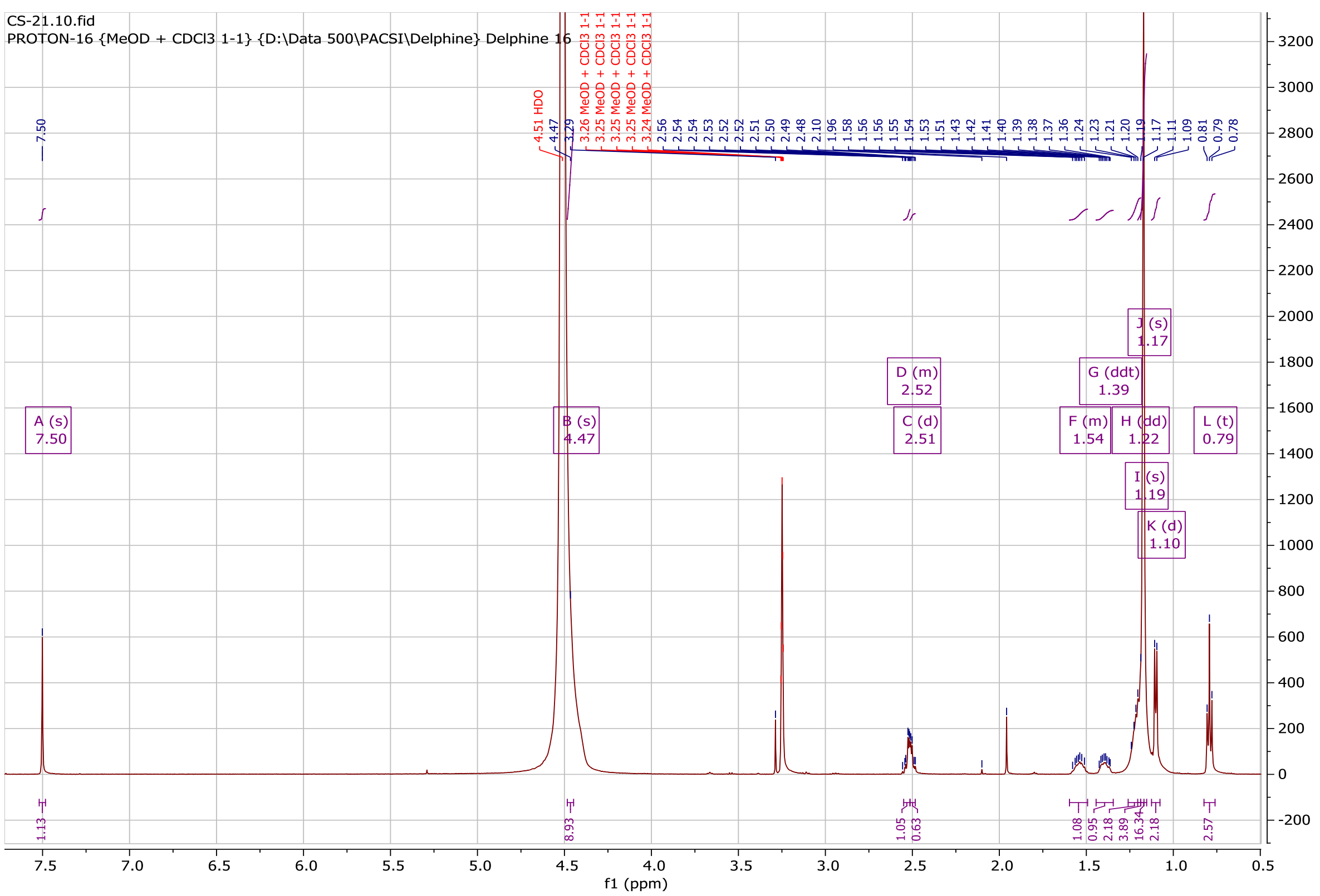

Figure S3: ^{1}H NMR spectrum {500 MHz, $CDCl_3/CD_3OD$ (1:1)} of compound **1**

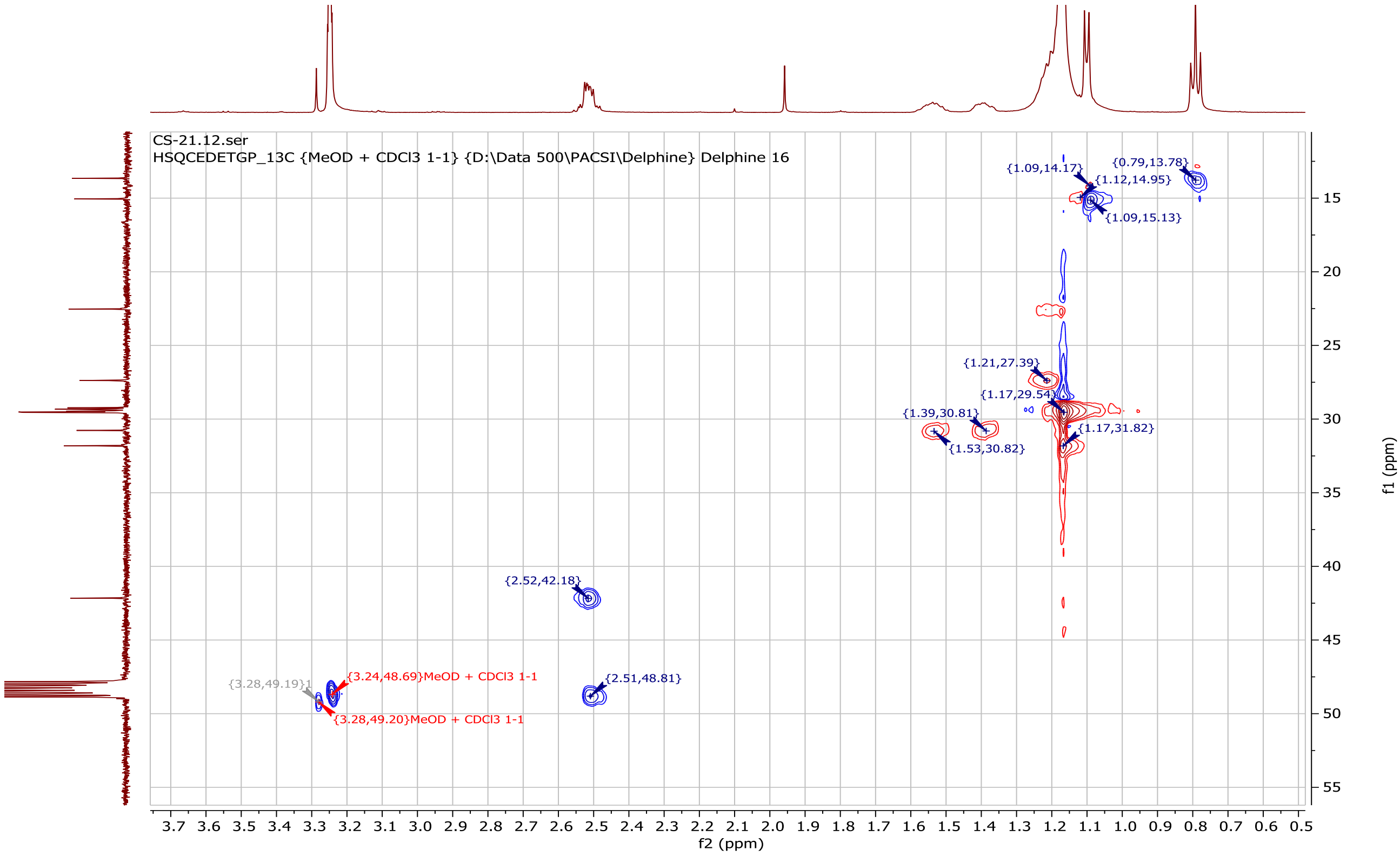

Figure S4: HSQC spectrum of compound 1

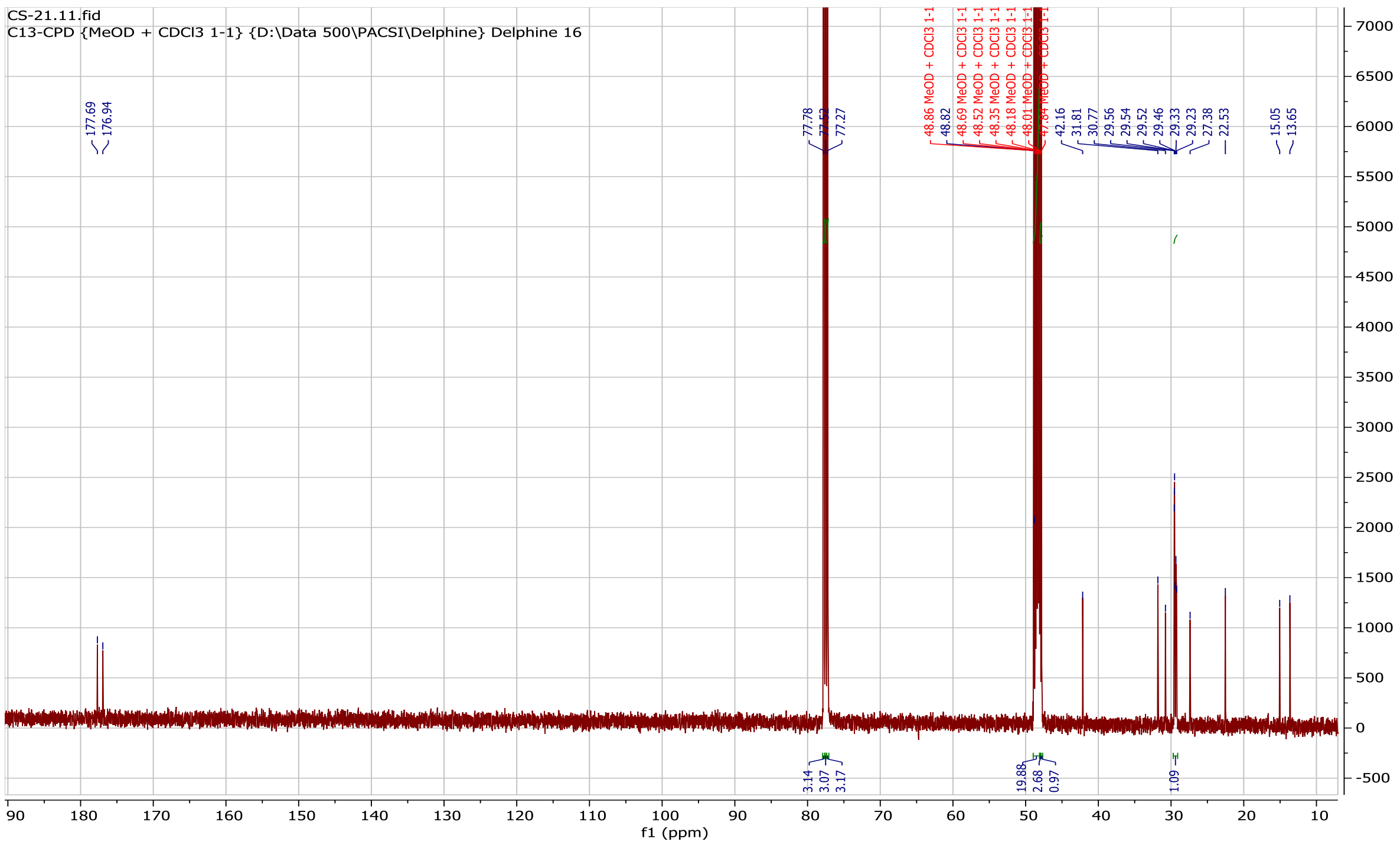

Figure S5: ^{13}C NMR spectrum {125 MHz, $CDCl_3/CD_3OD$ (1:1)} of compound **1**

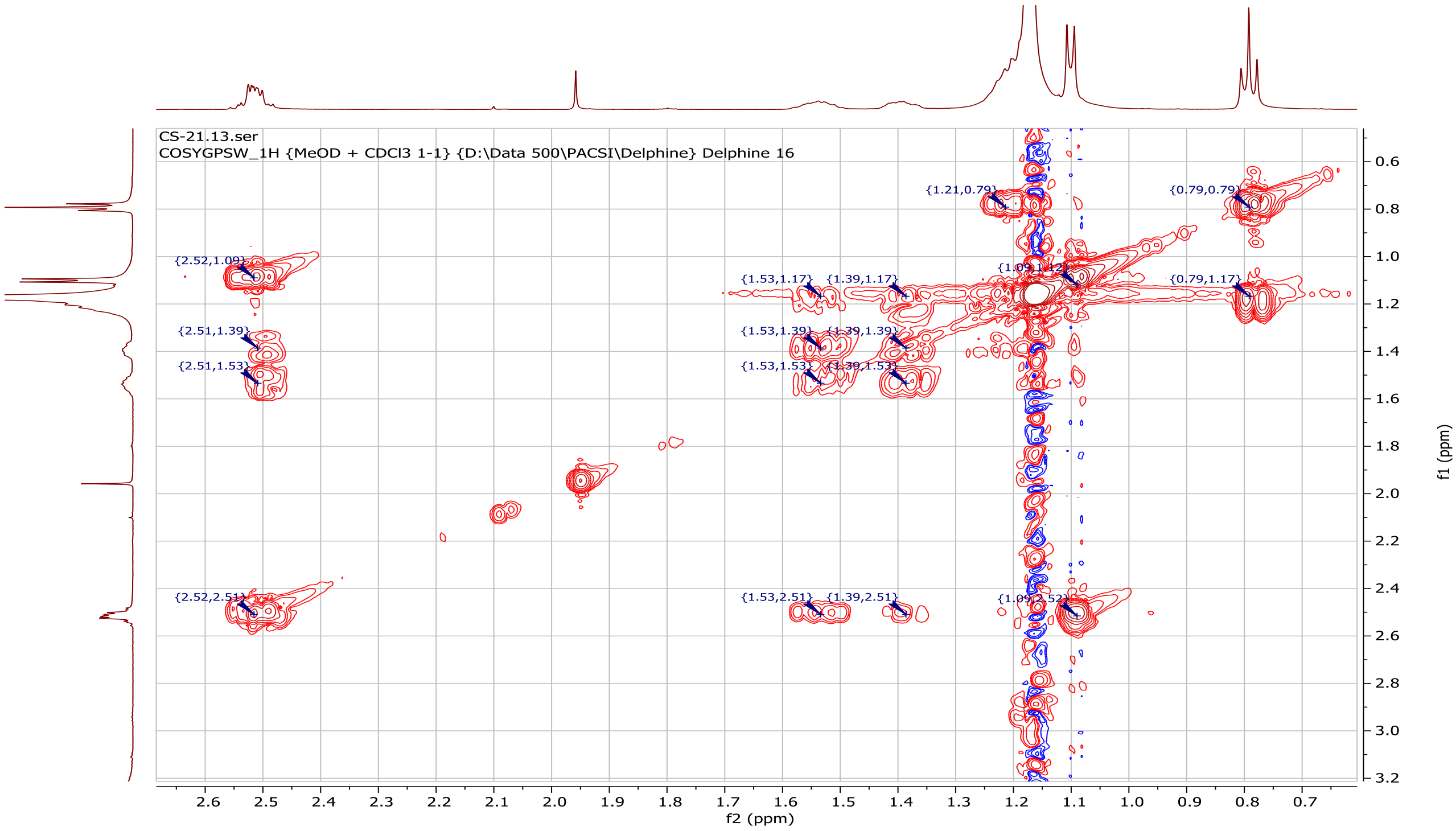

Figure S6: COSY spectrum of compound **1**

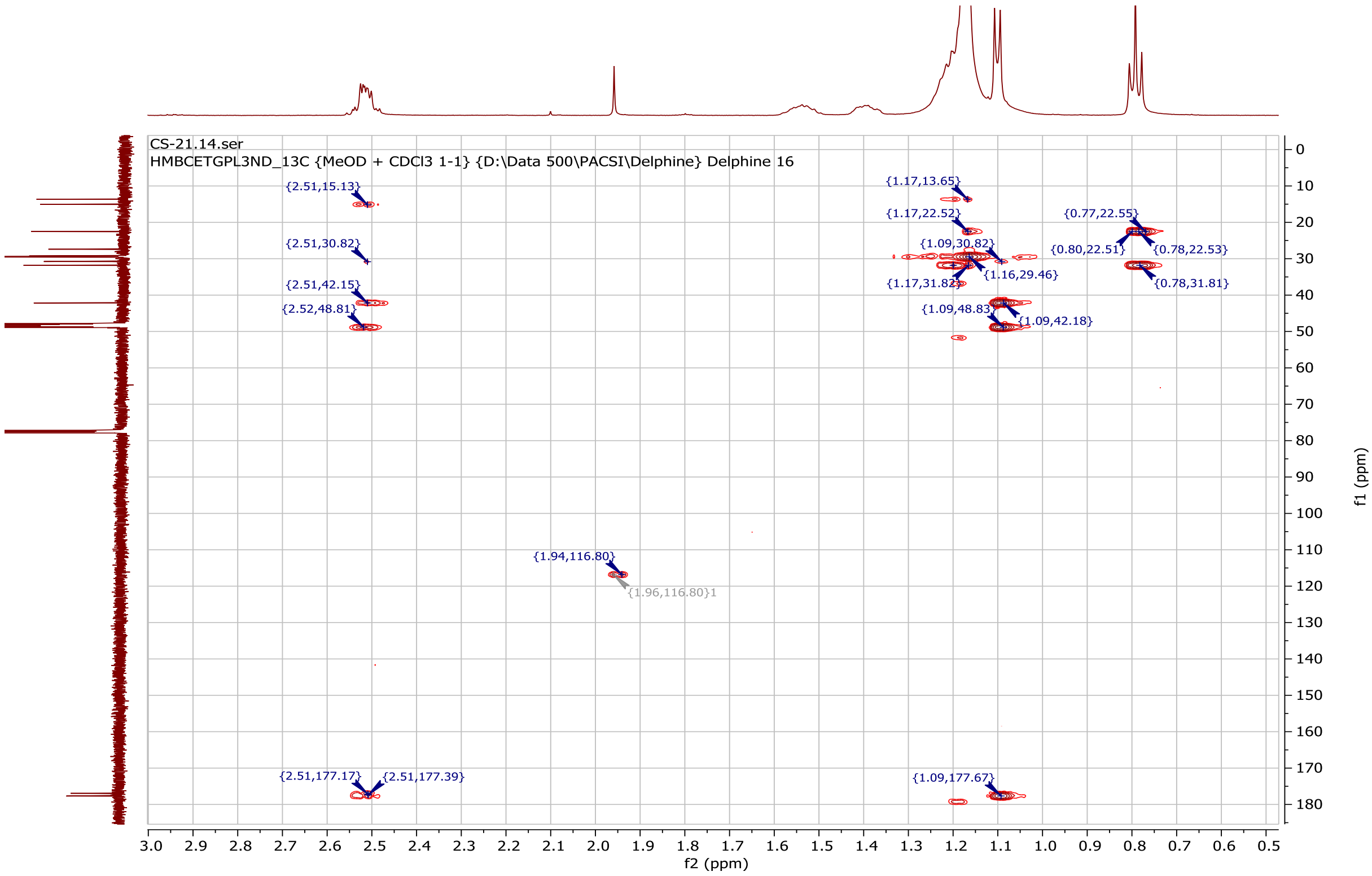

Figure S7: HMBC spectrum of compound **1**

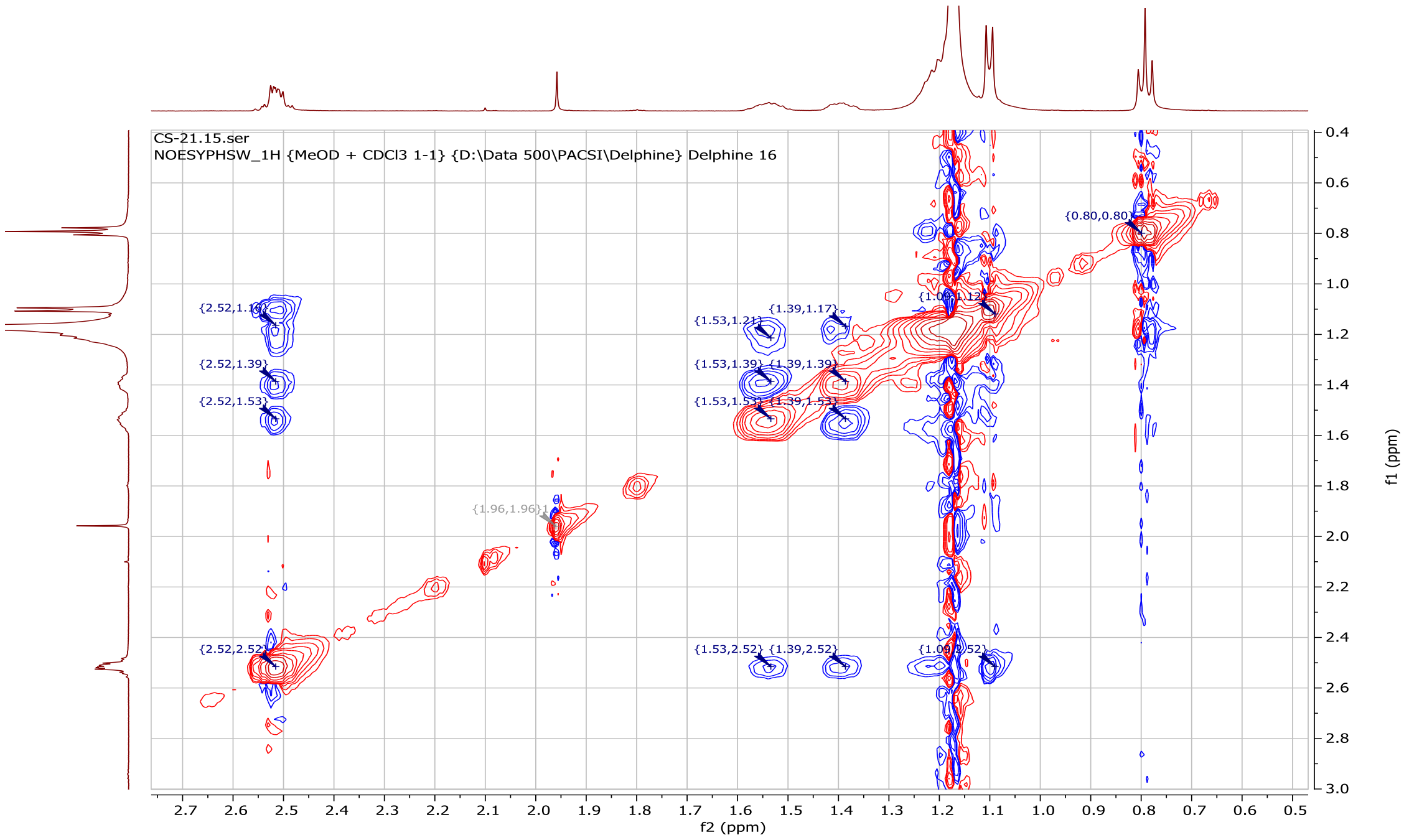

Figure S8: NOESY spectrum of compound **1**

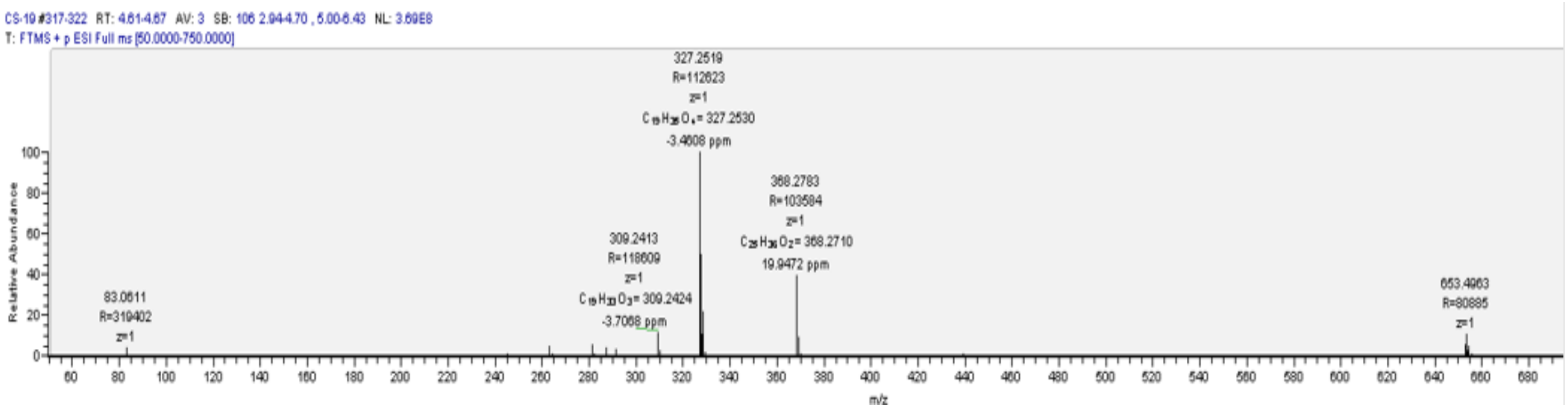

Figure S9: ESI mass spectrum of compound **2**

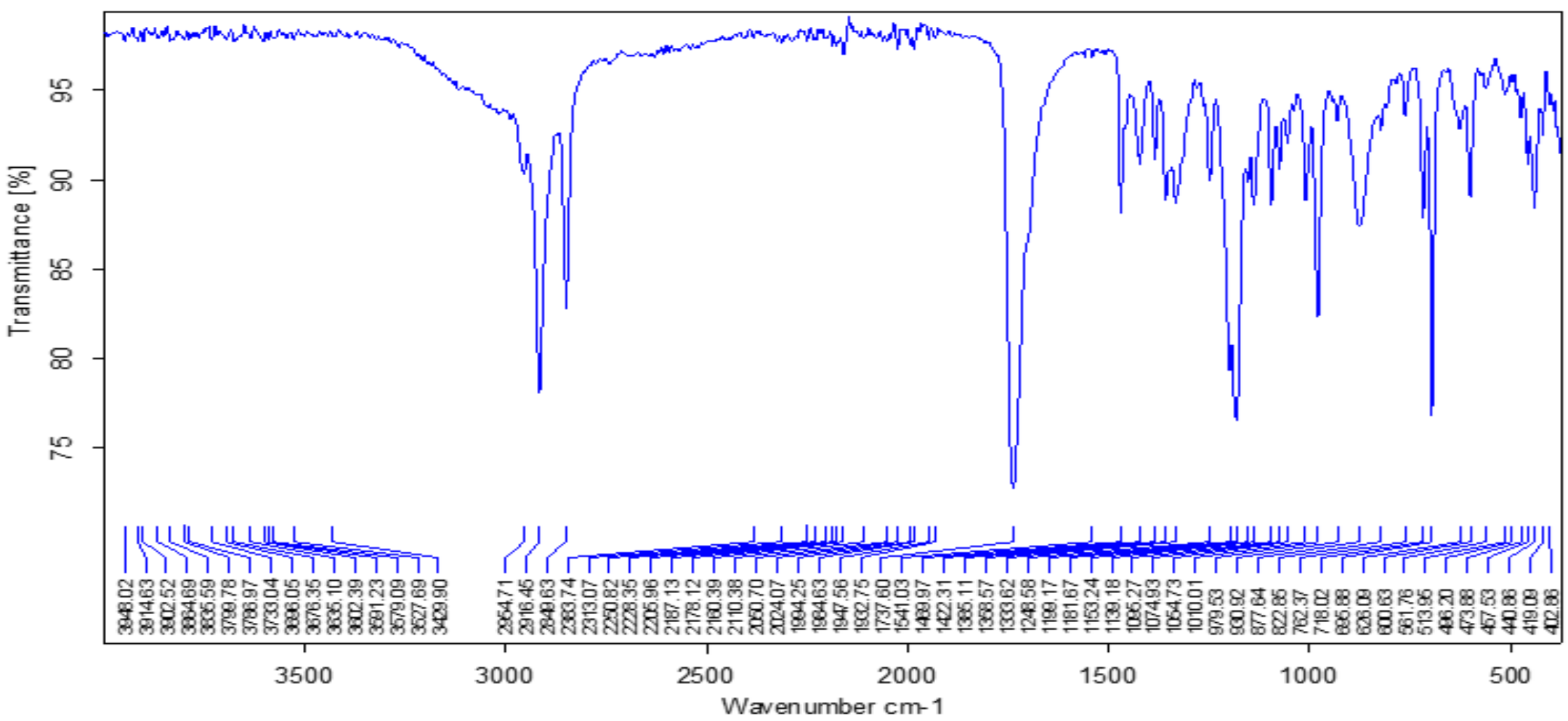

Figure S10: IR spectrum of compound **2**

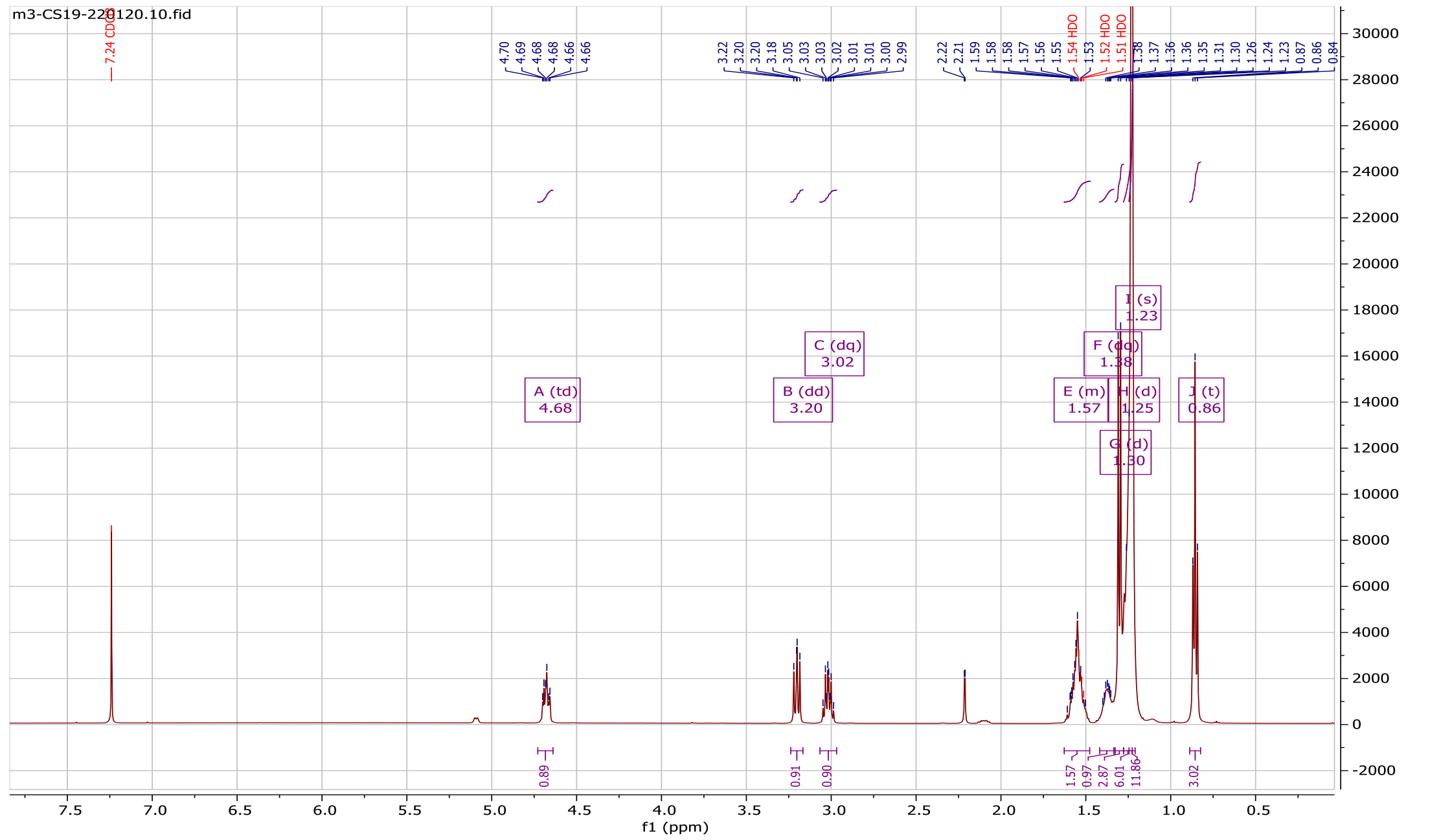

Figure S11: ^1H NMR spectrum ($CDCl_3$, 500 MHz) of compound **2**

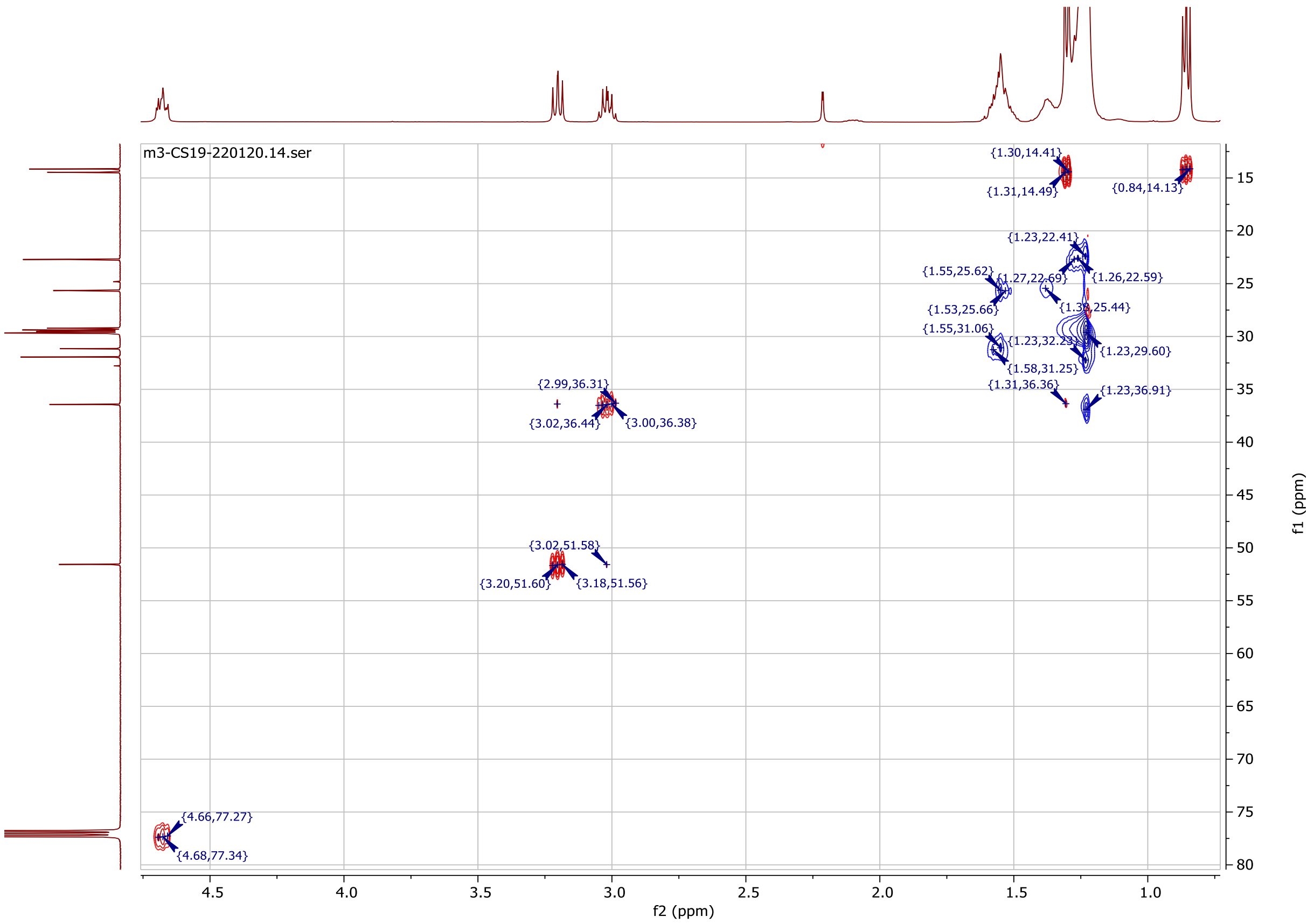

Figure S12 : HSQC spectrum of compound **2**

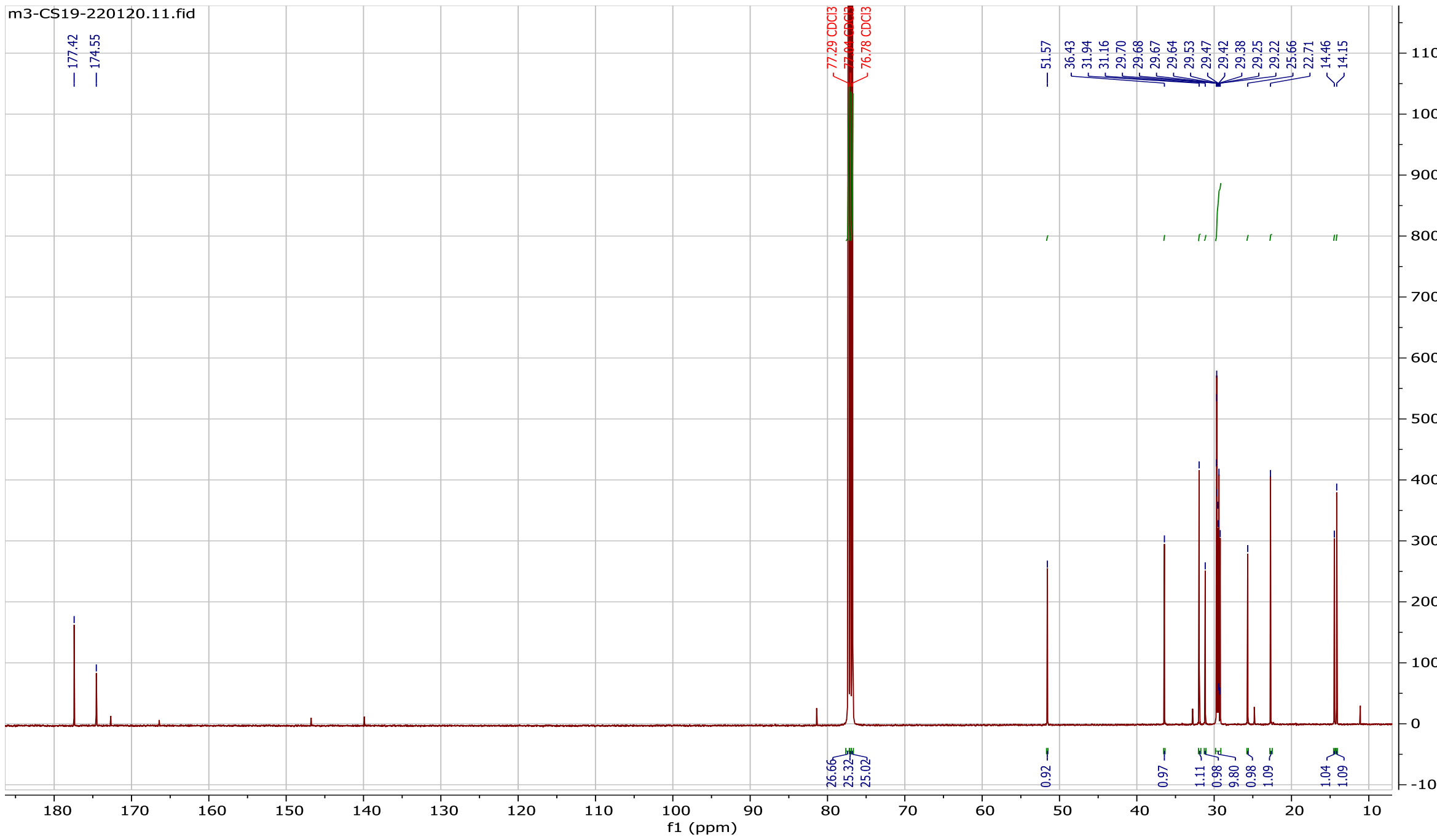

Figure S13: ^{13}C NMR spectrum ($CDCl_3$, 125 MHz) of compound **2**

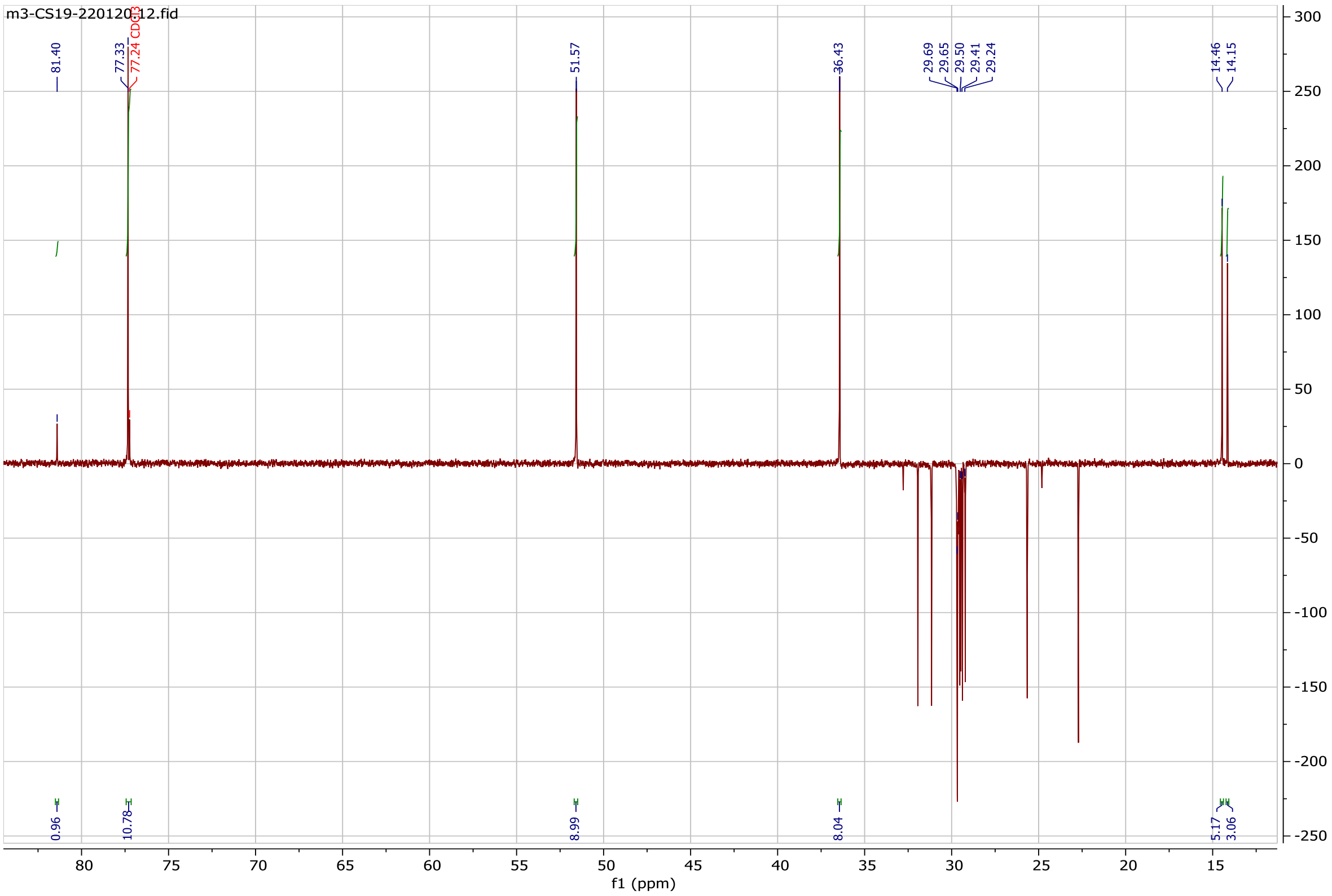

Figure S14: DEPT spectrum in $CDCl_3$ of compound **2**

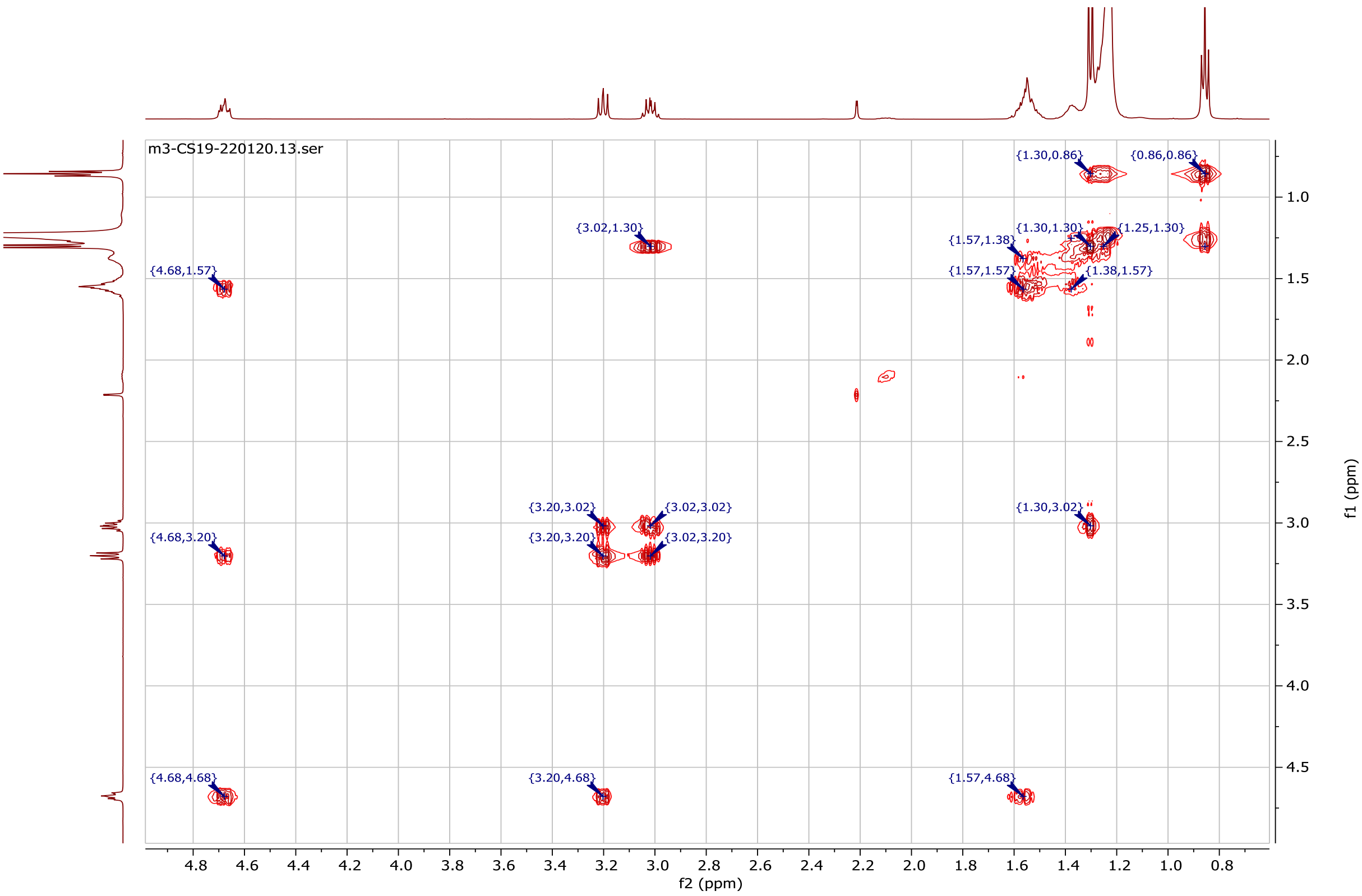

Figure S15: COSY spectrum of compound **2**

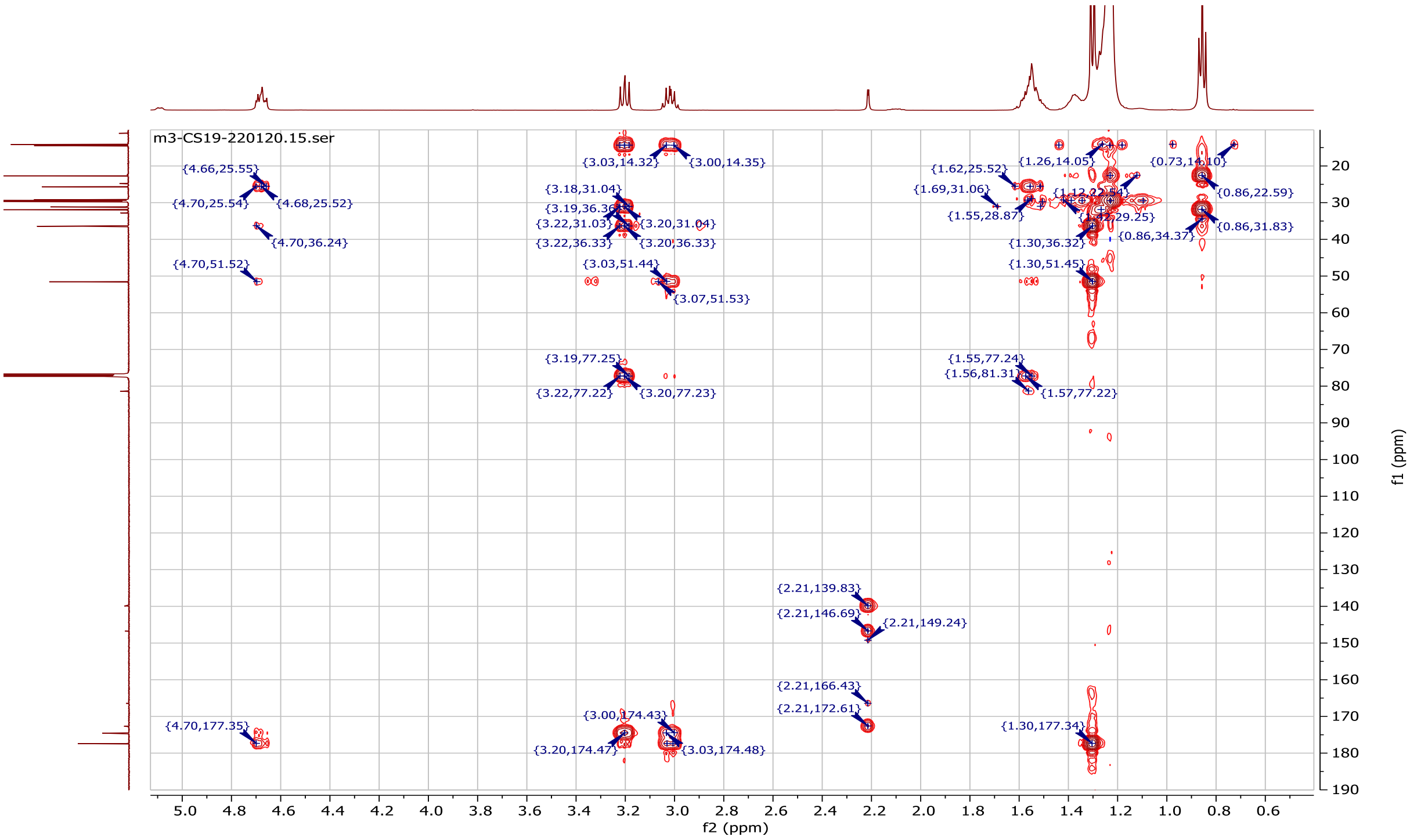

Figure S16: HMBC spectrum of compound **2**

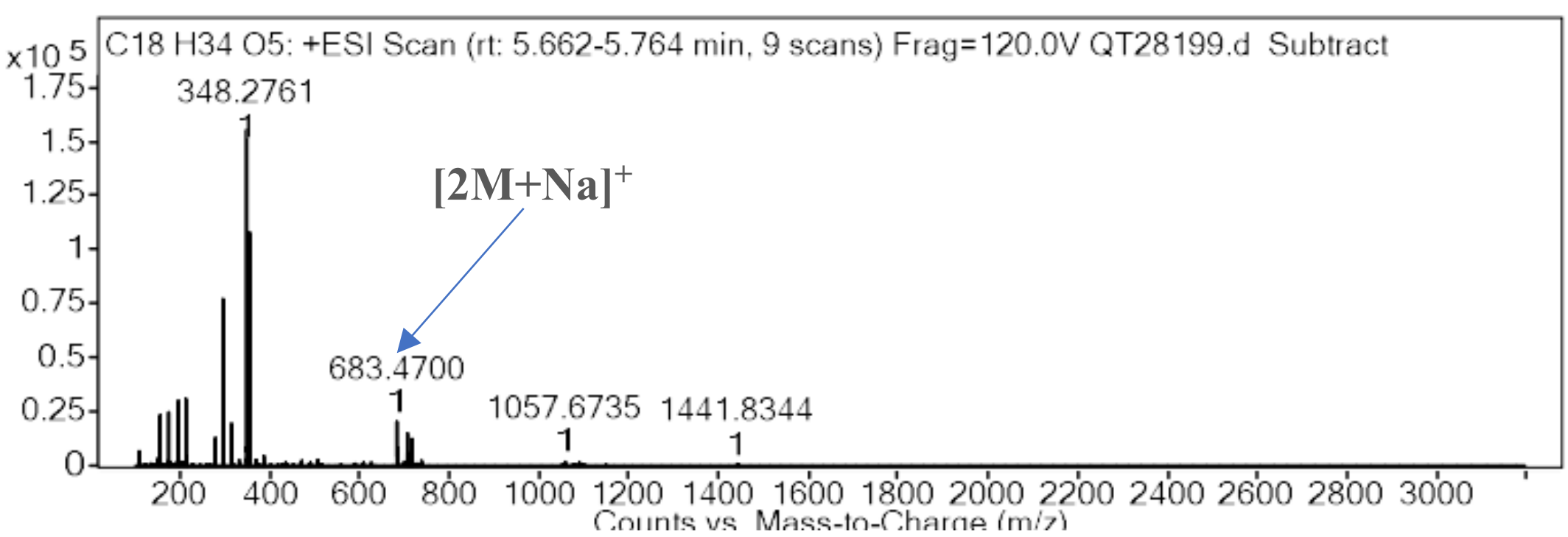

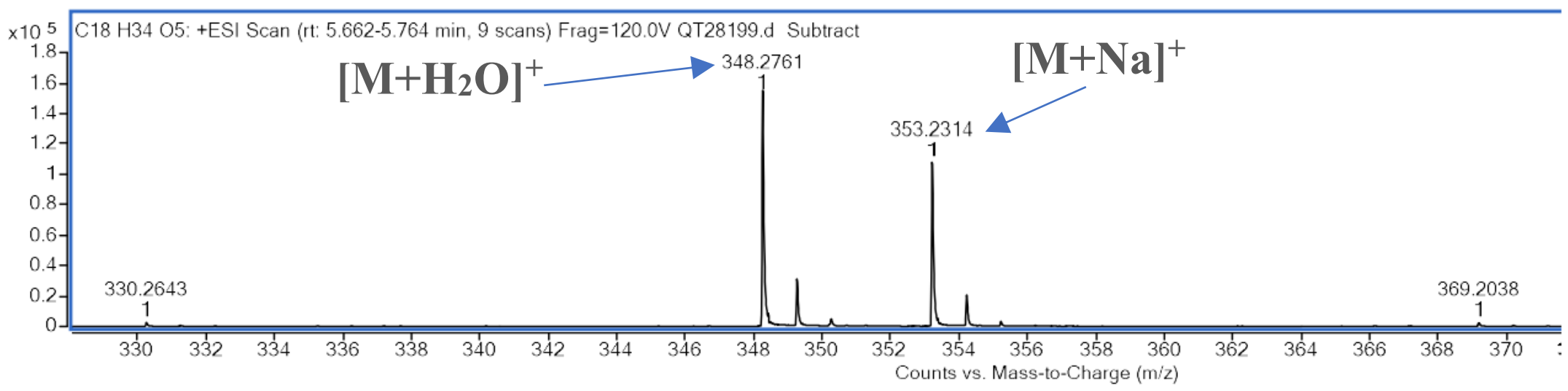

Peak List

m/z	z	Abund	Formula	Ion
155.1066	1	24218.87		
173.1172	1	24674.82		
195.1382	1	30049.26		
213.1488	1	31453.81		
295.2278	1	78391.83		
348.2761	1	160452.01	C18 H34 O5	(M+NH4)+
349.2786	1	31544.85	C18 H34 O5	(M+NH4)+
353.2314	1	111390.33	C18 H34 O5	(M+Na)+
354.2344	1	20882.05	C18 H34 O5	(M+Na)+
683.47	1	20763.61		

Figure S17: HR-ESI-MS spectrum of compound **3**

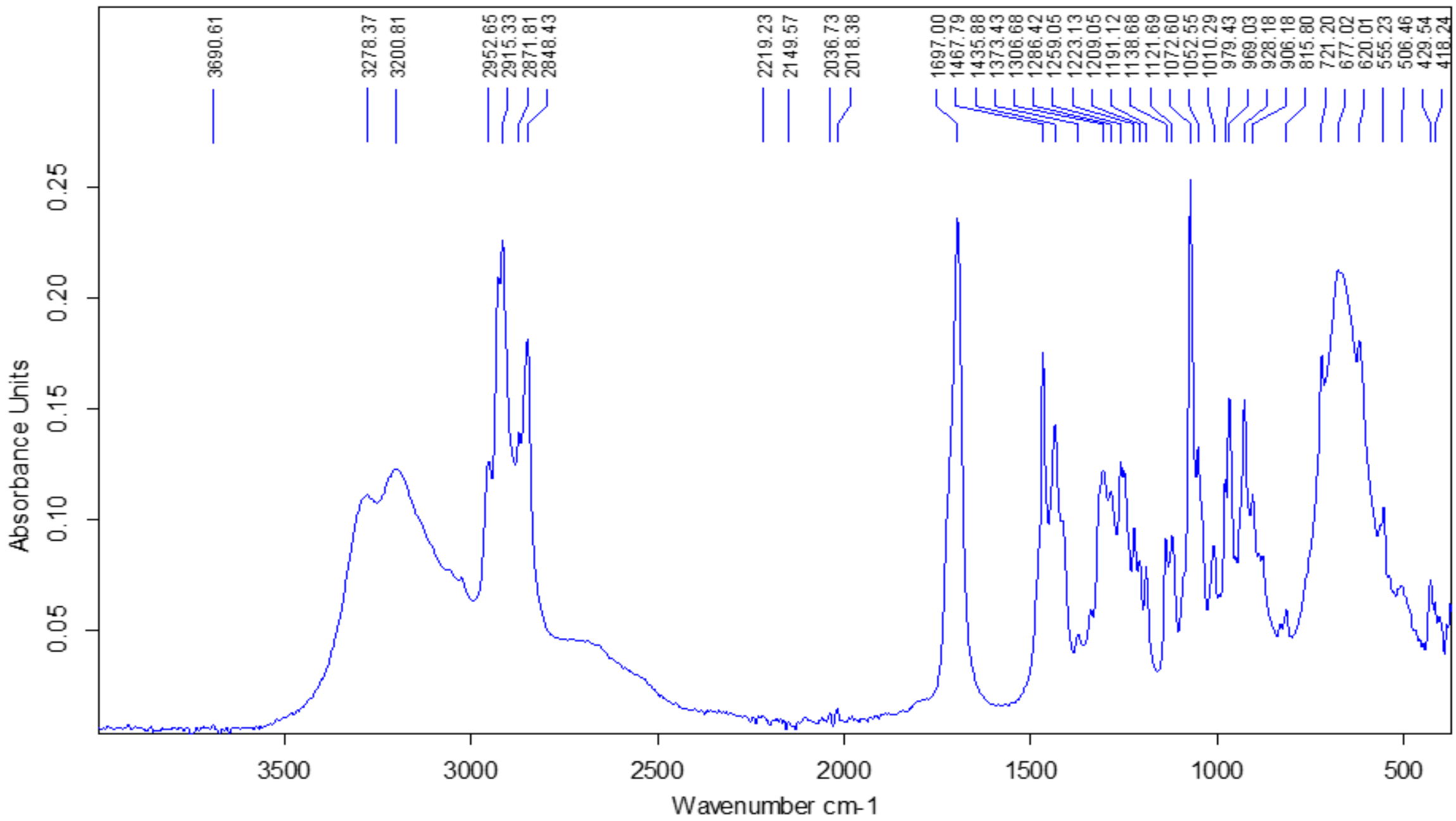

Figure S18: IR spectrum of compound **3**

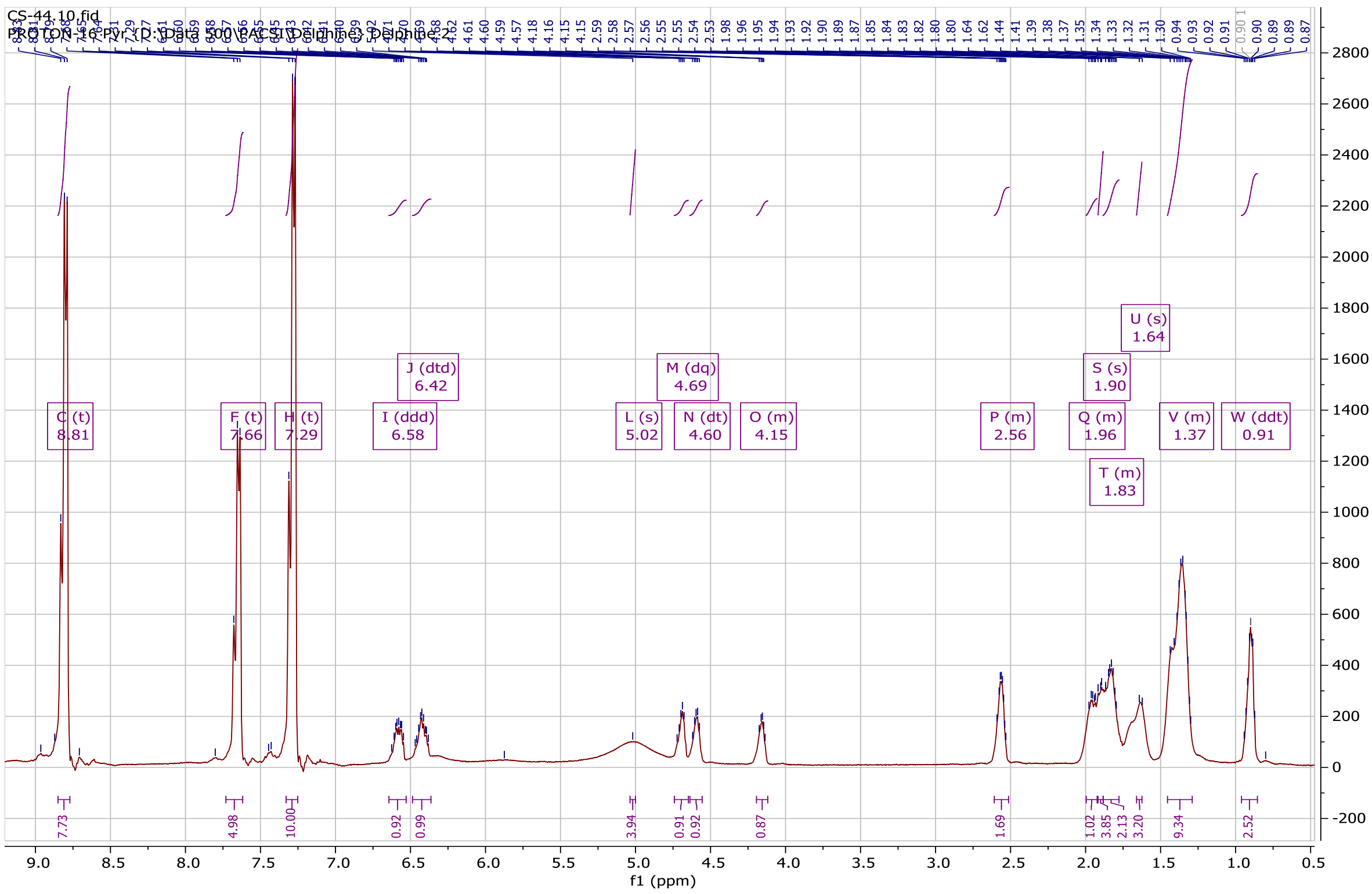

Figure S19: ^{1}H NMR (500 MHz, C_5D_5N) spectrum of compound **3**

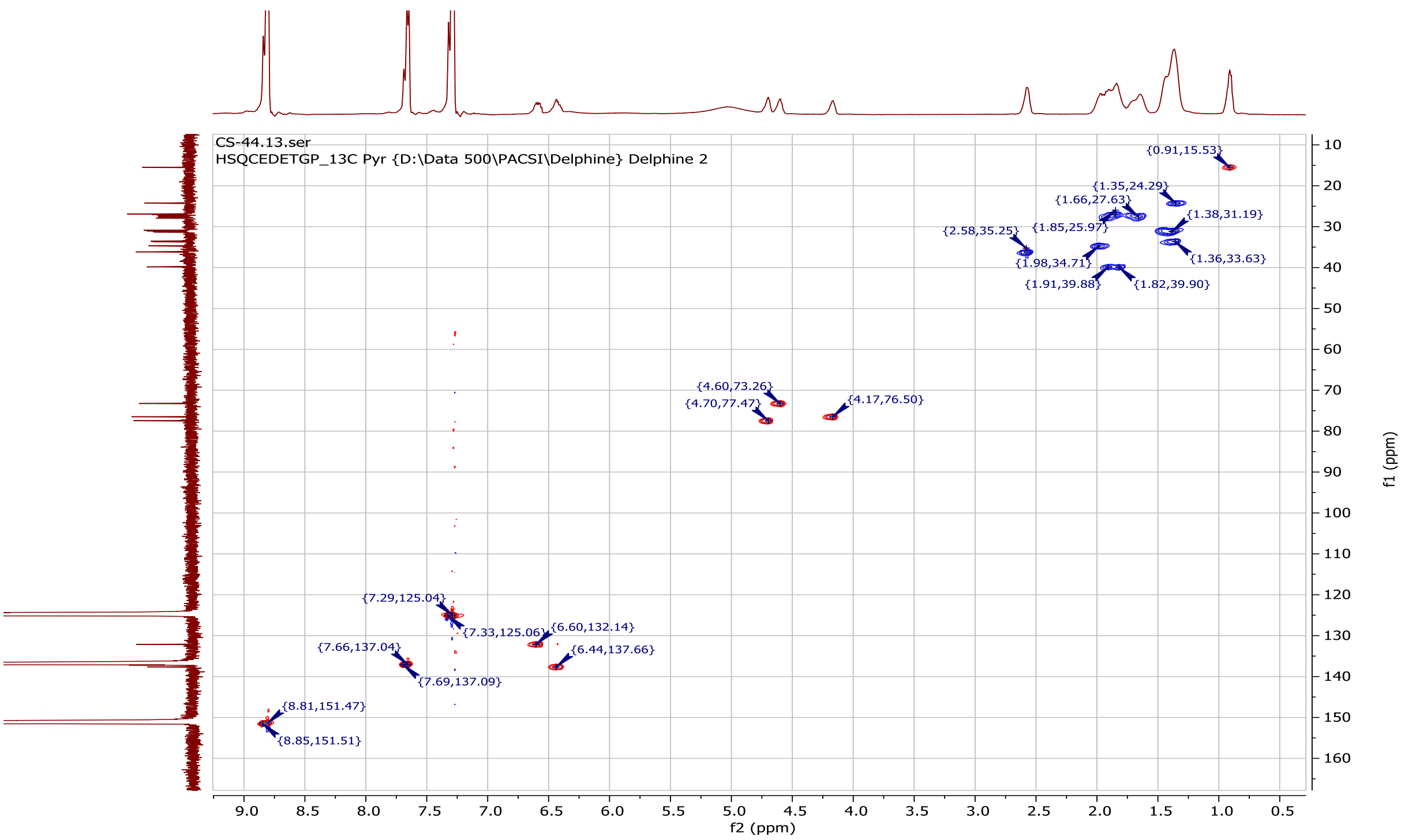

Figure S20: HSQC spectrum of compound **3**

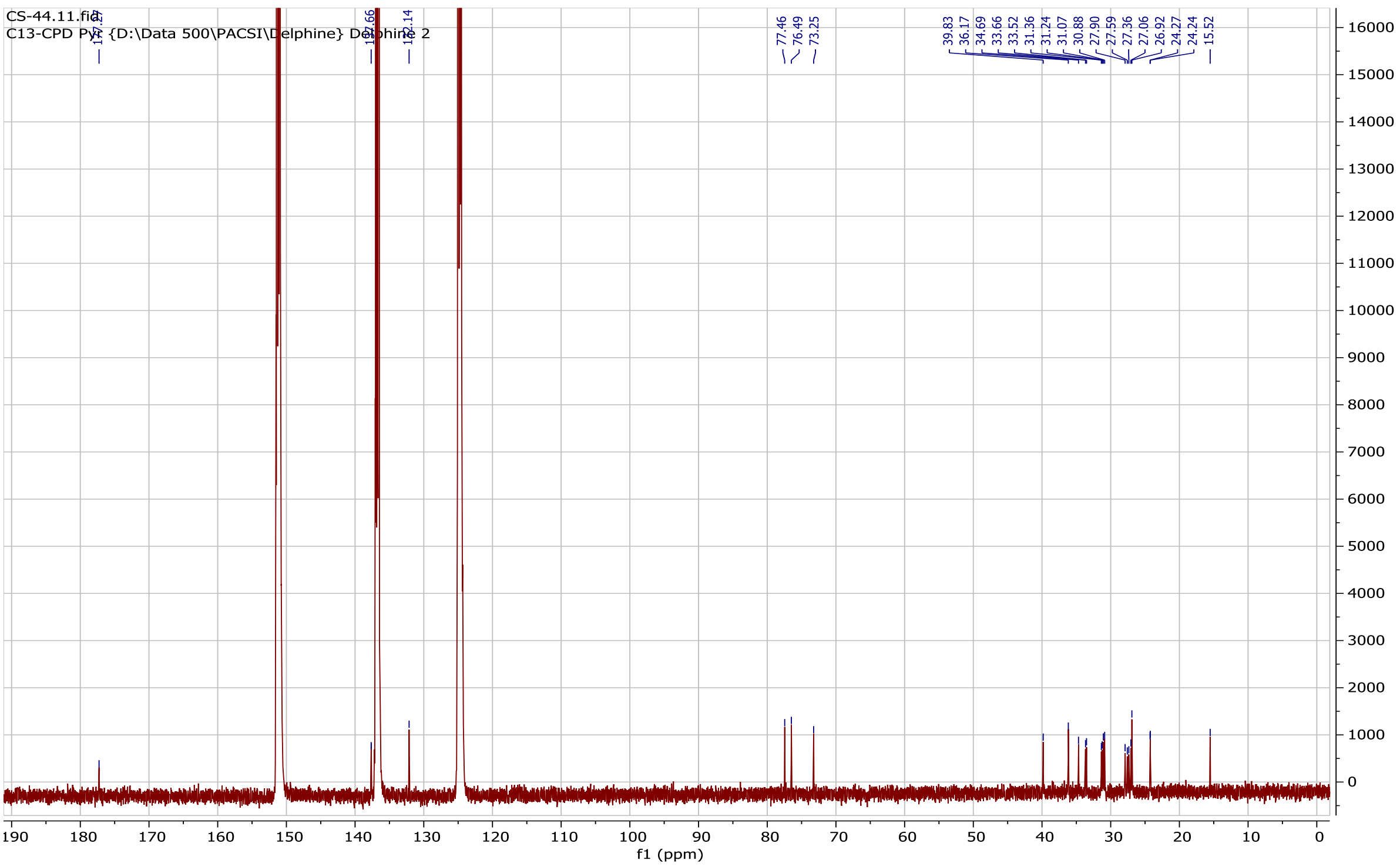

Figure S21: ^{13}C NMR (125 MHz, C_5D_5N) spectrum of compound **3**

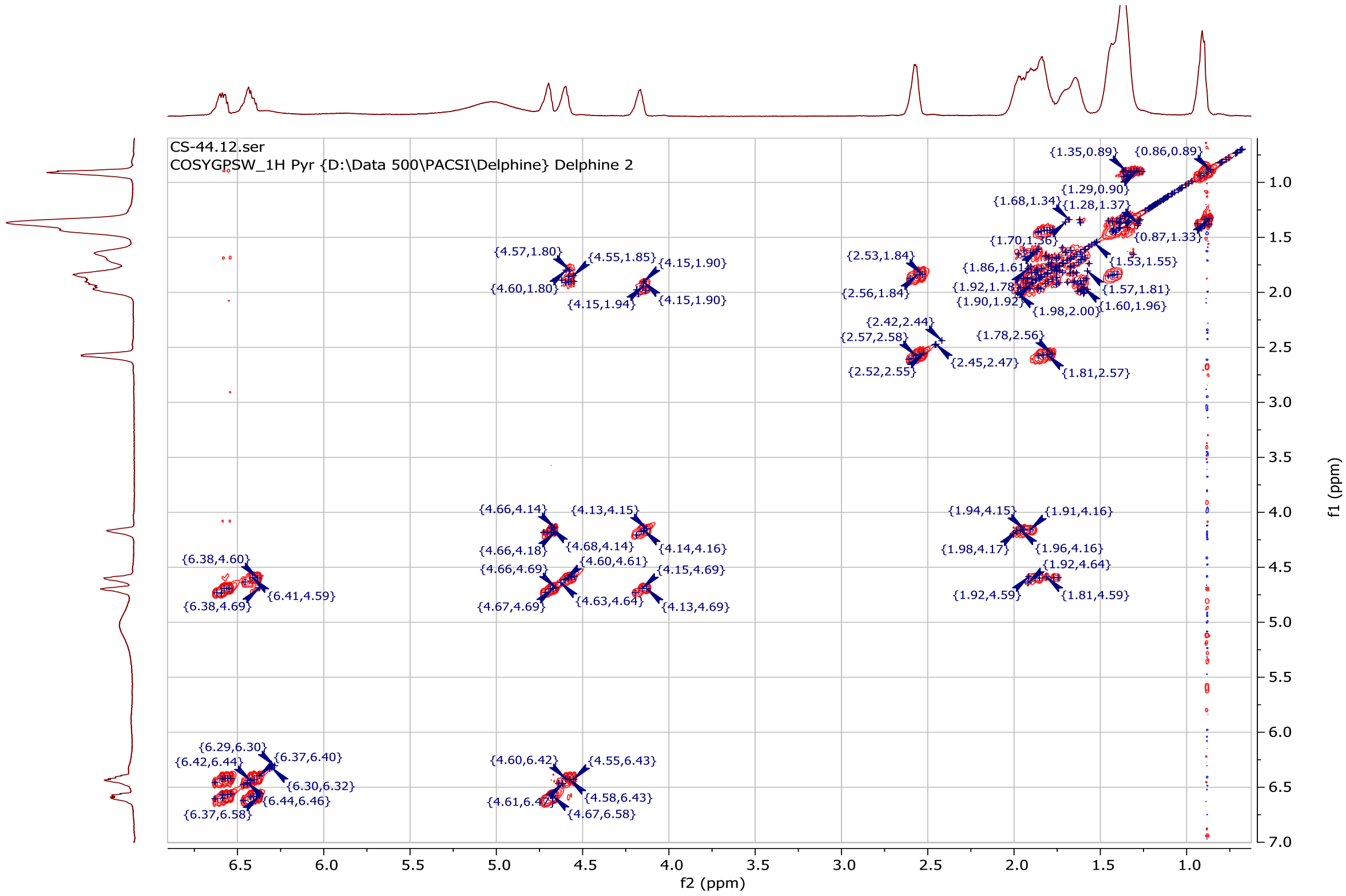

Figure S22: COSY spectrum of compound **3**

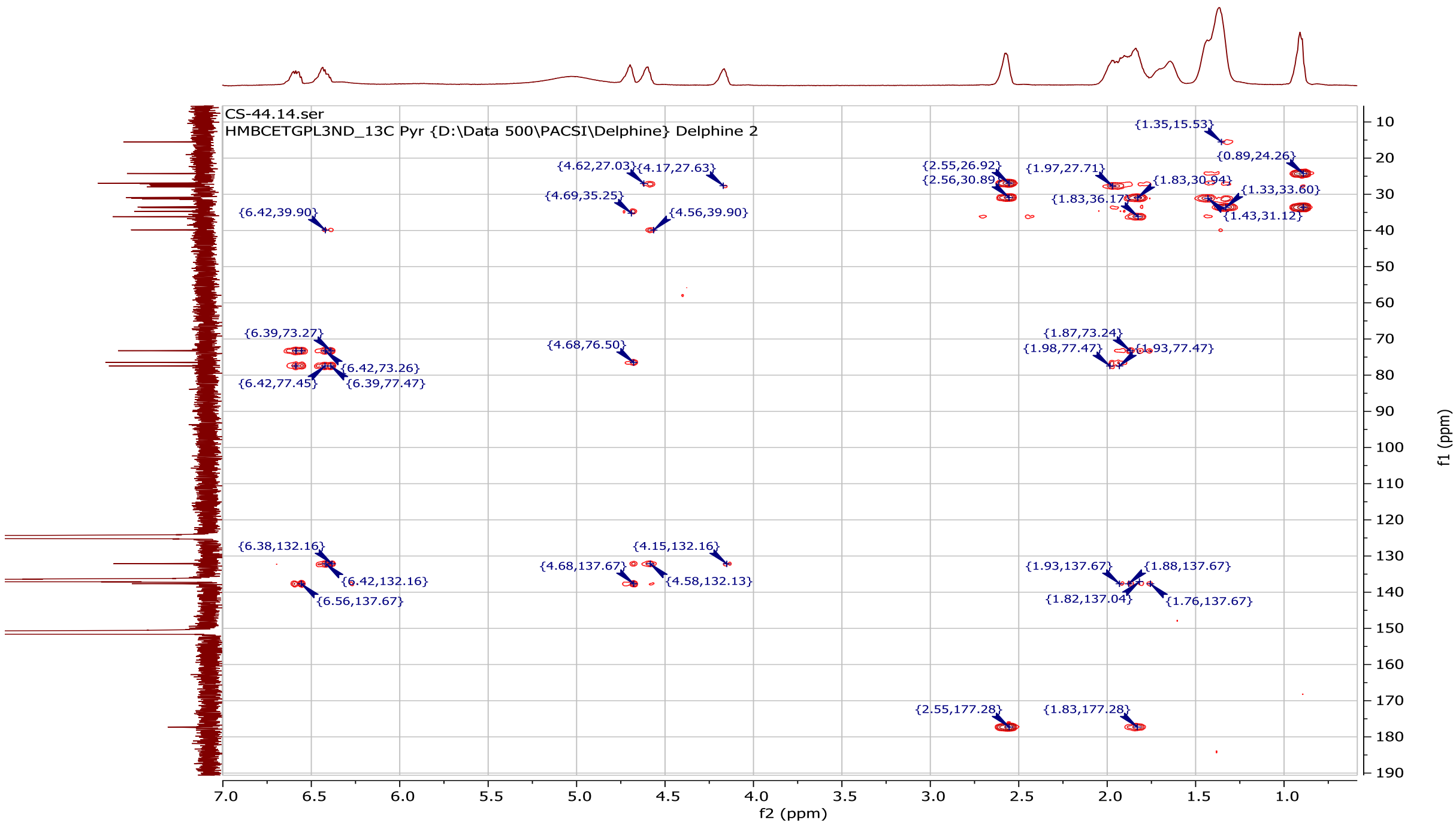

Figure S23: HMBC spectrum of compound **3**

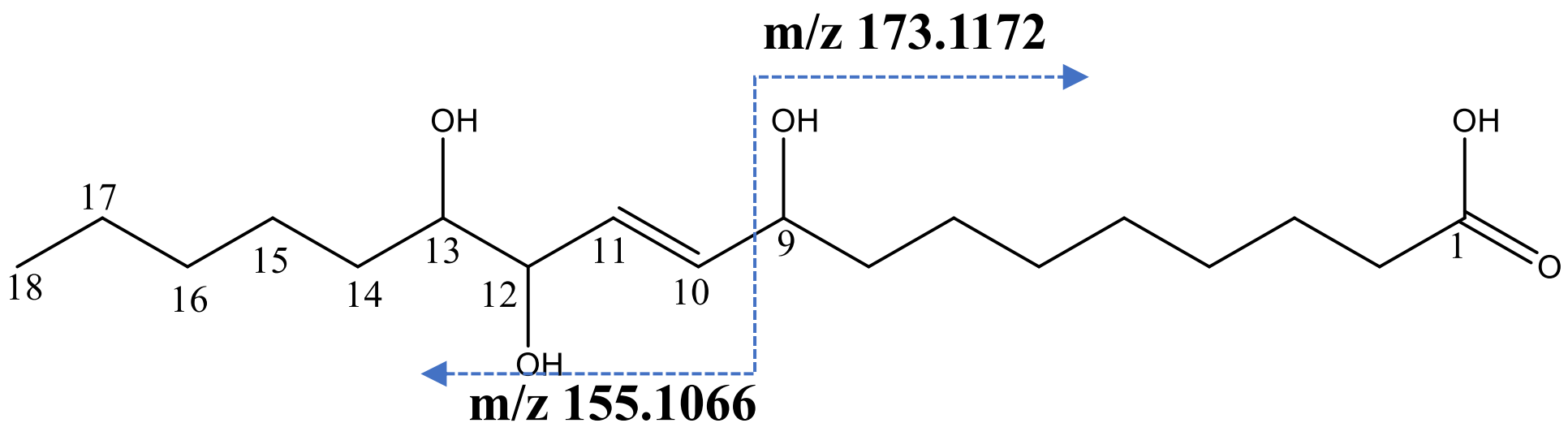

Scheme 1: mass fragmentation pattern of **3**

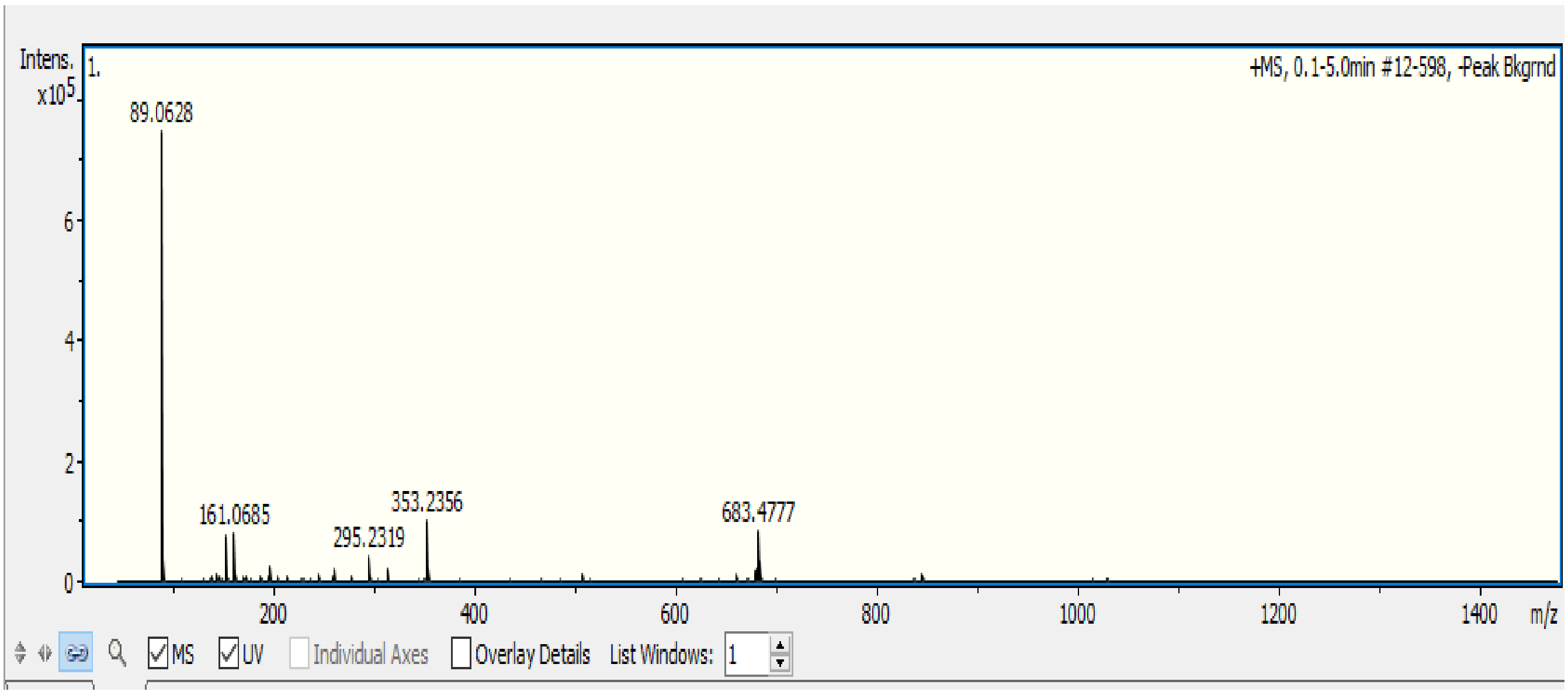

Figure S24: HR-ESI-MS spectrum of compound **4**

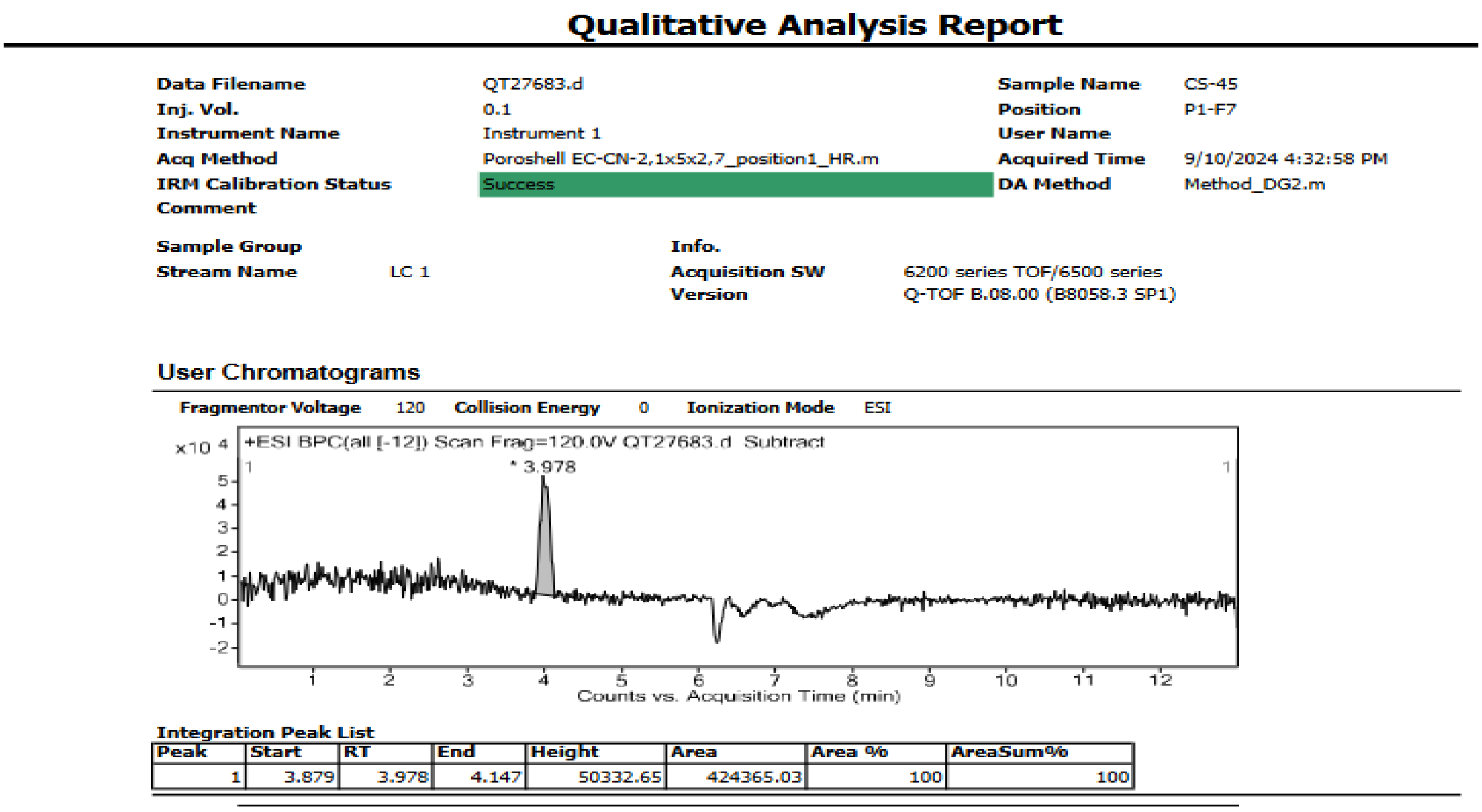

Qualitative Analysis Report

Data Filename	QT27683.d	Sample Name	CS-45
Inj. Vol.	0.1	Position	P1-F7
Instrument Name	Instrument 1	User Name	
Acq Method	Poroshell EC-CN-2,1x5x2,7_position1_HR.m	Acquired Time	9/10/2024 4:32:58 PM
IRM Calibration Status	Success	DA Method	Method_DG2.m
Comment			

Sample Group

Stream Name LC 1

Info.

Acquisition SW Version 6200 series TOF/6500 series Q-TOF B.08.00 (B8058.3 SP1)

User Chromatograms

Fragmentor Voltage 120 Collision Energy 0 Ionization Mode ESI

Integration Peak List

Peak	Start	RT	End	Height	Area	Area %	AreaSum%
1	3.879	3.978	4.147	50332.65	424365.03	100	100

Figure S25: LC-MS profile of Compound **4**

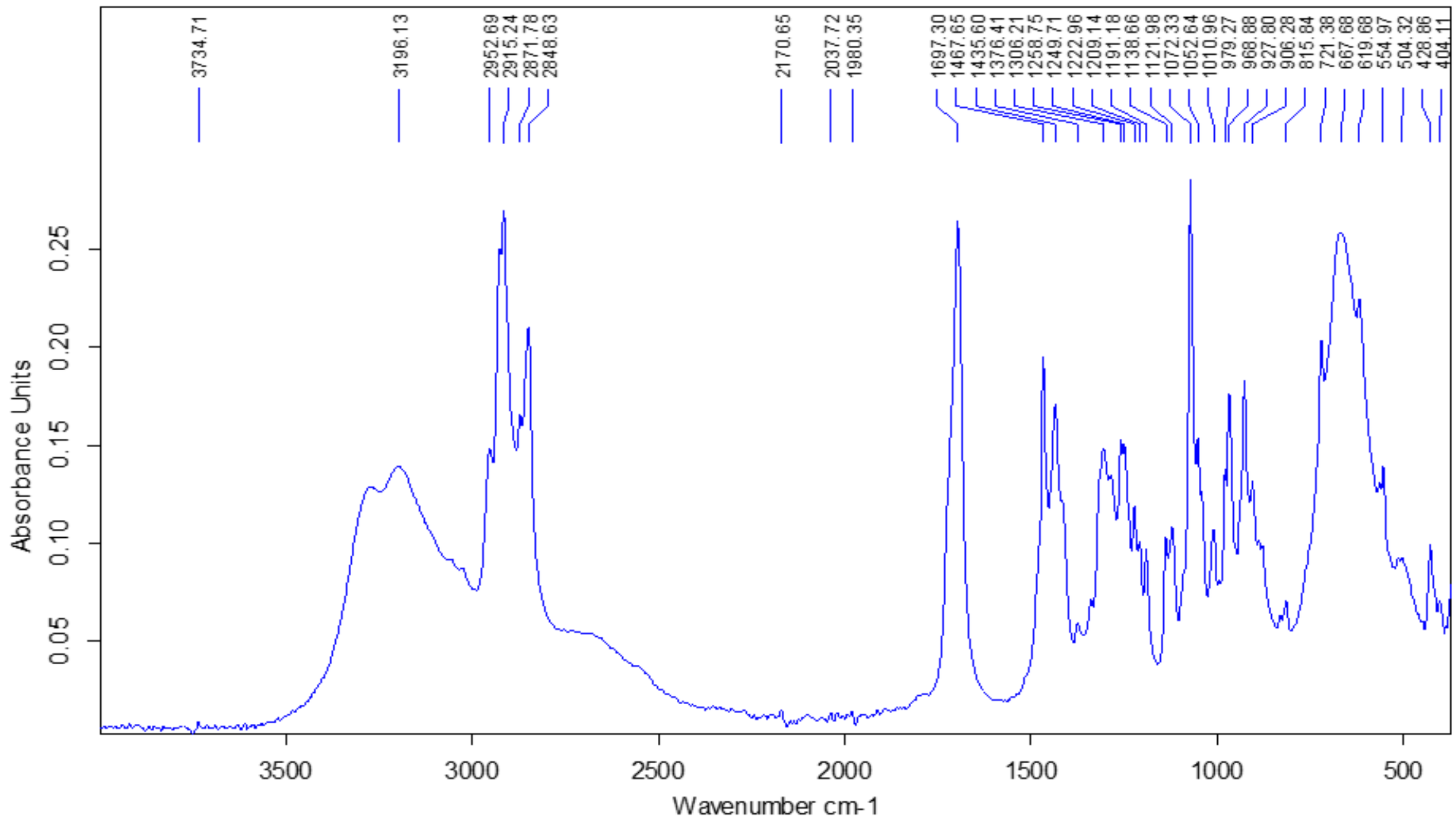

Figure S26: IR spectrum of compound **4**

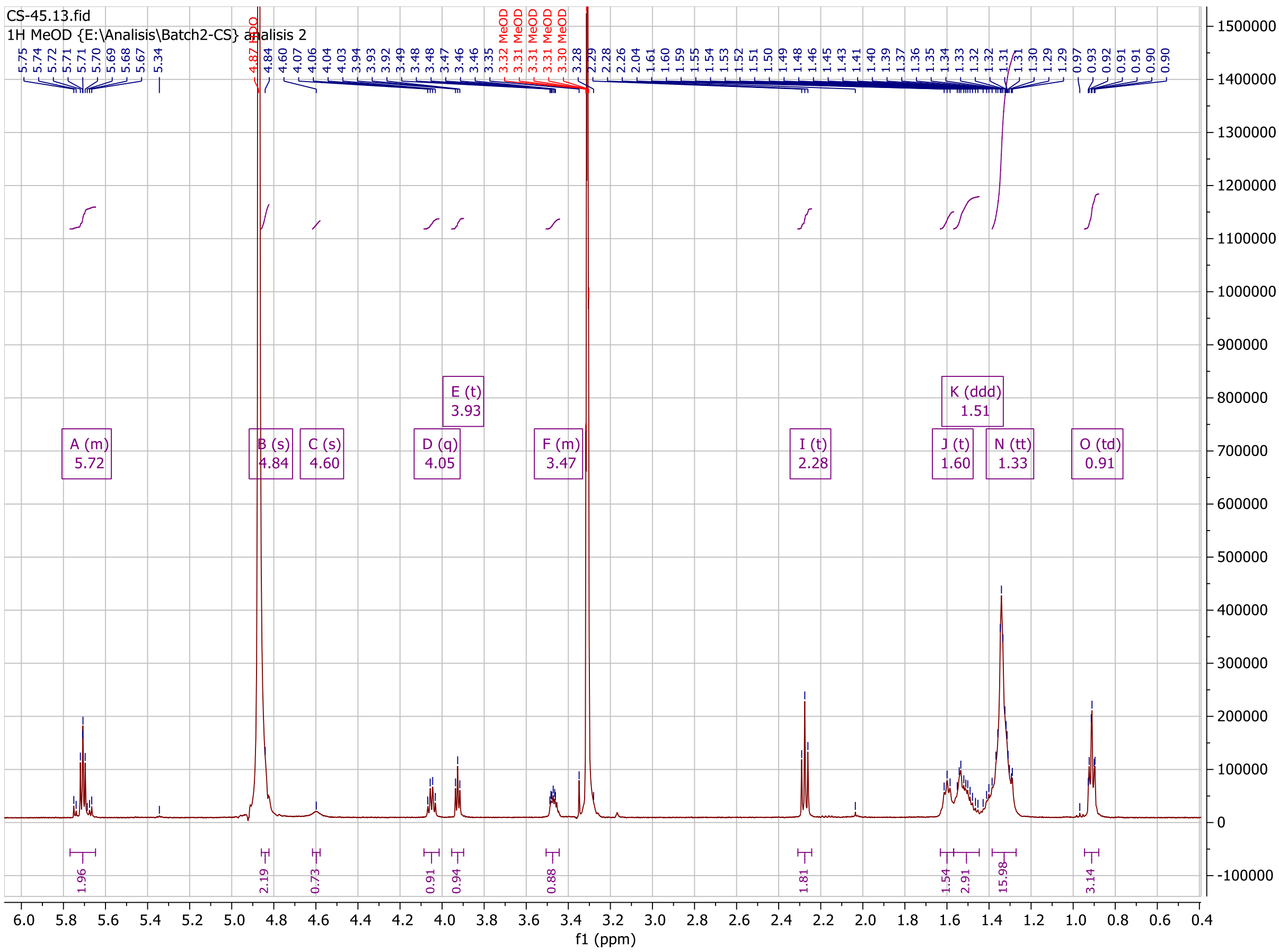

Figure S27: ^{1}H NMR (500 MHz, CD_3OD) spectrum of compound **4**

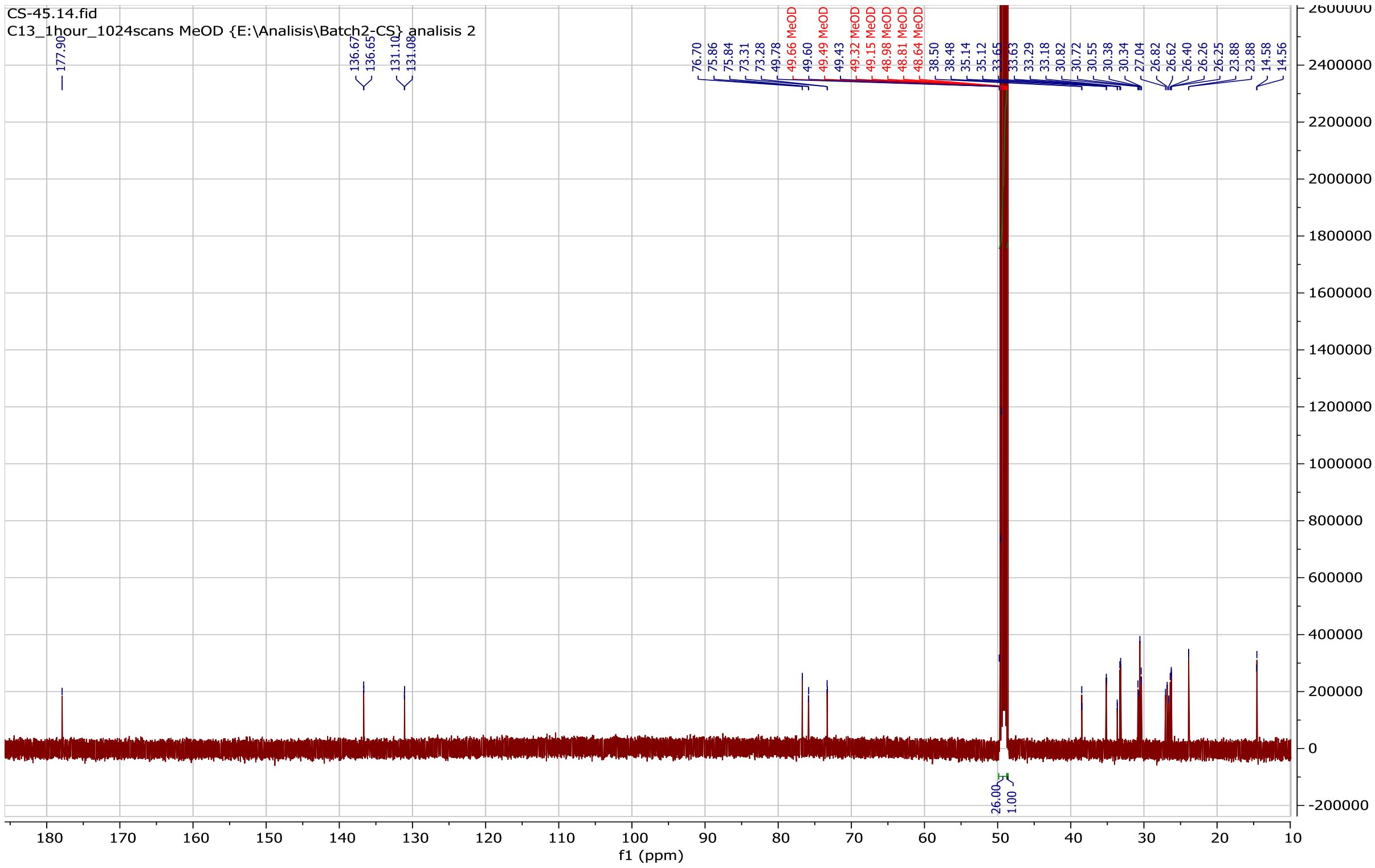

Figure S28: ^{13}C NMR (125 MHz, CD_3OD) spectrum of compound **4**

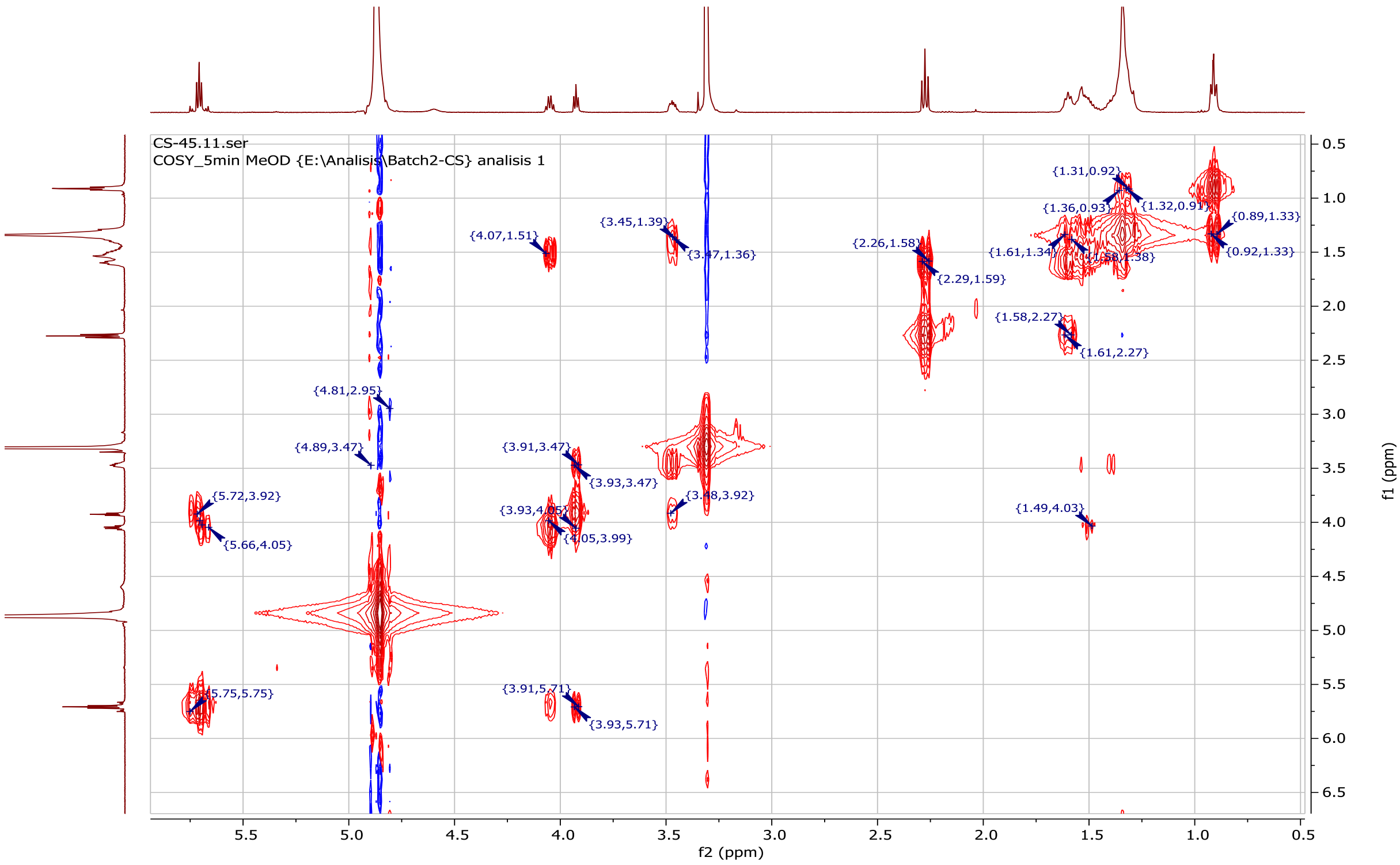

Figure S29: COSY spectrum of compound **4**

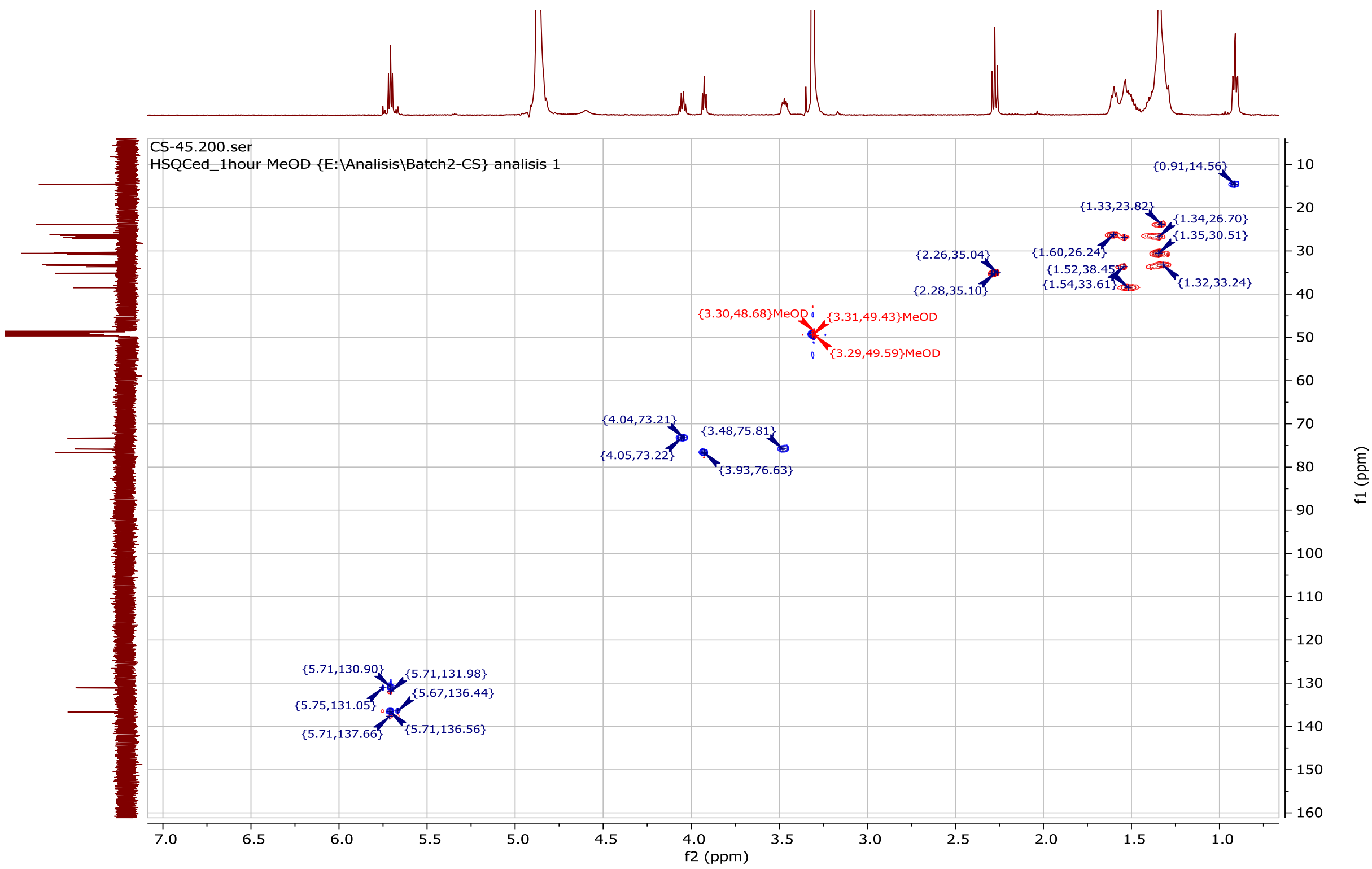

Figure S30: HSQC spectrum of compound **4**

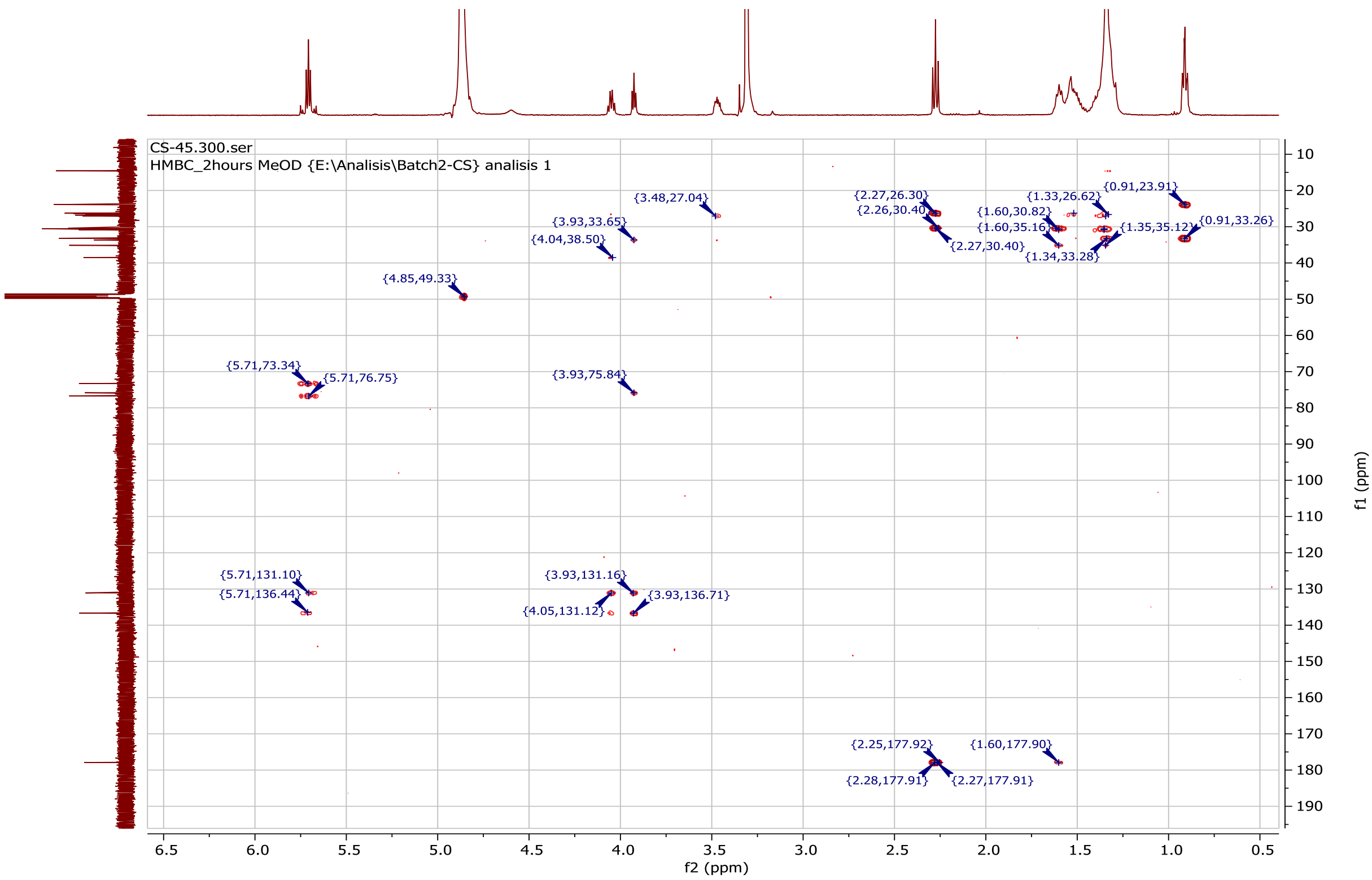

Figure S31: HMBC spectrum of compound **4**

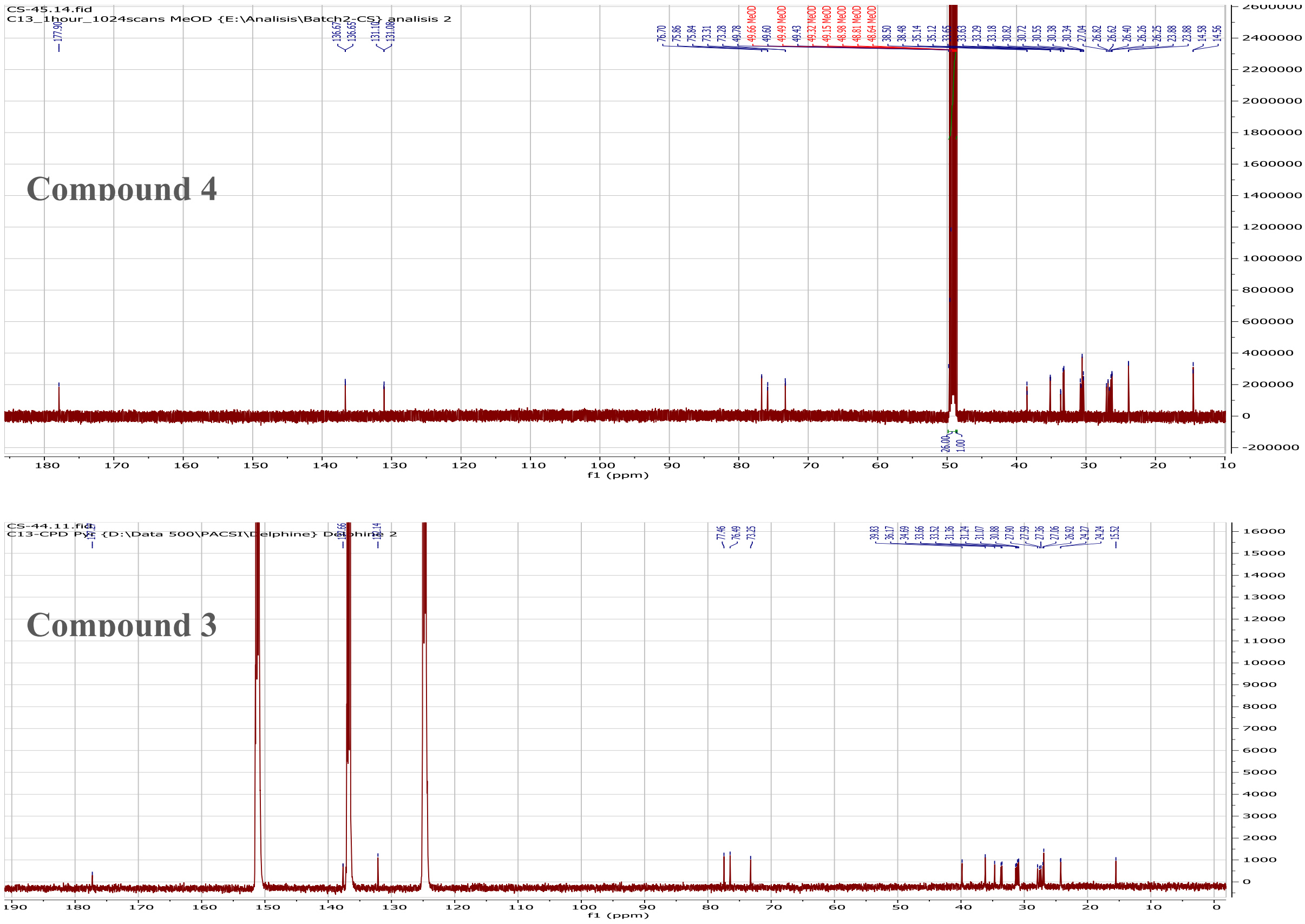

Figure S32: Compared ^{13}C NMR (125 MHz, CD_3OD) spectrum of compound **4** and **3** {125 MHz, $CDCl_3/CD_3OD$ (1:1)}

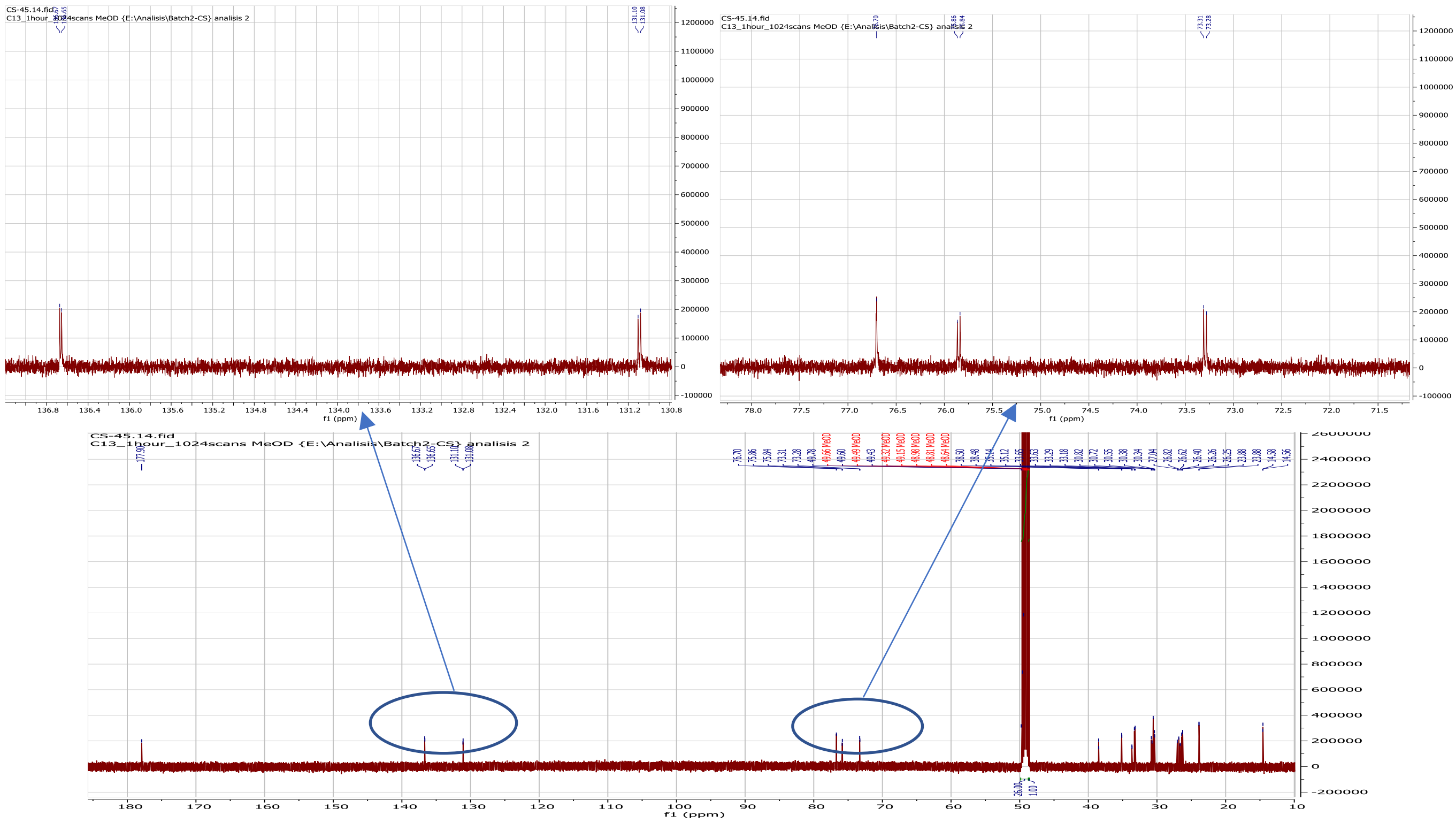

Figure S33: Extended ^{13}C NMR (125 MHz, CD_3OD) spectrum of compound **4**

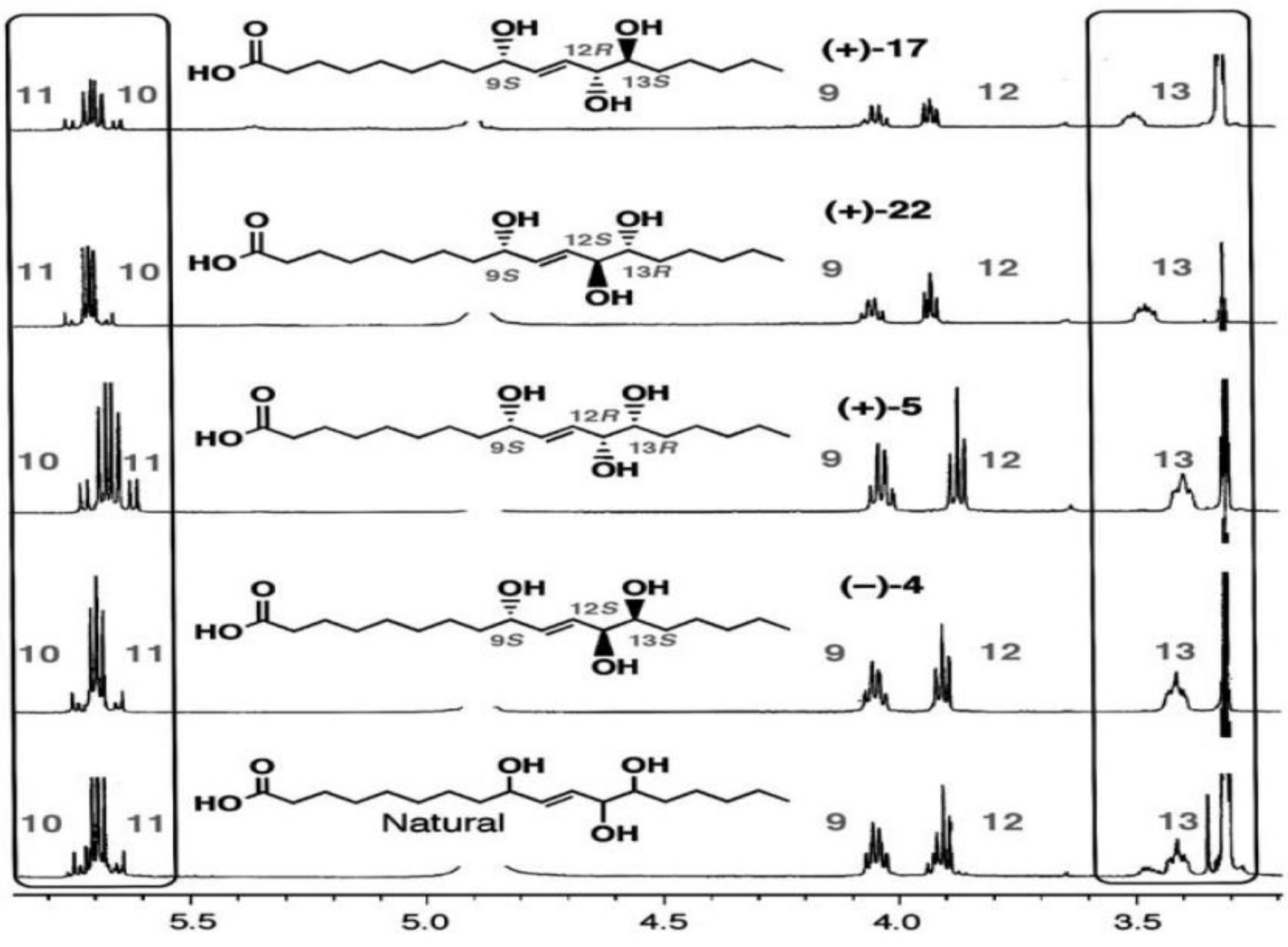

Figure S34: ^{1}H NMR spectra of stereoisomers of pinellic acid (Shirahata *et al*., 2006).

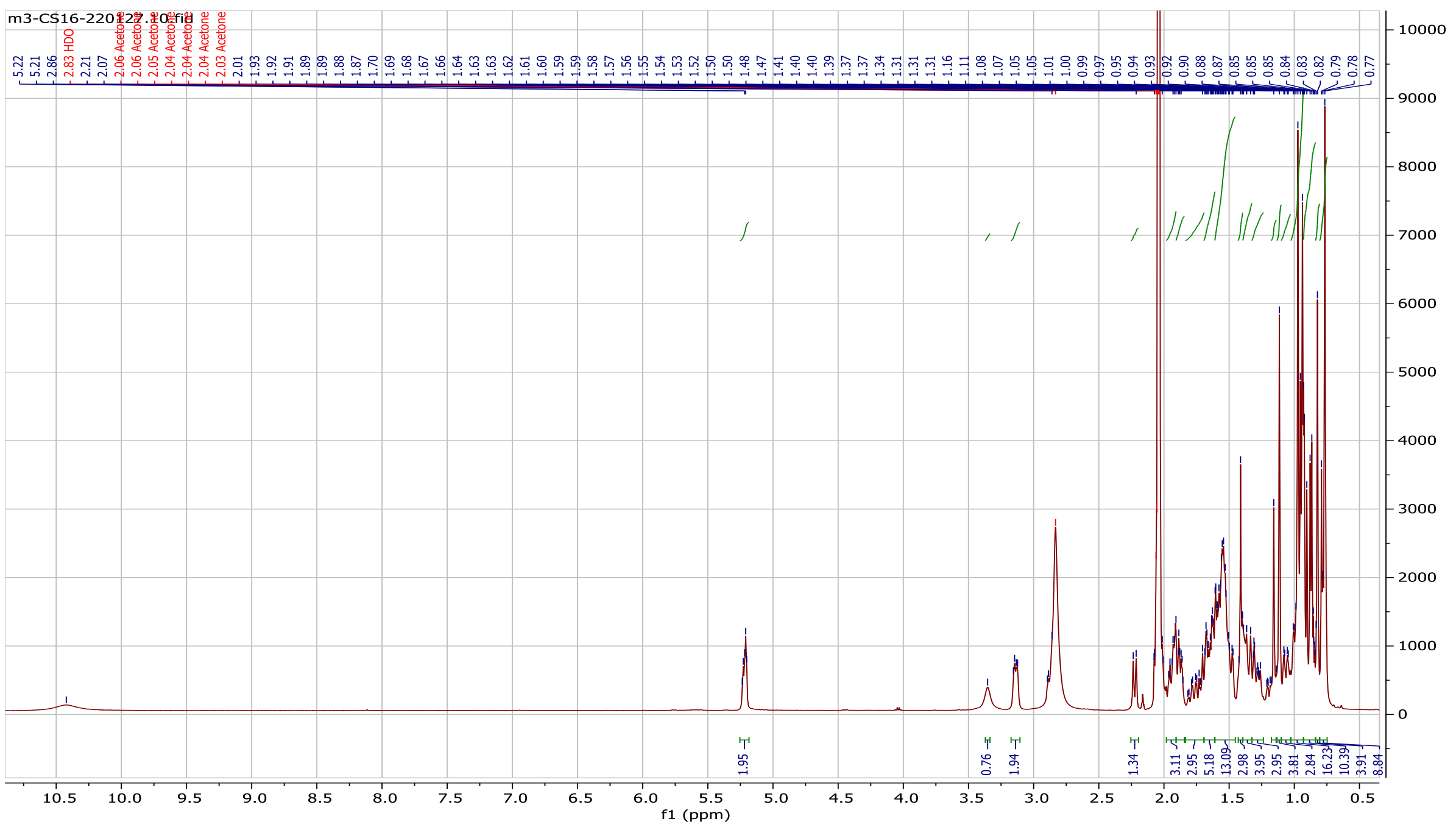

Figure S35: ^{1}H NMR spectrum (Acetone-d_6, 500 MHz) of the mixture of **5** and **6**

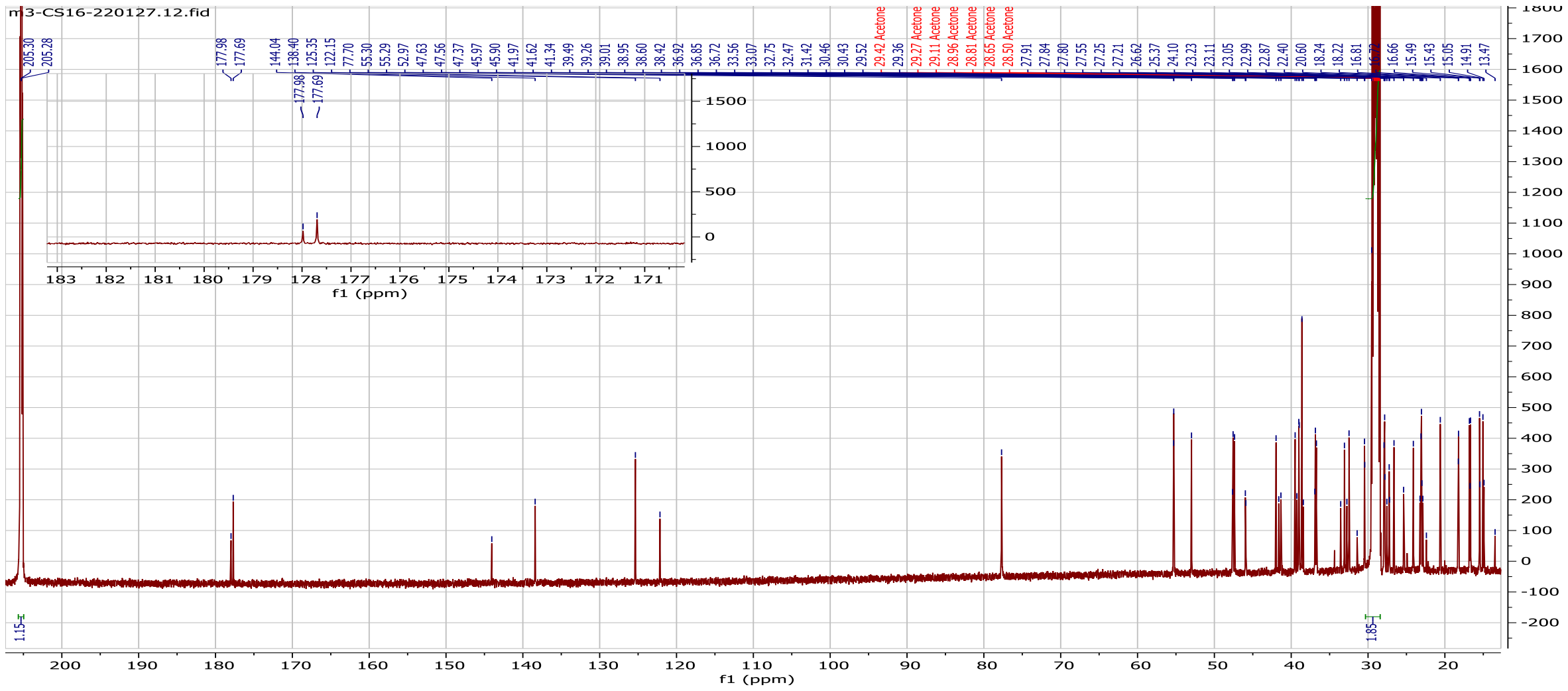

Figure S36: ^{13}C NMR spectrum (Acetone-d_6, 125 MHz) of the mixture of **5** and **6**

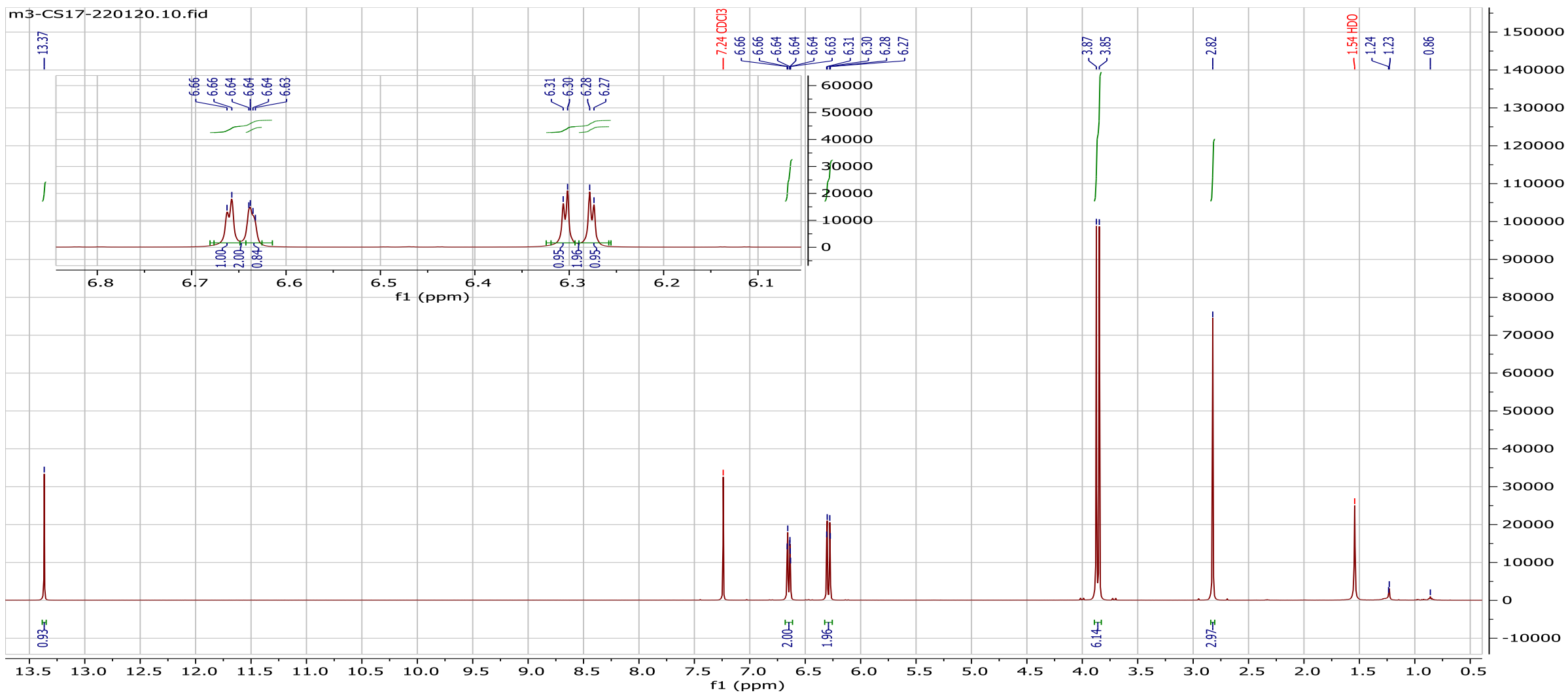

Figure S37: ^{1}H NMR spectrum ($CDCl_3$, 500 MHz) of **7**

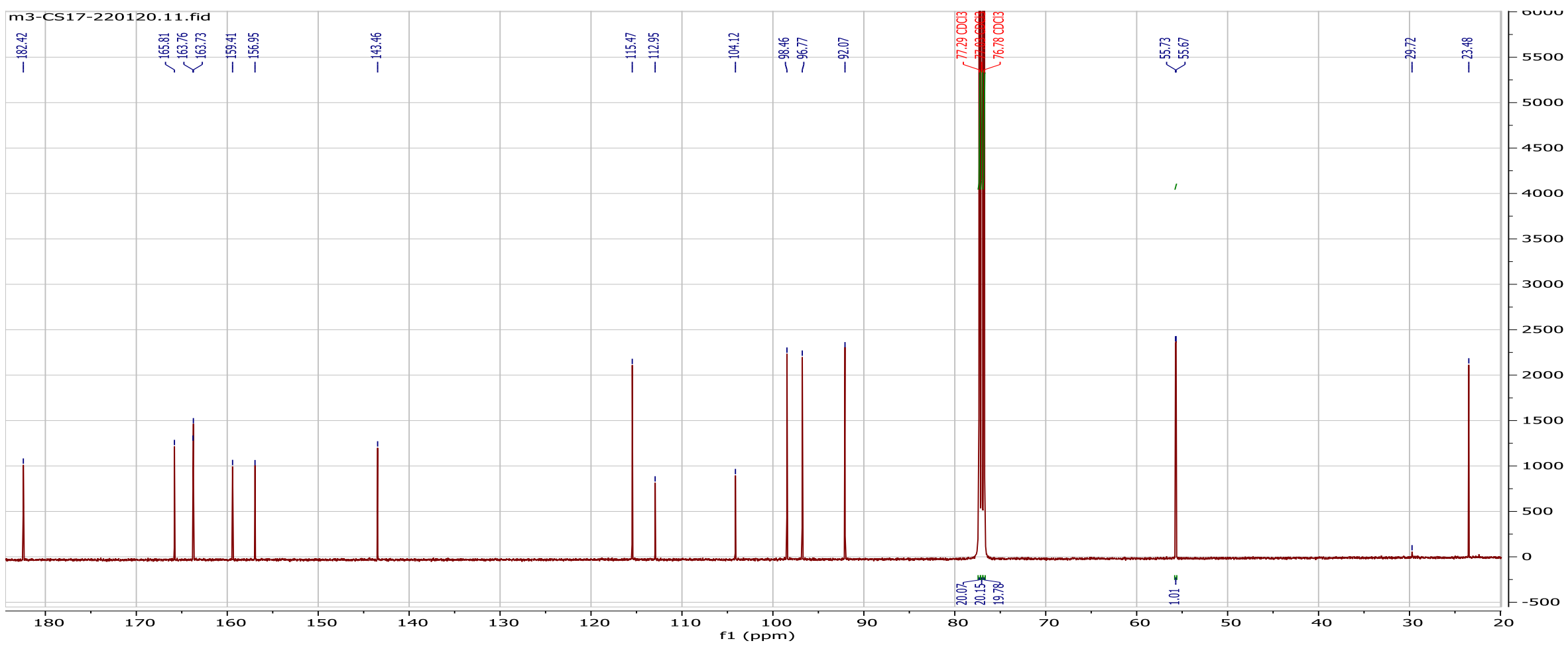

Figure S38: ^{13}C NMR spectrum ($CDCl_3$, 125 MHz) of **7**

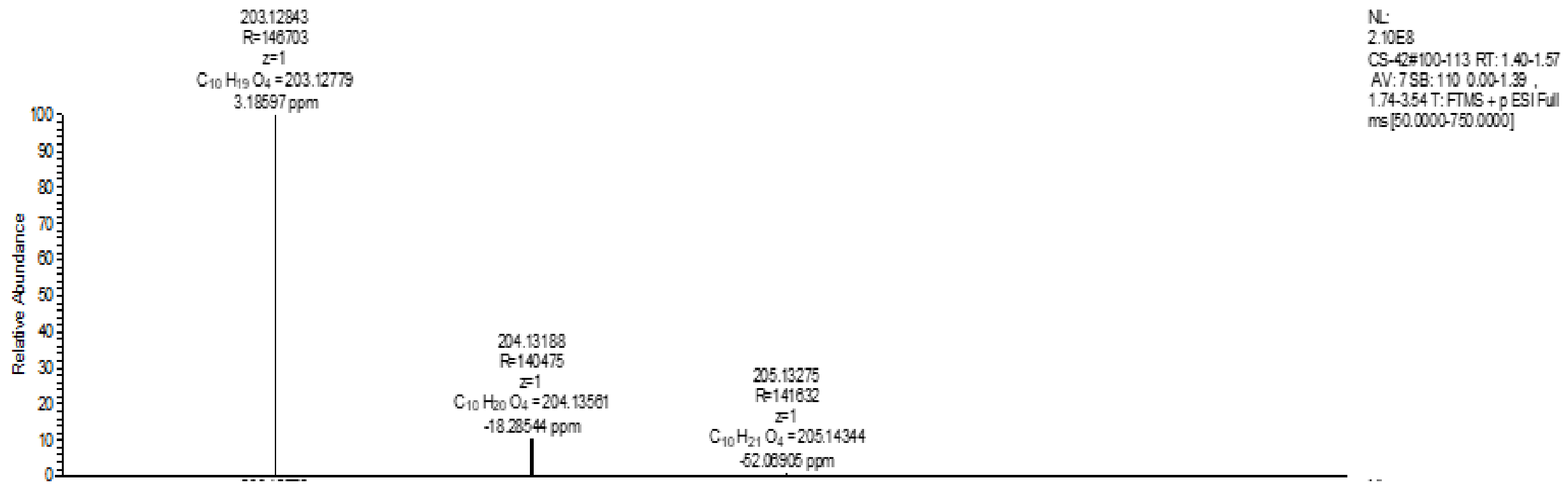

Figure S39: HR-ESI-MS of **8**

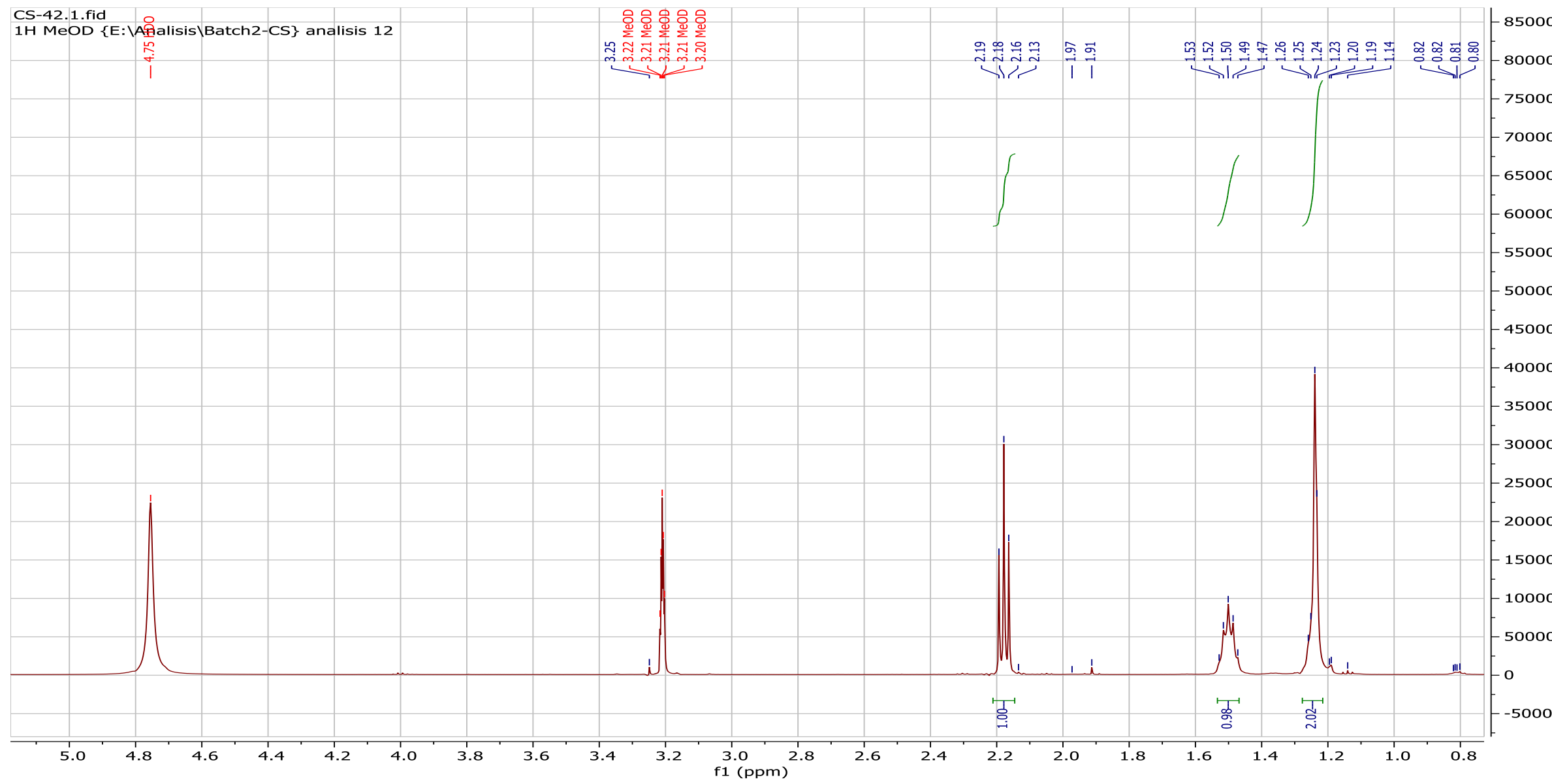

Figure S40: ^{1}H NMR spectrum (CD_3OD, 500 MHz) of **8**

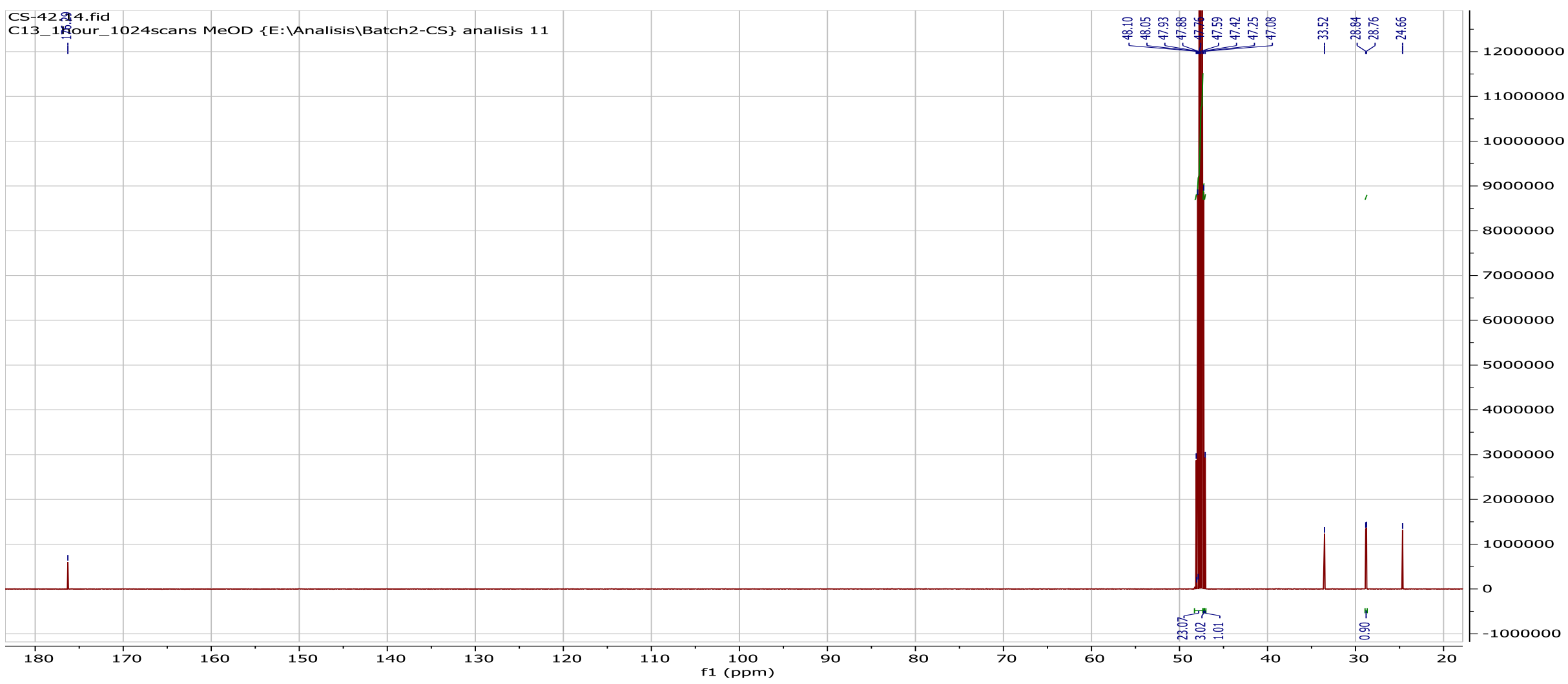

Figure S41: ^{13}C NMR spectrum (CD_3OD, 125 MHz) of **8**

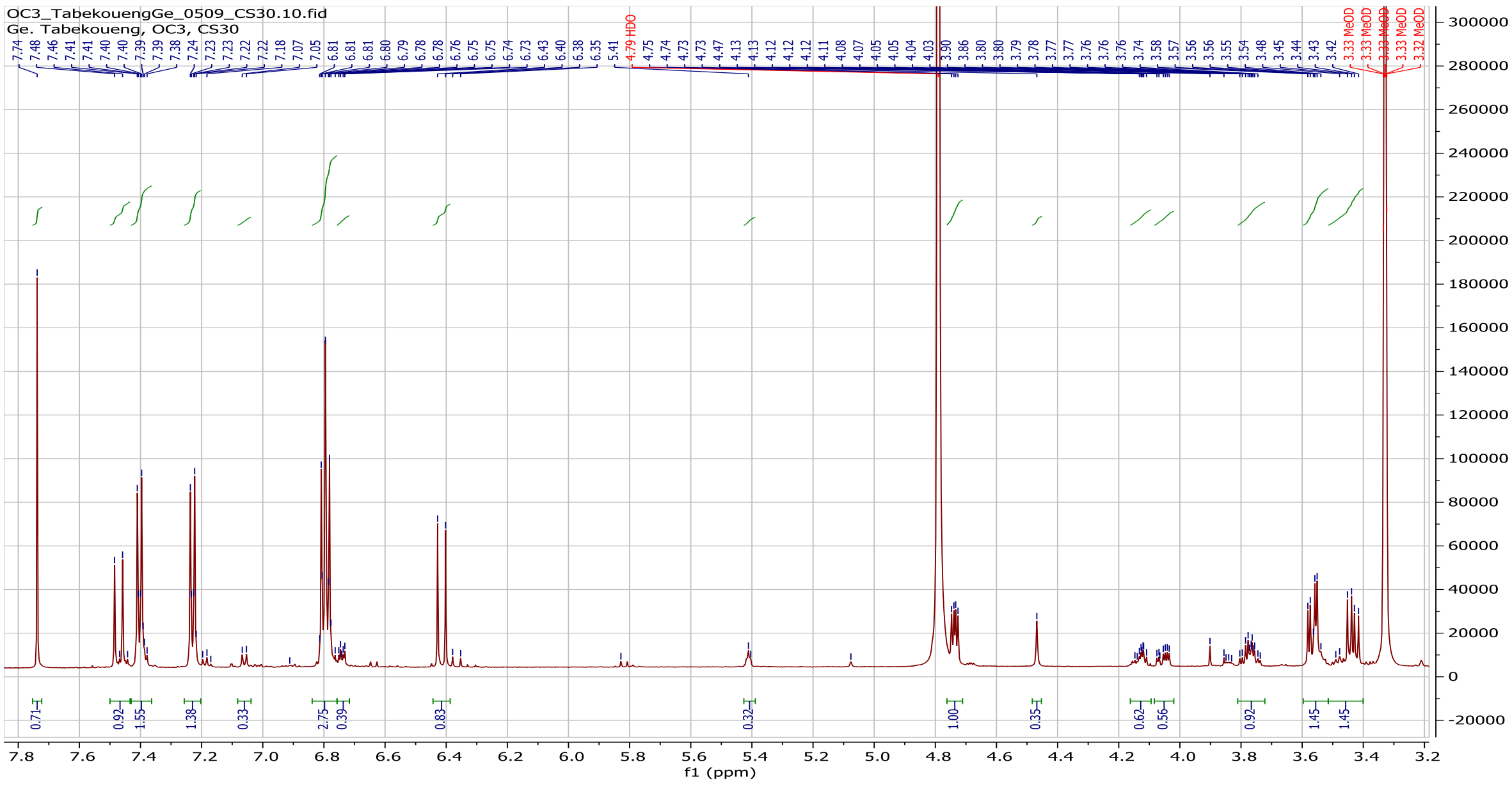

Figure S42: ^{1}H NMR spectrum ($CDCl_3$/CD_3OD, 600 MHz) of **9**

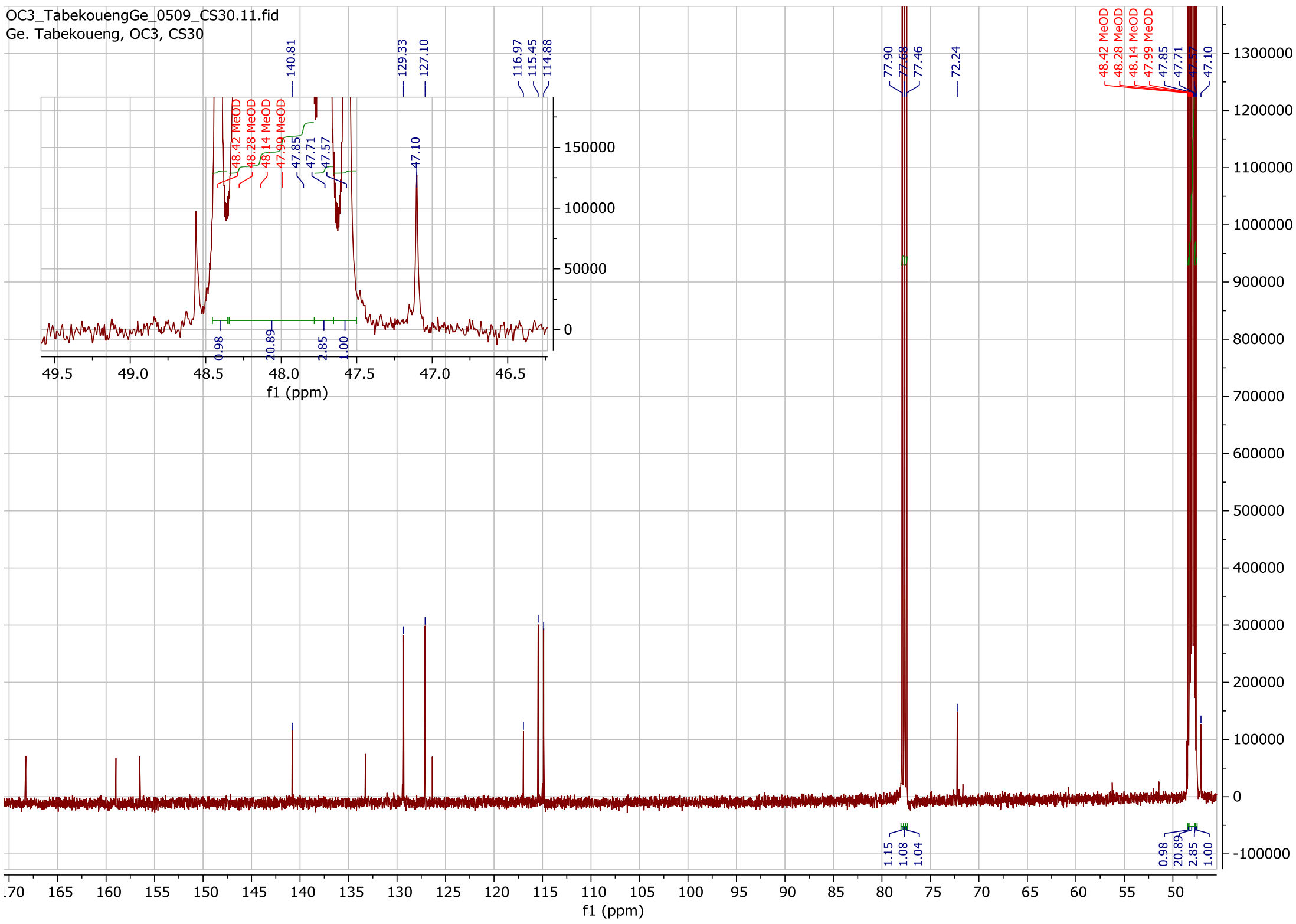

Figure S43: ^{13}C NMR spectrum ($CDCl_3$/CD_3OD, 150 MHz) of **9**

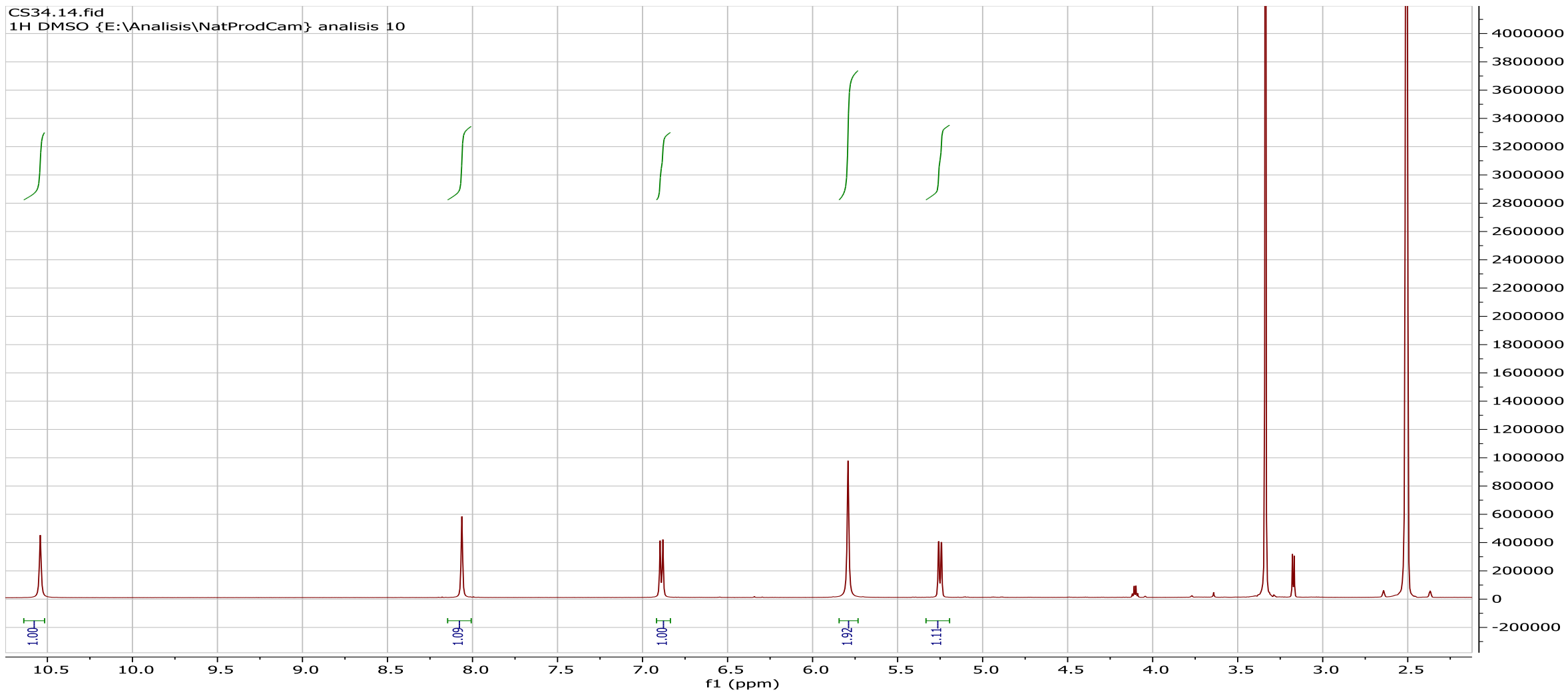

Figure S44: ^{1}H NMR spectrum (DMSO-d_6, 500 MHz) of **10**

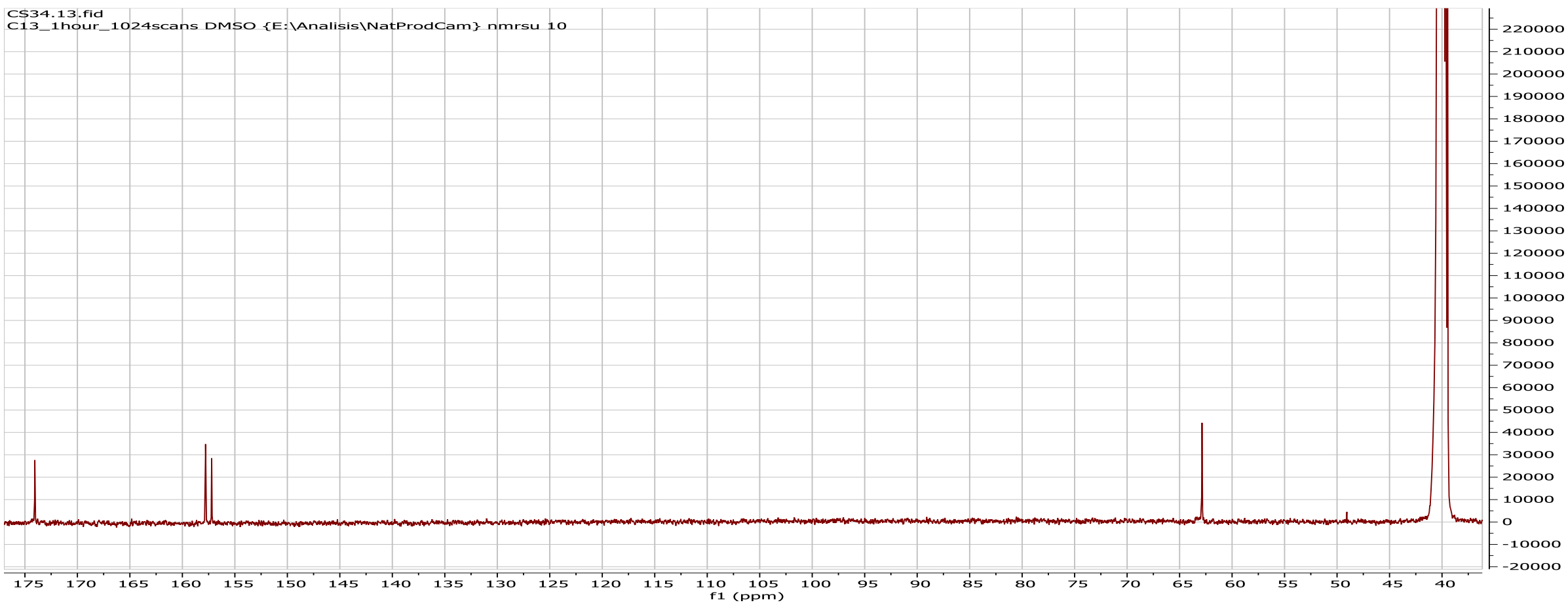

Figure S45: ^{13}C NMR spectrum (DMSO-d_6, 125 MHz) of **10**

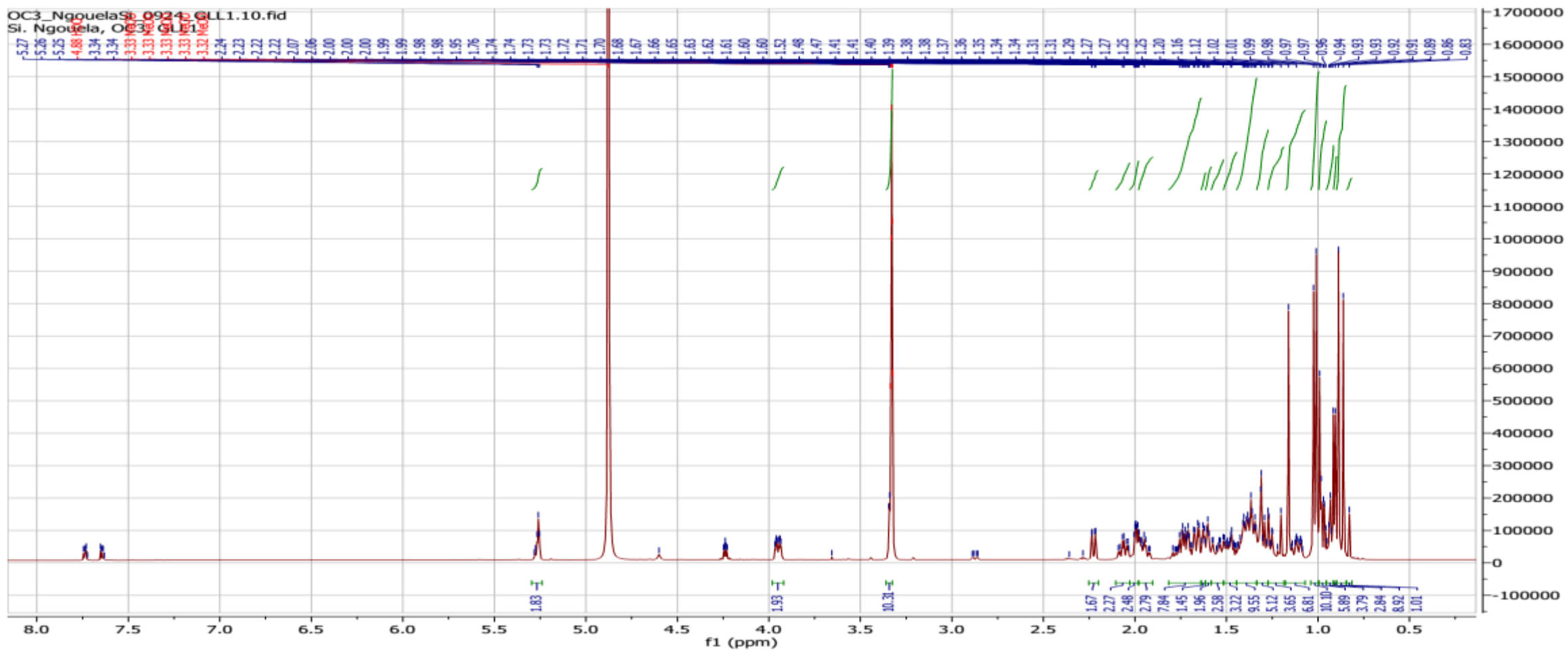

Figure S46: ^{1}H NMR spectrum of **11** (500 MHz, DMSO-d_6)

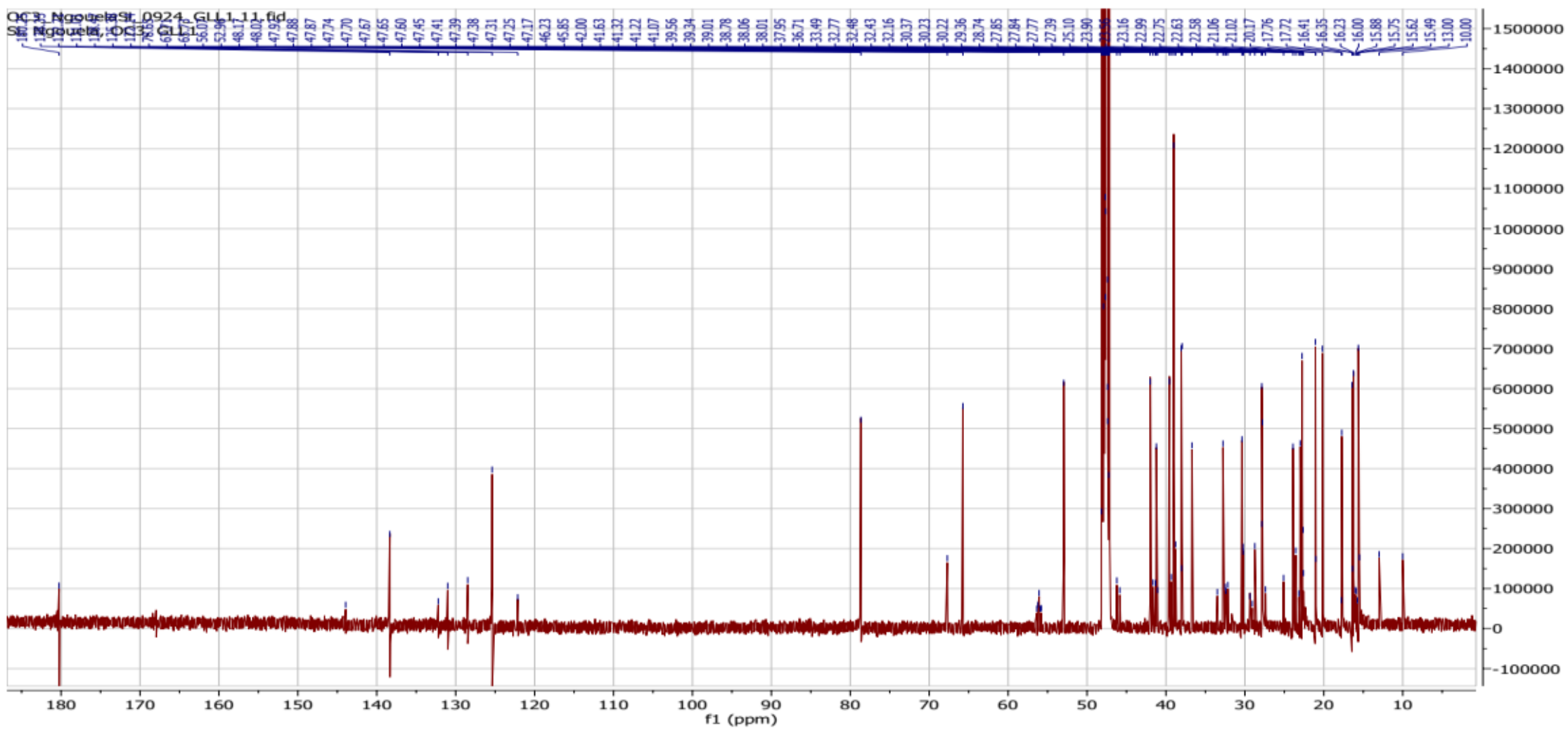

Figure S47: ^{13}C NMR spectrum of **11** (125 MHz, DMSO-d_6)

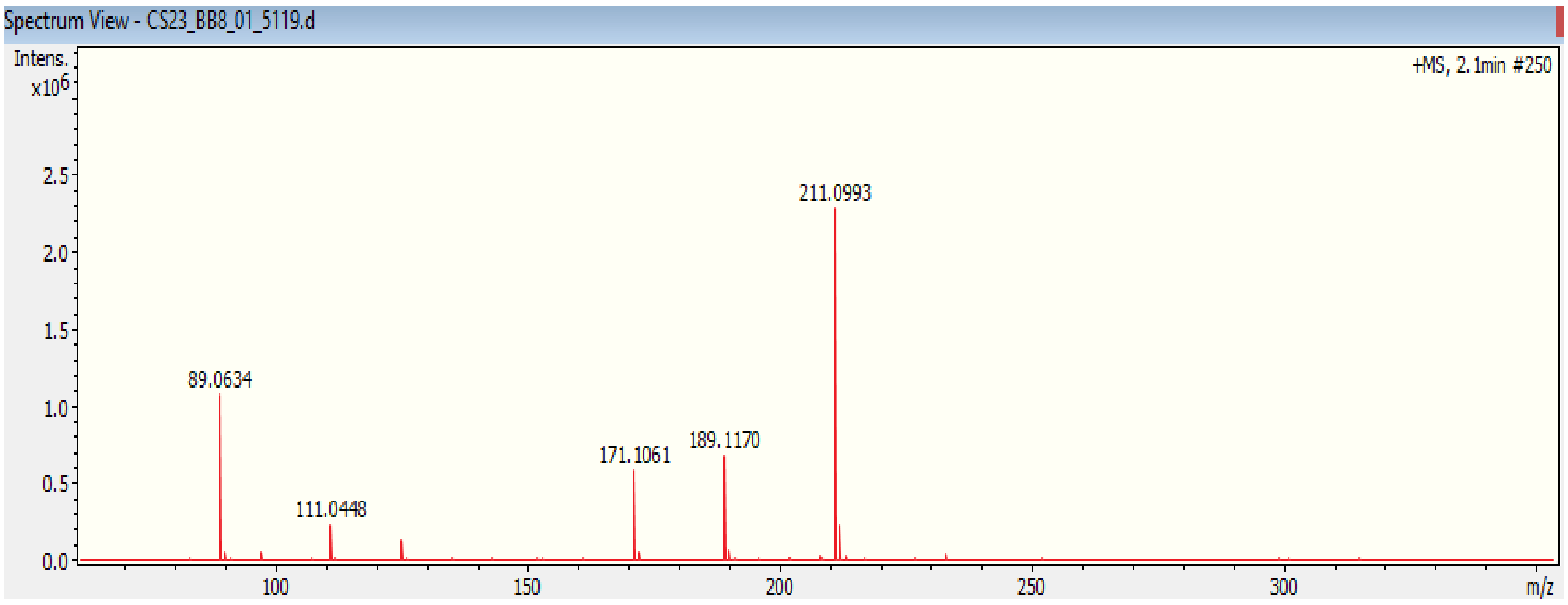

Figure S48: HR-ESI-MS of **12**

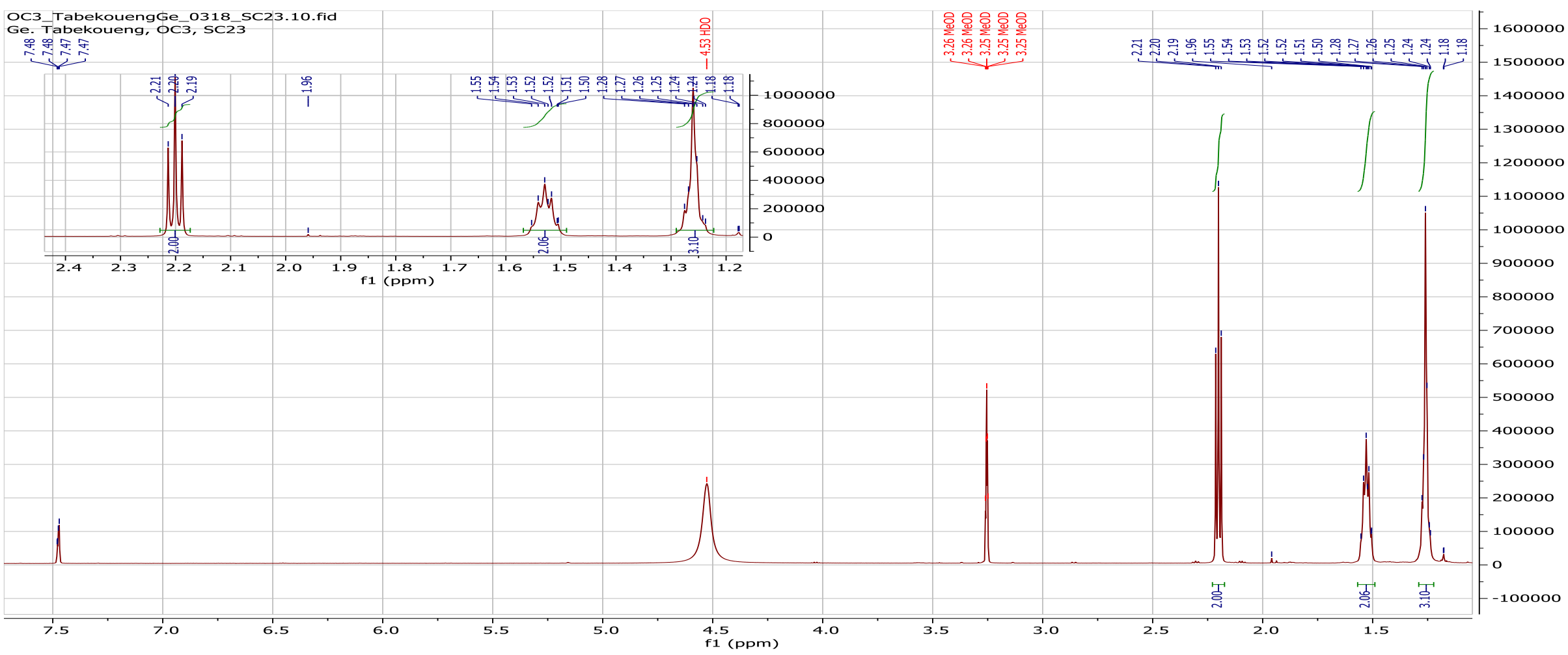

Figure S49: ^{1}H NMR spectrum ($CDCl_3$/CD_3OD, 600 MHz) of **12**

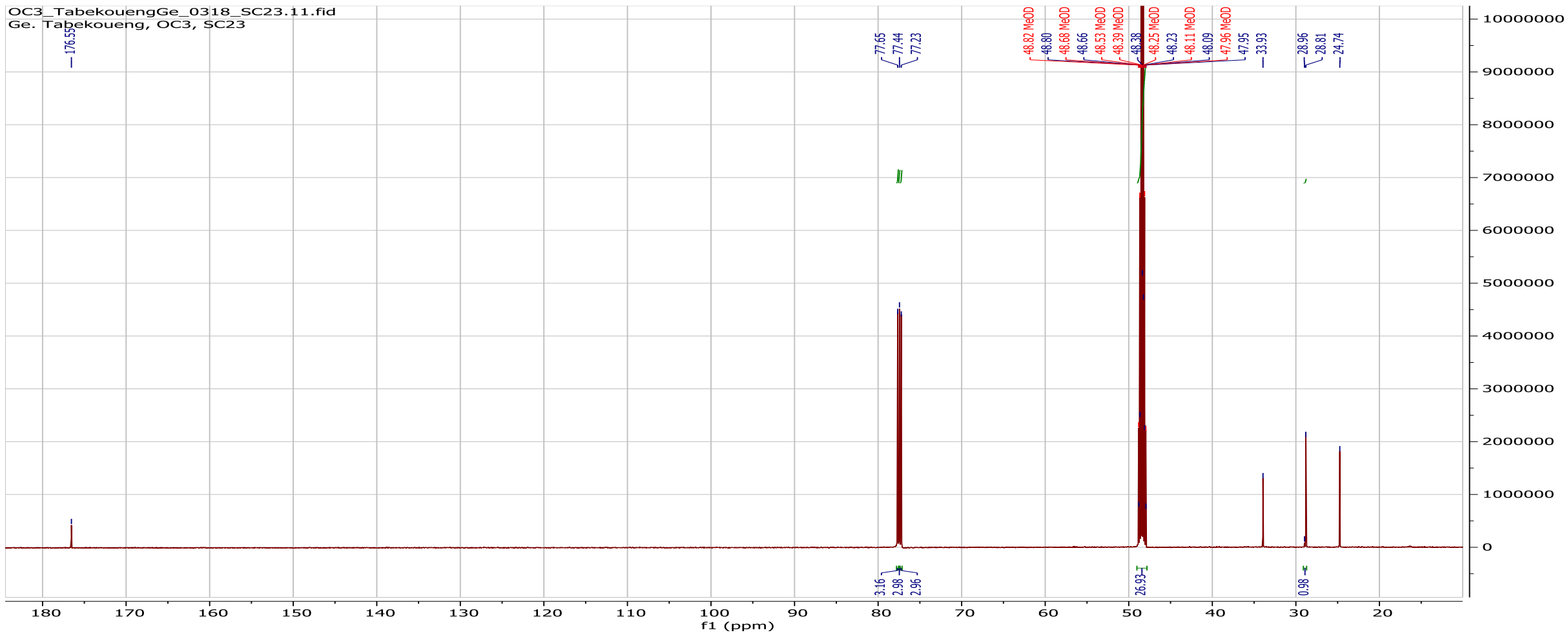

Figure S50: ^{13}C NMR spectrum {150 MHz, $CDCl_3/CD_3OD$ (1:1)} of **12**

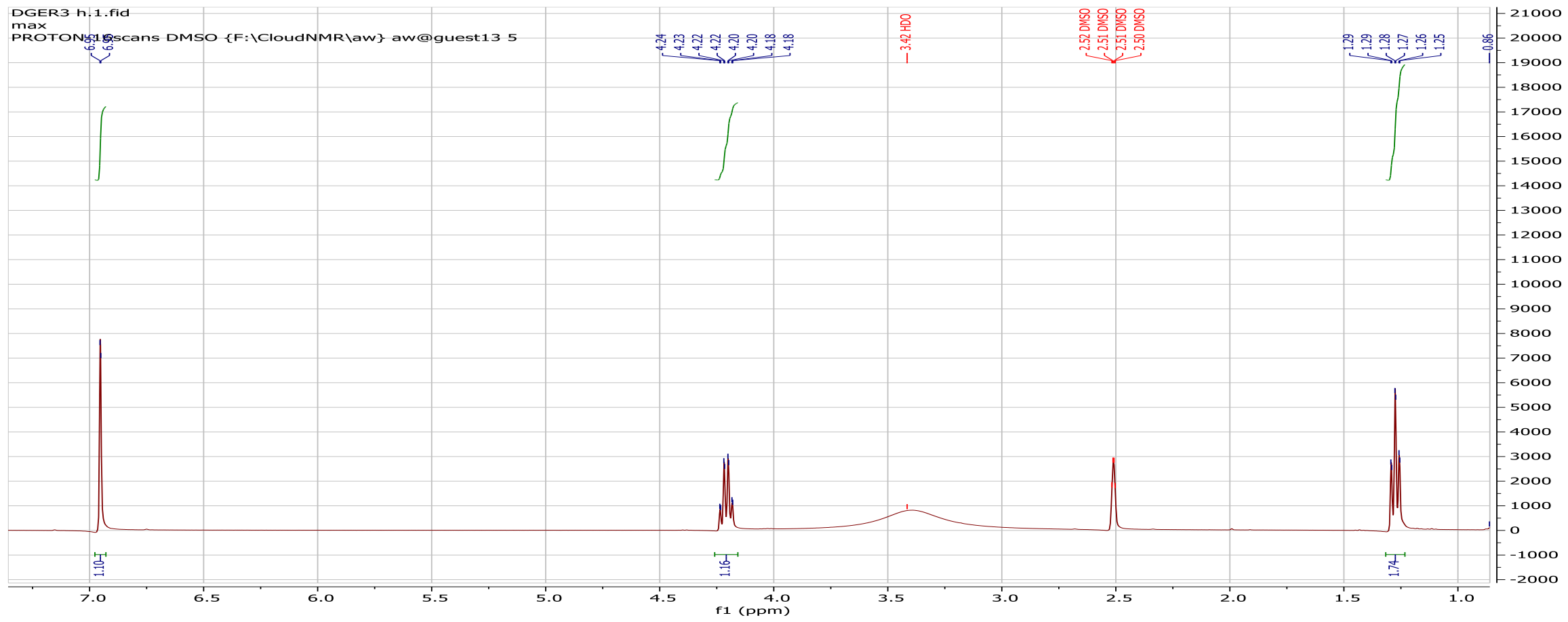

Figure S51: ^{1}H NMR spectrum (DMSO-d_6, 500 MHz) of **13**

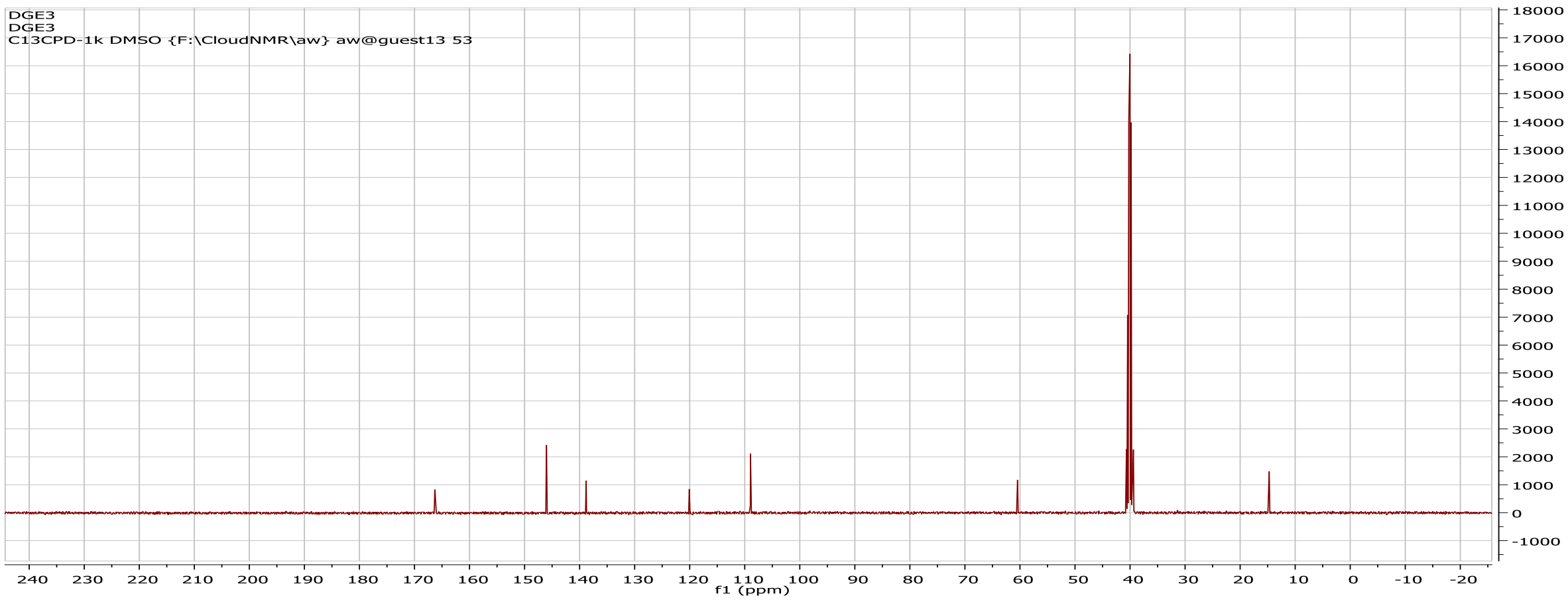

Figure S52: ^{13}C NMR spectrum (DMSO-d_6, 125 MHz) of **13**

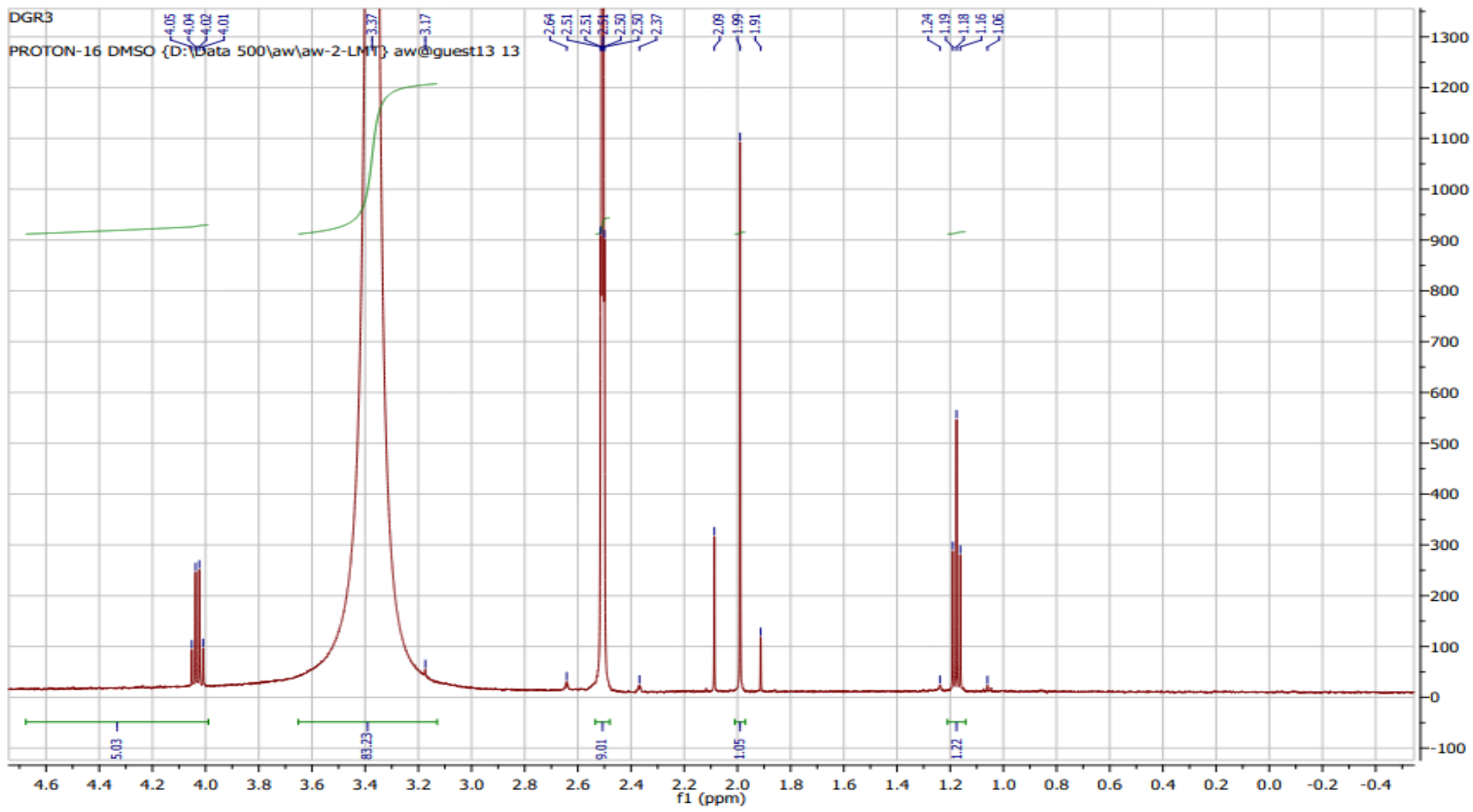

Figure S53: ^{1}H NMR spectrum (DMSO-d_6, 500 MHz) of **14**

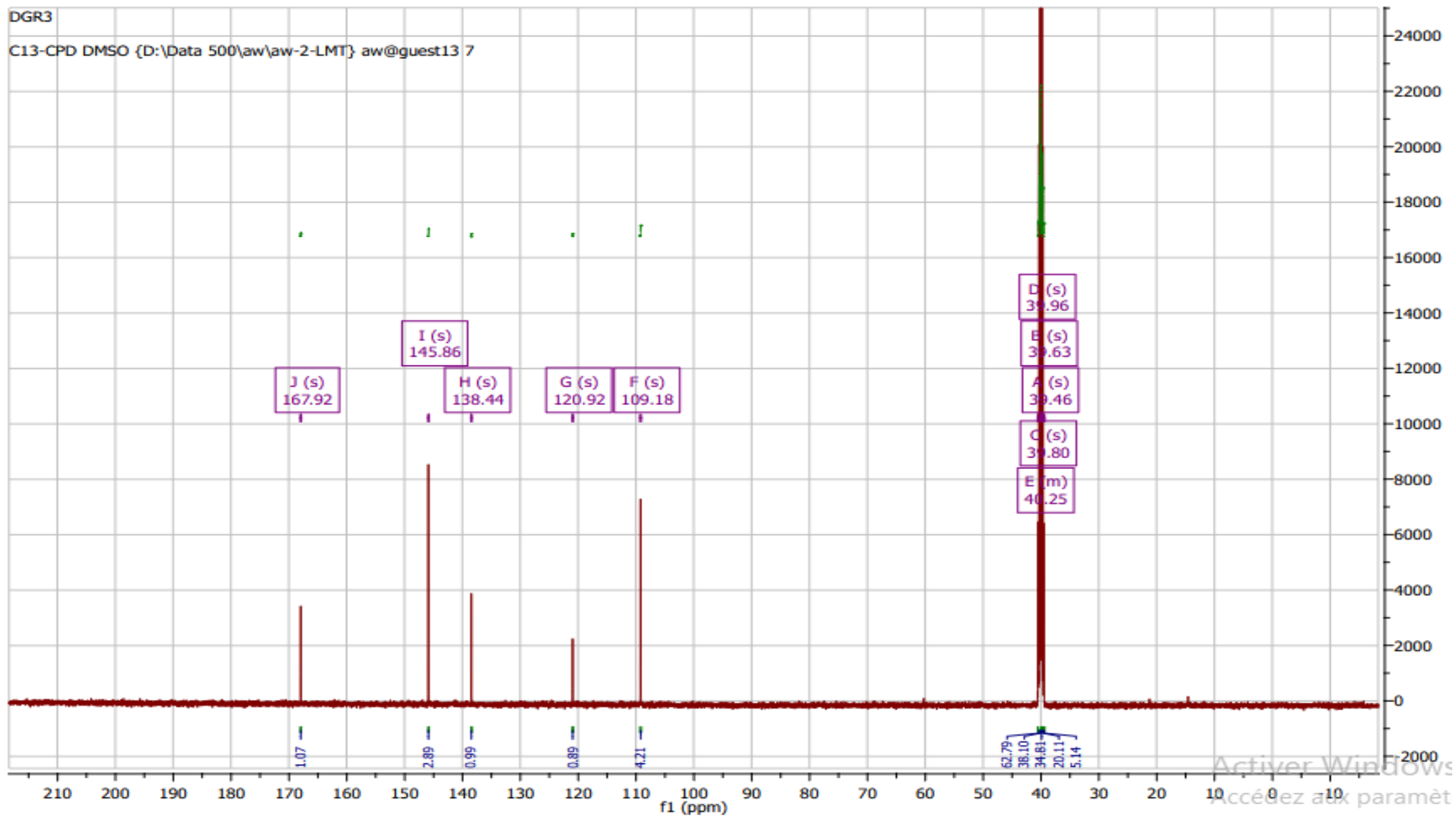

Figure S54: ^{13}C NMR spectrum (DMSO-d_6, 125 MHz) of **14**

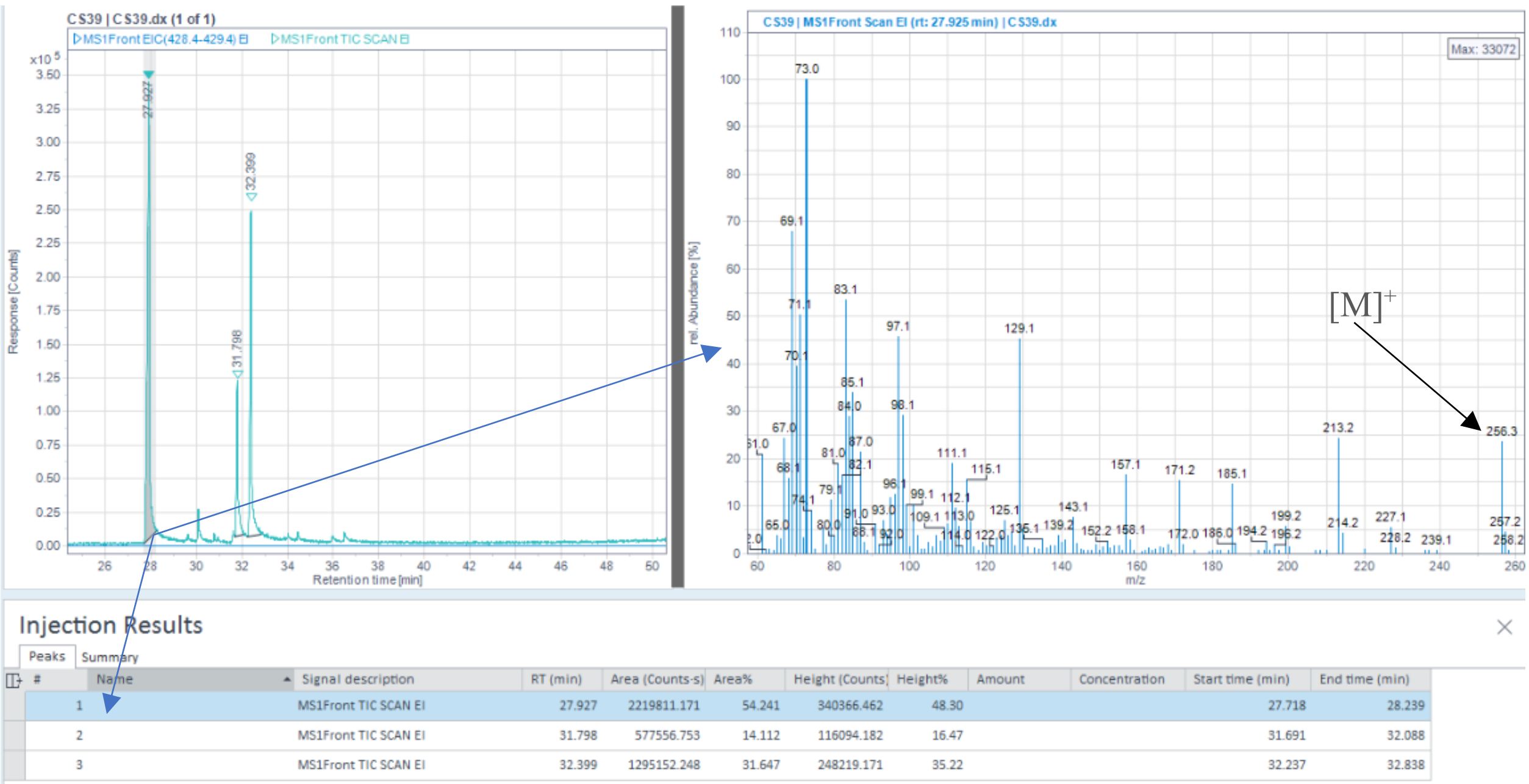

Figure S55: GC/EIMS pic 1 of **15**

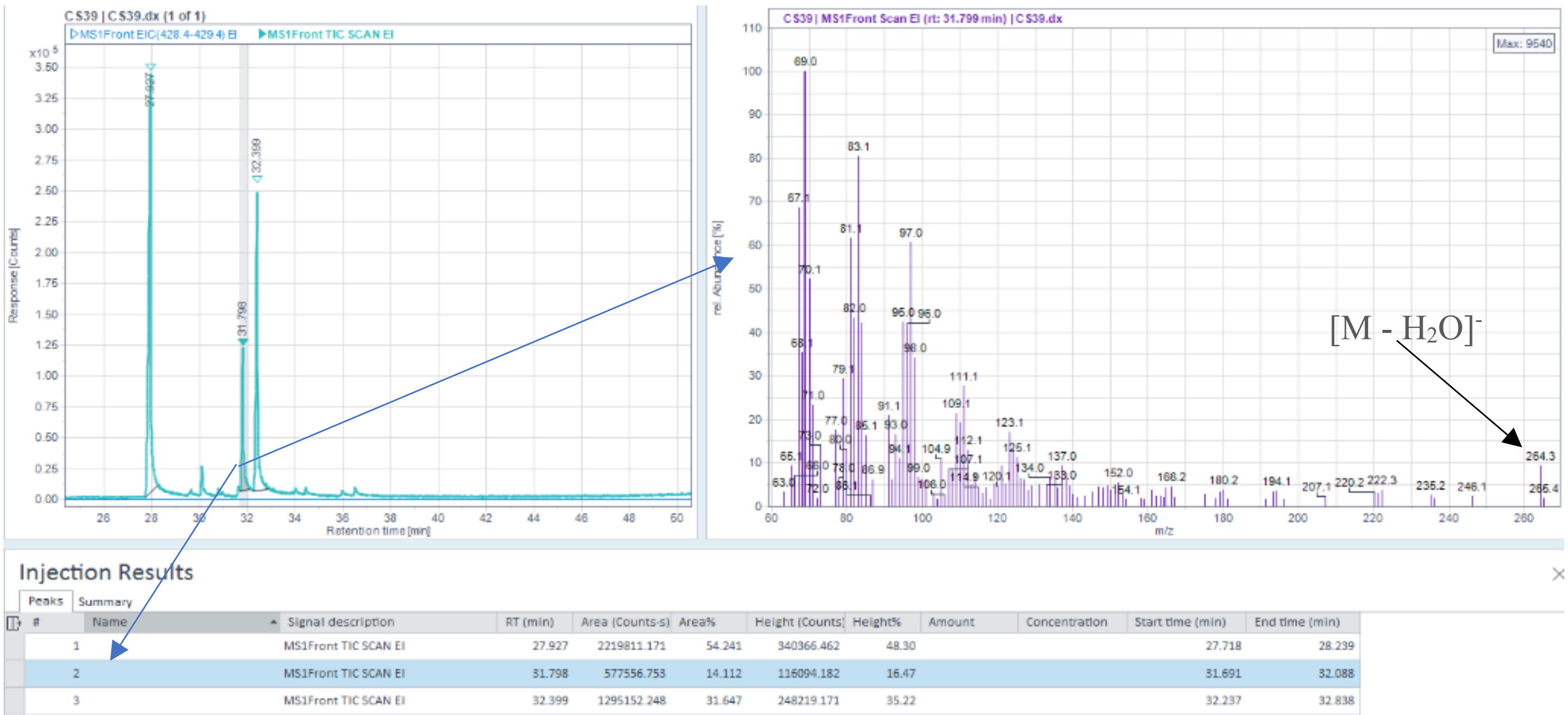

Figure S56: GC/EIMS pic 2 of **16**

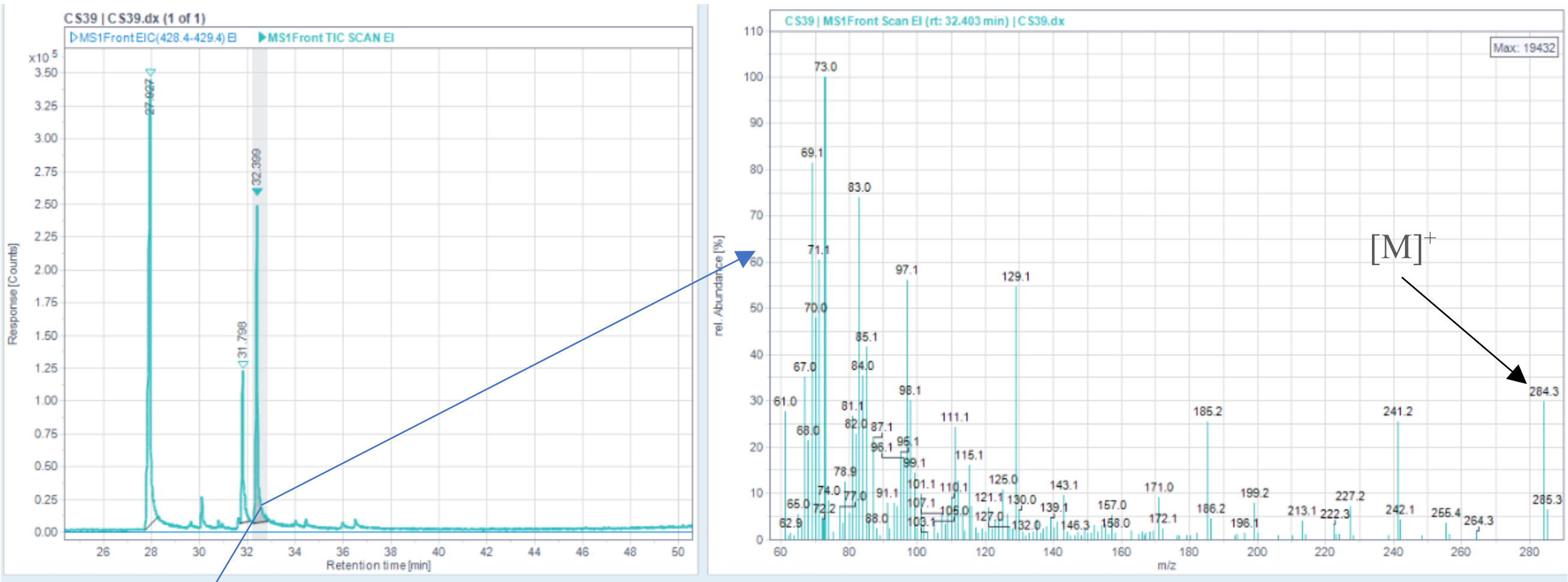

Injection Results

Peaks Summary

#	Name	Signal description	RT (min)	Area (Counts·s)	Area%	Height (Counts)	Height%	Amount	Concentration	Start time (min)	End time (min)
1		MS1Front TIC SCAN EI	27.927	2219811.171	54.241	340366.462	48.30			27.718	28.239
2		MS1Front TIC SCAN EI	31.798	577556.753	14.112	116094.182	16.47			31.691	32.088
3		MS1Front TIC SCAN EI	32.399	1295152.248	31.647	248219.171	35.22			32.237	32.838

Figure S57: GC/EIMS pic 3 of **17**

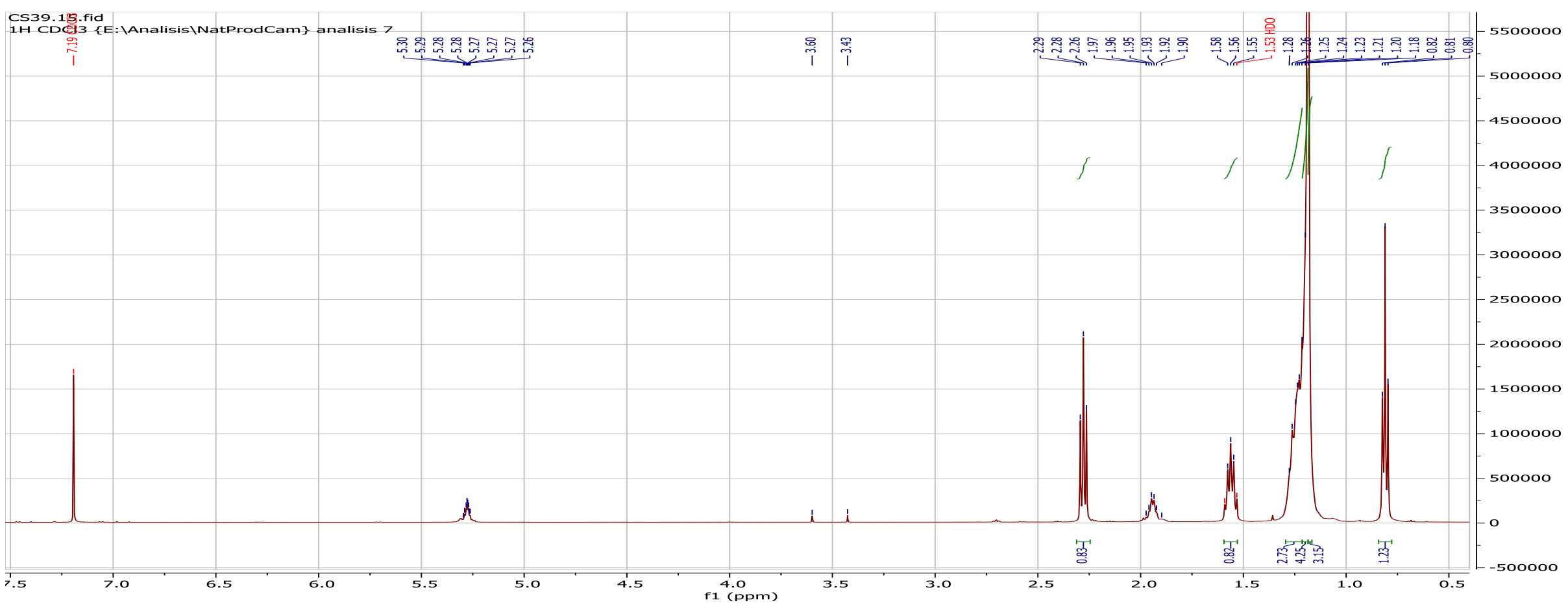

Figure S58: ^{1}H NMR spectrum ($CDCl_3$, 500 MHz) of the mixture of **15**, **16** and **17**

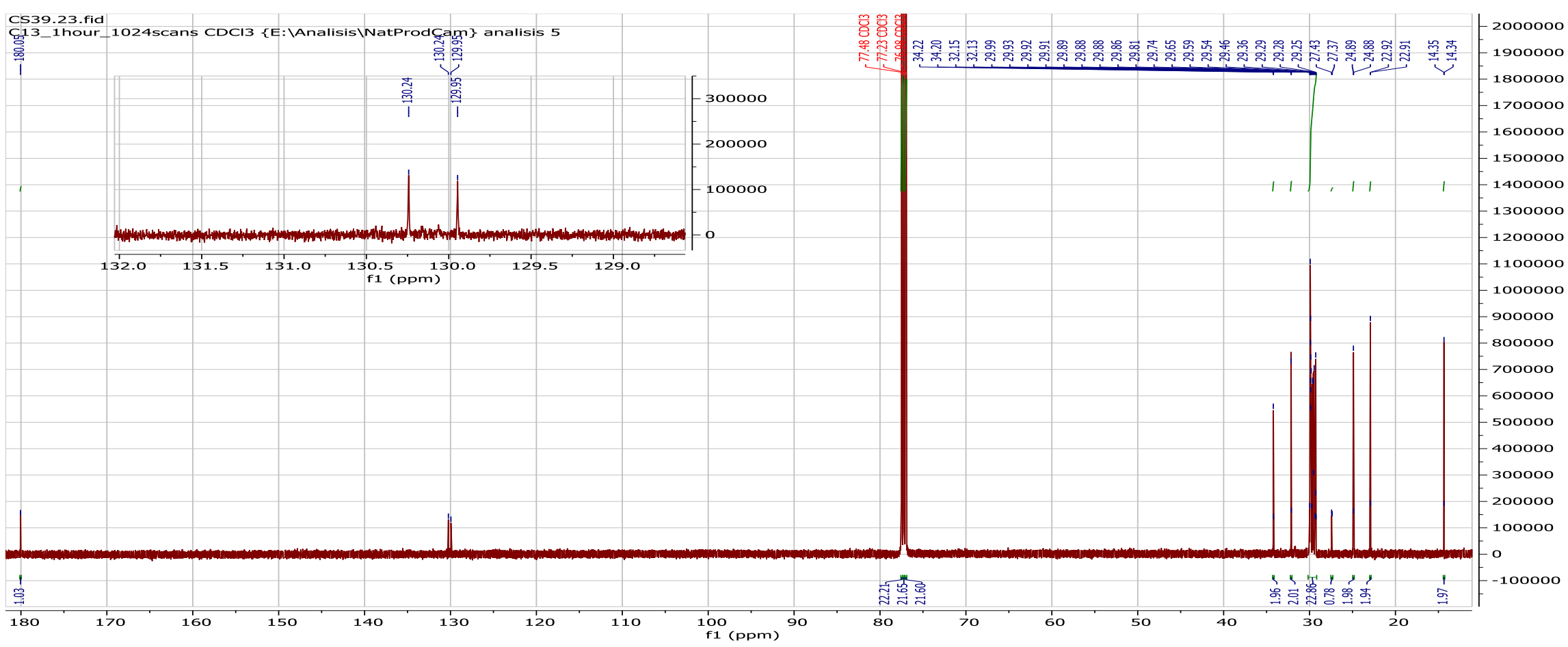

Figure S59: ^{13}C NMR spectrum ($CDCl_3$, 125 MHz) of the mixture of **15**, **16** and **17**
